# Inferring context-dependent computations through linear approximations of prefrontal cortex dynamics

**DOI:** 10.1126/sciadv.adl4743

**Published:** 2024-12-18

**Authors:** Joana Soldado-Magraner, Valerio Mante, Maneesh Sahani

**Affiliations:** ^1^ Gatsby Computational Neuroscience Unit, University College London, 25 Howland St, London W1T 4JG, UK.; ^2^ Institute of Neuroinformatics, ETH Zurich and University of Zurich, Winterthurerstrasse 190, 8057 Zurich, Switzerland.

## Abstract

The complex neural activity of prefrontal cortex (PFC) is a hallmark of cognitive processes. How these rich dynamics emerge and support neural computations is largely unknown. Here, we infer mechanisms underlying the context-dependent integration of sensory inputs by fitting dynamical models to PFC population responses of behaving monkeys. A class of models implementing linear dynamics driven by external inputs accurately captured PFC responses within contexts and revealed equally performing mechanisms. One model implemented context-dependent recurrent dynamics and relied on transient input amplification; the other relied on subtle contextual modulations of the inputs, providing constraints on the attentional effects in sensory areas required to explain flexible PFC responses and behavior. Both models revealed properties of inputs and recurrent dynamics that were not apparent from qualitative descriptions of PFC responses. By revealing mechanisms that are quantitatively consistent with complex cortical dynamics, our modeling approach provides a principled and general framework to link neural population activity and computation.

## Teaser

PFC neural responses during contextual decision-making are consistent with both recurrent and input-driven flexible computations.

## INTRODUCTION

A fascinating aspect of our daily existence is that, in a blink of an eye, we can effortlessly change our course of action, switch between tasks, or wander in between lines of thought. To achieve this flexibility, brain circuits must be endowed with mechanisms to perform context-dependent computations so that behavior is quickly adapted to each situation and the correct decisions can be taken. The mechanisms underlying this flexibility are still poorly understood.

A brain structure known to mediate flexible computations is the prefrontal cortex (PFC) (*1*). PFC is part of an extensive and highly distributed network of cortical and subcortical areas comprising the decision-making circuitry of the brain (*2*). It is involved in complex cognitive functions such as planning, selective attention, and executive control (*3*, *4*). PFC is thought to hold the representation of goals, contexts, and task rules (*5*, *6*) and in primates is required to switch behaviors according to different task instructions (*7*). Last, PFC’s crucial role in ignoring task distractors suggests that it actively filters out irrelevant information (*8*, *9*). This makes PFC of special importance for studying contextual decision-making.

Previous work suggested that flexible prefrontal computations emerge from the concerted interaction of large, interacting neural populations (*1*). Unexpectedly, during contextual decisions requiring monkeys to integrate noisy sensory information toward a choice, irrelevant information did not appear to be gated at the level of inputs into PFC. Instead, irrelevant inputs may be dynamically discarded through recurrent computations occurring within PFC. A possible mechanism for such dynamical gating was revealed by reverse-engineering recurrent neural networks (RNNs) trained to solve the same contextual decision-making task as the monkeys. The trained RNNs reproduced key features of the PFC population activity, although the networks were not explicitly designed to match the dynamics of the data. The match with the recorded data, however, was only qualitative, as these networks do not reproduce all aspects of the rich and heterogeneous responses of individual PFC neurons. It is not known whether a model explicitly designed to capture the complex PFC dynamics in its entirety would rely on the same contextual decision-making mechanism as the RNNs.

In this study, we took the approach of fitting discrete-time linear dynamical system (LDS) models directly to the PFC data, allowing us to infer interpretable low-dimensional (low-d) linear systems that approximate the neural population activity in each context. We characterized the nature of computations implemented in each context by analyzing the properties of the fitted models, whose dynamics closely matched those of the PFC population. To validate our assumption of linear dynamics, we compared the LDS to a novel low-rank factorization of the data, tensor factor regression (TFR), which can capture nonlinear dynamics. Both models performed comparably, implying that a linear model is sufficient to explain PFC activity in a given context.

We fitted different LDS model classes corresponding to different hypotheses about the nature of context-dependent computations in PFC. One class could implement context-dependent recurrent dynamics but received fixed inputs, mimicking the design of RNNs developed in past work (*1*). Another class had fixed recurrent dynamics but could implement context-dependent inputs. In both models, we inferred external input signals directly from the data. Unexpectedly, these two model classes explained the PFC responses similarly well, meaning that both contextual decision-making mechanisms are consistent with the data. Both mechanisms shared some features with the RNN solution but also differed from it in important ways, revealing previously unknown properties of PFC inputs and recurrent dynamics underlying contextual decision-making computations.

Our study emphasizes the need for quantitative modeling approaches to infer computational mechanisms from neural activity. Quantitative approaches may reveal mechanisms that appear unlikely when considering qualitative features of the neural activity alone. Quantitative models may also lead to potentially nonintuitive predictions that can be tested experimentally, as we uncover here. Our data-driven approach to analyzing neural dynamics, based on fitting LDS models to neural population responses, can be applied across different brain areas, neural datasets, and computational mechanisms, providing a general tool to test specific hypotheses about the nature of computations implemented by neural circuits.

## RESULTS

**Fig. 1.**
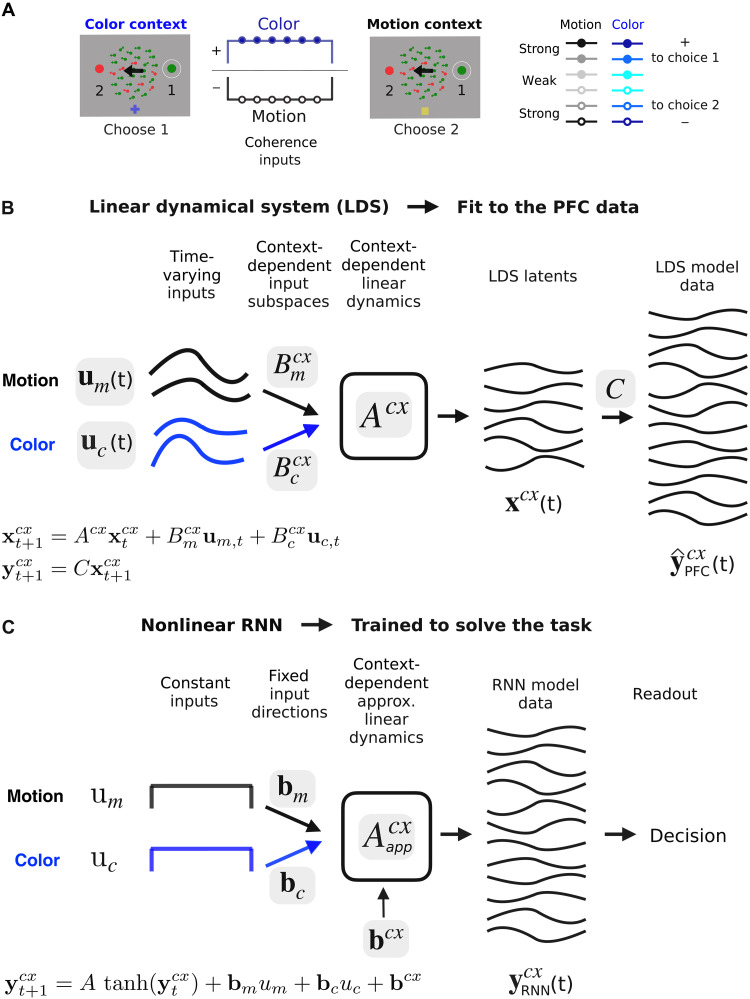
LDS model-fitting approach. (**A**) Task. Monkeys chose a target indicated by either the motion or the color coherence of a random dots display depending on context. Three coherence levels (black and blue color shades) determine the sensory evidence strength, which can point to two different choice targets (filled circles/positive values, choice 1; hollow circles/negative values, choice 2). Here, strong color coherence (green) and motion coherence (leftward motion; left arrow) point at opposite targets. In the color context, the monkey must choose the green target (on the right). In the motion context, the target on the left (red target). (**B**) LDS model fit to PFC data from both contexts with either fixed or context-dependent linear dynamics $A^{\mathrm{cx}}$ and input subspaces $B_{m,c}^{\mathrm{cx}}$. External inputs $u_{m,c}\left(t\right)$ are also learned [one for each coherence condition in (A)], are fixed across contexts, and can be time varying. These parameters determine the evolution of a low-d latent process x(t) that approximates the dynamics of the high-d PFC neural responses ${\hat{y}}_{\mathrm{PFC}}\left(t\right)$ (via the orthonormal mapping C). (**C**) Nonlinear RNNs were trained by Mante *et al.* on the same task as the monkeys. External inputs were handcrafted noisy signals with mean $u_{m,c}$ constant over time and proportional to the coherence level. Input dimensions were 1D and fixed across contexts $b_{m,c}$. A context-dependent input vector $b^{\mathrm{cx}}$ switched the dynamics of the fixed nonlinear recurrent network between two approximately linear regimes $A_{\mathrm{app}}^{\mathrm{cx}}$. A linear readout pooled network responses to generate a decision signal for training. Network population responses $y_{\mathrm{RNN}}\left(t\right)$ were only qualitatively compared to the PFC responses. Gray shadings, learned parameters from data fitting or training.

We analyzed PFC recordings from two monkeys performing a contextual version of the classic random dots motion task (*1*, *10*). The monkeys had to report the overall color or motion of the dots, depending on context (Fig. 1A). Since both types of sensory evidence were simultaneously presented, the monkeys had to actively ignore the irrelevant sensory input to form a decision based only on the relevant input. We analyzed only correct trials and focused on the random dots presentation period (750 ms), during which the motion and color evidence needed for a correct decision were presented (*1*). In the next sections, we present an in-depth analysis of the PFC data from one of the monkeys (monkey A). Findings from monkey F are presented in the Supplementary Materials and confirm the key insights gained from monkey A.

### Two classes of models implement context-dependent computation

To infer possible mechanisms underlying PFC population dynamics, we fitted several LDS models to the measured responses (Fig. 1B). Each LDS had three components: a dynamics matrix A, which determined the recurrent contribution to the evolution of a low-d “latent” activity state x(t); external motion and color inputs $u_{m}\left(t\right)$ and $u_{c}\left(t\right)$; and motion and color input subspaces $B_{m}$ and $B_{c}$, specifying dimensions along which the external inputs modulated the latent state. The dynamics matrix and the input subspaces were fixed over time, whereas the external inputs could be time varying. The condition-specific *z*-scored peristimulus time histograms (PSTHs) of individual units in PFC were then reconstructed as a linear combination of the low-d latent dynamics (via an orthonormal mapping, matrix C in Fig. 1B). Note that the population PSTHs were computed from PFC units that were not recorded simultaneously, so the inferred dynamics are based on pseudo-population data (*1*).

Critically, we fitted any given LDS model jointly to the PFC data from the two contexts, whereby only some of the model parameters varied across contexts. We considered primarily two model classes. In what we refer to as the $\left\{A^{\mathrm{cx}},B\right\}$ models, the dynamics matrix $A^{\mathrm{cx}}$ could differ across contexts (Fig. 1B, *cx* = mot/col. context), whereas the input parameters were fixed. In the $\left\{A,B^{\mathrm{cx}}\right\}$ models, on the other hand, the dynamics matrix was fixed, but the motion and color subspaces $B_{m,c}^{\mathrm{cx}}$ were allowed to vary across contexts (in both direction and norm). These two classes effectively amount to two distinct mechanisms for processing inputs flexibly across contexts.

The $\left\{A^{\mathrm{cx}},B\right\}$ models retain key properties of previously proposed RNNs (*1*). As in the RNNs, the motion and color inputs are fixed across contexts, meaning that context-dependent computations must be achieved by the recurrent dynamics (Fig. 1C). The $\left\{A,B^{\mathrm{cx}}\right\}$ models instead rely on contextually modulated inputs, a mechanism that appeared unlikely on the basis of past analyses of the PFC responses (*1*). Both model classes differ from the RNNs in several ways. First, whereas the RNNs were trained on the task, with handcrafted external inputs that were constant over time, all LDS parameters were fitted to the data (Fig. 1B, gray boxes), including the time-varying inputs $u_{m}\left(t\right)$ and $u_{c}\left(t\right)$. Second, whereas the RNNs received one-dimensional (1D) inputs, the LDS could learn multidimensional input subspaces $B_{m,c}$ and could thus produce rich activity patterns directly through the inputs (*11*, *12*). To avoid solutions that relied entirely on input driven activity, we fitted the LDS with a regularization favoring weak inputs (Materials and Methods). Activity patterns that do not directly represent the motion and color coherence, such as the integrated relevant evidence or activity related to the passage of time, would then be encouraged to emerge through the transformation of the inputs by the recurrent dynamics in all LDS models.

**Fig. 2.**
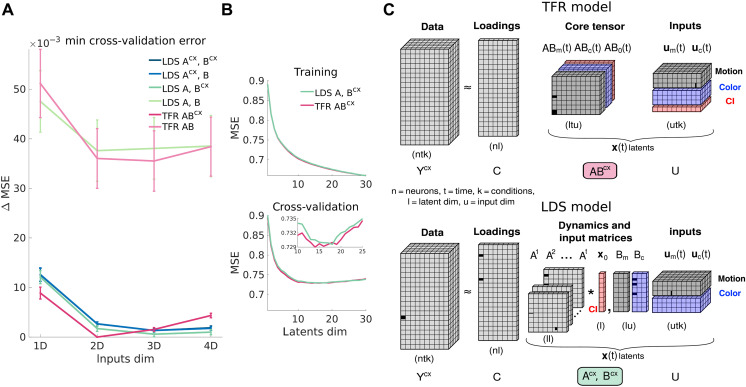
Several LDS model classes capture the PFC data equally well and comparably to a more flexible TFR model. (**A**) LOOCV (*13*) for LDS and TFR models with different input dimensionalies and contextual constraints. Shown are minimum cross-validation MSE across all latent dimensionalities, relative to the best performing model (TFR $\left\{{\mathrm{AB}}^{\mathrm{cx}}\right\}$ model with 2D inputs and 14 latents). Error bars, SEM across LOOCV folds (36 task conditions, Materials and Methods). $\left\{A^{\mathrm{cx}},B^{\mathrm{cx}}\right\}$ line is below $\left\{A^{\mathrm{cx}},B\right\}$’s. (**B**) Training and LOOCV performance for the best LDS and TFR models across different latent dimensionalities (TFR 2D $\left\{{\mathrm{AB}}^{\mathrm{cx}}\right\}$ and LDS 3D $\left\{A,B^{\mathrm{cx}}\right\}$; min LOOCV latent dim = 14 and 18). Monkey A data. (**C**) TFR model (top). The data tensor Y is factorized into three low-rank tensors, all learned. The loadings C (an orthonormal matrix) sets the rank of the factorization and maps the low-d core tensor AB into the high-d neural space. The low-d latents x(t) are generated by multiplying the core tensor and the input tensor U, which captures motion, color, and CI signals. For clarity, two indicator tensors are omitted, one recreating an LDS-like temporal convolution of the core tensor and inputs and another one that repeats the inputs across the 36 task conditions (Materials and Methods). To generate context-dependent activity $Y^{\mathrm{cx}}$, the core tensor can change across contexts ${\mathrm{AB}}^{\mathrm{cx}}$. In the LDS model (bottom), the TFR core tensor AB is replaced by a smaller set of parameters, A and B. Inputs are also repeated across task conditions. Asterisk symbol, convolution operation; $x_{0}$, initial conditions.

We found that the two LDS model classes could explain the PFC responses similarly well (Fig. 2A, $\left\{A^{\mathrm{cx}},B\right\}$ and $\left\{A,B^{\mathrm{cx}}\right\}$; cold color lines), implying that two very different mechanisms of context-dependent computation are consistent with the observed activity. A third model class that had contextual flexibility in both the recurrent dynamics and the inputs (referred to as $\left\{A^{\mathrm{cx}},B^{\mathrm{cx}}\right\}$) did not improve the fits, but given its equal performance, it could imply a mixture of the $\left\{A^{\mathrm{cx}},B\right\}$ and $\left\{A,B^{\mathrm{cx}}\right\}$ mechanisms (see Discussion). A model that could change only the initial conditions across contexts (Materials and Methods) but not the recurrent dynamics or the inputs (referred to as {A,B}) instead performed substantially worse. We estimated the dimensionality of the latent dynamics and inputs based on generalization performance using leave-one-condition-out cross-validation (LOOCV) (Fig. 2B) (*13*). We could test for generalization of the model across conditions given that the inputs were shared across several conditions, so we could generate data for the left-out conditions based on the inputs inferred from the rest of the conditions (Materials and Methods). All models performed substantially better for input dimensionalities higher than 1D (Fig. 2A), meaning that they required multidimensional input signals. The best performing LDS models required three dimensions for both the color and motion inputs. The LDS models needed between 13 and 18 latent dimensions to best fit the data (table S1), many more than the four dimensions required to describe the task (motion, color, context, and decision).

### PFC dynamics in each context are well approximated by a linear system

In the previous section, we showed that several LDS model classes capture the data equally well relative to each other. However, this finding alone does not address whether these models explain the data well in absolute terms. In this section, we demonstrate that these linear models indeed provide very accurate descriptions of the PFC responses.

First, both models closely approximated the highly heterogeneous responses of individual PFC neurons (fig. S1). The fits captured a substantial fraction of the variance in the data [27%, corresponding to mean squared error (MSE) = 0.73 on *z*-scored responses; Fig. 2B], which included poorly fit neurons with weak or sparse responses (fig. S1, firing rates <1 Hz), and were not smoothed or “denoised” (*1*).

Second, the best LDS models performed comparably to a more powerful model class that we refer to as TFR (Fig. 2, A and B, warm color lines). TFR is based on a novel low-rank model for the data that factors the data tensor into several low-d tensors, including a core tensor and an input tensor (Fig. 2C). LDS models share this factorization but impose additional constraints (Fig. 2C). The coefficients of the TFR core tensor determine how inputs at any one time affect latent state (and thus neural firing) at all other times. In an LDS, these effects are mediated by the dynamics matrix: They are causal, and influences that span multiple time steps can only do so through the linear latent dynamics. By contrast, the TFR core tensor allows each input value to have an arbitrary impact on measurements at all times. The learned pattern of input influences could be consistent with a time-varying linearization of a nonlinear dynamical system or could be inconsistent with any form of Markovian structure in the latent processes. However, the effects of different inputs in the TFR model superpose linearly, thus allowing generalization to held-out conditions in cross validation (as in the LDS model). The LDS models are nested within the TFR class (Materials and Methods), simplifying model comparisons (*14*). TFR incorporated input parameters and contextual constraints equivalent to those of the LDS (Fig. 2C and Materials and Methods). The fitted latent dimensionality was similar in the TFR and LDS fits (13D to 18D; tables S1 and S2), but the optimal input dimensionality was lower for TFR compared to the LDS fits (2D versus 3D; Fig. 2A). The extra input dimension in the LDS fits could imply limitations of the linear dynamical constraints. Beyond this difference, however, the greater flexibility in TFR provided little or no advantage to the fits (Fig. 2, A and B), confirming that linear dynamics provide an accurate description of the data.

**Fig. 3.**
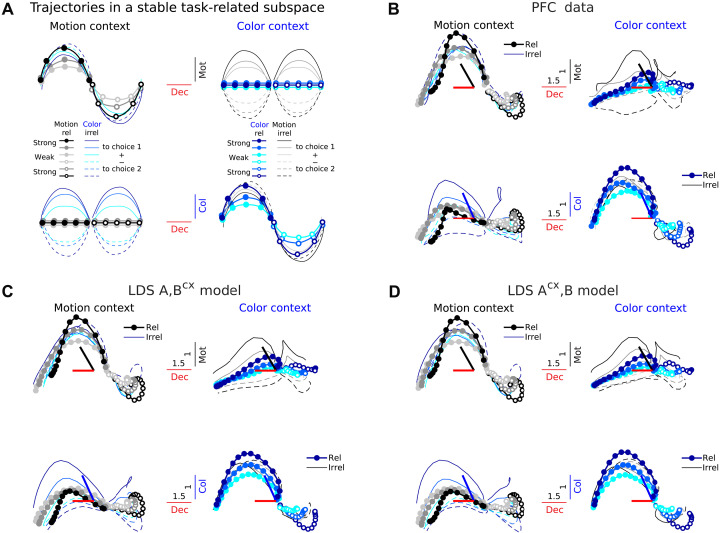
LDS models with both fixed and context-dependent input dimensions capture PFC trajectories in a fixed task-related subspace. (**A**) Population trajectories in a hypothetical subspace, fixed across contexts, capturing most variance due to motion, color, and choice. Trajectories are sorted by choice and coherence strength (as in Fig. 1A). The same trajectories are sorted twice, by either motion or color. Input dimensions encode motion and color coherence information regardless of whether this is relevant or irrelevant in a given context (thick doted versus thin lines). In contrast, the decision-related dimension encodes only the integrated relevant input in each context (*1*) (decision axes separate filled versus hollow circles, but not filled versus dashed lines). This suggests that choice-related activity emerges from the relevant input signal. Input signals are assumed transient along the input dimensions. (**B**) PFC trajectories in the task-related subspace found by Mante *et al.* using targeted dimensionality reduction (TDR) (*1*), for monkey A. The subspace captures motion, color, and choice-related variance along a set of orthonormal axes that are fixed across contexts. Colored thick bars, angle between TDR axes before orthogonalization. Numbers on bars, scaling factor to ease visualization (*1*). Trajectories are sorted by choice and motion/color coherence conditions, with color/motion conditions averaged out (*1*). (**C**) Cross-validated model trajectories (LOOCV) for the best LDS $\left\{A,B^{\mathrm{cx}}\right\}$ model (3D inputs, 18D latents) in the task-related subspace found from the PFC data [dimensions in (B) to (D) are the same]. (**D**) Same for the $\left\{A^{\mathrm{cx}},B\right\}$ model (3D inputs, 16D latents). Trajectories have been smoothed with a Gaussian filter for visualization (sliding window size, 5 bins).

Third, the best LDS models qualitatively captured salient features of the population dynamics equally well. In particular, both the $\left\{A,B^{\mathrm{cx}}\right\}$ and the $\left\{A^{\mathrm{cx}},B\right\}$ models reproduced PFC population trajectories in the activity subspace capturing most variance due to motion, color, and choice across contexts (Fig. 3) (*1*). TFR fits were comparable, both at the population (fig. S2A) and single-neuron level (fig. S1). One key implication of this finding is that the qualitative properties of population trajectories in the considered low-d activity subspace are not sufficient to distinguish between mechanisms that rely on inputs that are fixed or variable across contexts. Our quantitative model-fitting approach demonstrates that a model with flexible inputs and fixed dynamics ($\left\{A,B^{\mathrm{cx}}\right\}$) can capture the prominent context-dependence of choice-related features of the responses but at the same time generates trajectories whose input-related features appear largely stable across contexts.

To understand the mechanisms of context-dependent integration in the two model classes, below, we first separately characterize the inputs and recurrent dynamics in the $\left\{A,B^{\mathrm{cx}}\right\}$ and the $\left\{A^{\mathrm{cx}},B\right\}$ models and then ask how their combined effects can account for contextual integration in PFC. We assessed the robustness of the inferred mechanisms by fitting 100 models for each class (random initialization) with the dimensionality of inputs and latent state set by the above cross-validation results (inputs: 3D; latent: 18D and 16D for $\left\{A,B^{\mathrm{cx}}\right\}$ and $\left\{A^{\mathrm{cx}},B\right\}$; Fig. 2A and table S1).

### Input signals span curved manifolds and are largely stable across contexts

**Fig. 4.**
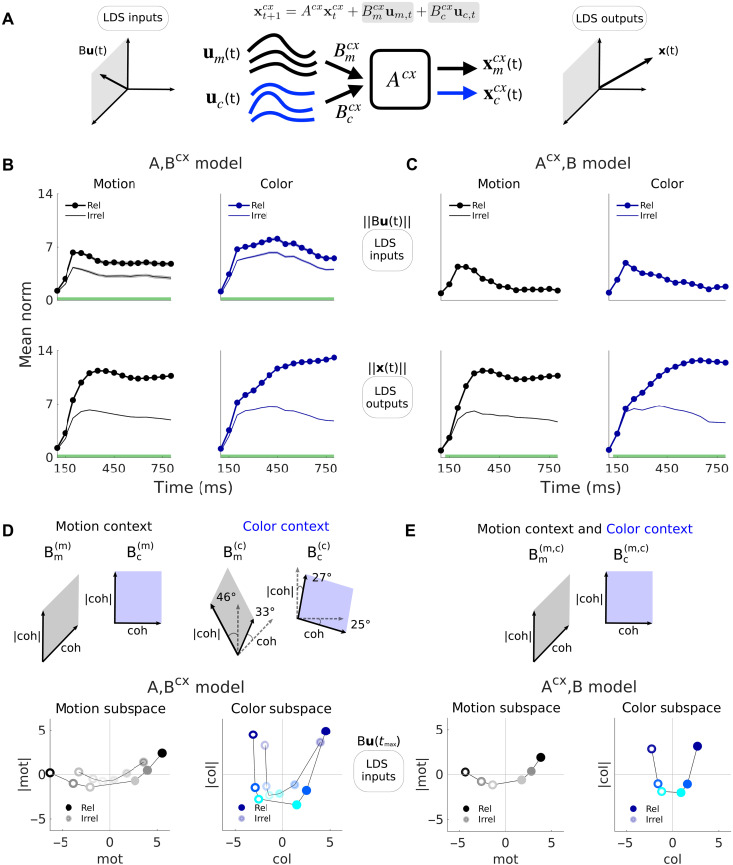
LDS inputs are integrated selectively by both models, are largely stable across contexts, and span curved manifolds. (**A**) LDS inputs and outputs. Input vectors Bu(t) are confined to the subspace spanned by the columns of B (here 2D, left). The latents or outputs x(t) span the full low-d LDS subspace (here 3D, right). (**B**) Top, external input strength over time (input norm, ‖Bu(t)‖) for the $\left\{A,B^{\mathrm{cx}}\right\}$ model in each context (relevant versus irrelevant), here shown for the strongest positive coherence. Bottom, the same but for the outputs, here generated from each motion and color input independently. To emphasize coherence-related contributions, the across-condition mean input/output has been subtracted out. Means across 100 models. Shades, SEM (not visible in the outputs). Green bars, times when relevant and irrelevant inputs/outputs are significantly different (Wilcoxon rank sum test, *P* < 0.001). (**C**) Same but for the $\left\{A^{\mathrm{cx}},B\right\}$ model. Inputs across contexts are the same, by construction, so the relevant and irrelevant traces are overlaid. (**D** and **E**) Orthonormal 2D subspaces that demix coherence and coherence magnitude information (coh and ∣coh∣). Shown are mean inputs across 100 models for all coherences (with the across-condition mean subtracted) at *t* = 250 ms (after input norm peak strength, fourth time point in Fig. 4, B and C) and projected onto the 2D coh-∣coh∣ planes. These form a curved representation of coherence information. Lines are drawn to ease visualization. For the $\left\{A,B^{\mathrm{cx}}\right\}$ model, input projections are shown onto the plane bisecting the two input planes found for each context, which were highly aligned (angles between dashed and filled lines). Color and motion planes were nearly orthogonal within each context for both models. Monkey A data.

In the models, the strength of contextual modulation can be summarized by the norm of the latent activity, which we term the model “output” ($\left\|x_{m}^{\mathrm{cx}}\left(t\right)\right\|$ and $\left\|x_{c}^{\mathrm{cx}}\left(t\right)\right\|$; Fig. 4A). We computed the output in each context by setting either the color or motion input to zero. The model output is essentially identical across model classes: It increases throughout the trial and is much larger for the relevant compared to the irrelevant input, reflecting context-dependent integration (Fig. 4, B and C, bottom, thick versus thin curves; green bars: *P* < 0.001, Wilcoxon rank sum test, *N* = 100 models). In the $\left\{A^{\mathrm{cx}},B\right\}$ model, this context dependence is due to differences in the recurrent dynamics across contexts, whereas in the $\left\{A,B^{\mathrm{cx}}\right\}$ model, it reflects contextual modulation of the strength and/or the direction of the inputs.

We computed the strength of the motion and color inputs by pooling contributions from all the corresponding input dimensions ($\left\|B_{m}^{\mathrm{cx}}u_{m}\left(t\right)\right\|$ and $\left\|B_{c}^{\mathrm{cx}}u_{c}\left(t\right)\right\|$; Fig. 4, B and C, top). Both inputs were somewhat more transient and weaker in the $\left\{A^{\mathrm{cx}},B\right\}$ compared to the $\left\{A,B^{\mathrm{cx}}\right\}$ model but were otherwise similar across the two model classes. By definition, input strength was fixed across contexts in the $\left\{A^{\mathrm{cx}},B\right\}$ model. In the $\left\{A,B^{\mathrm{cx}}\right\}$ model, the irrelevant inputs were weaker than the relevant ones but only modestly (Fig. 4B, top, thick versus thin curves, green bars: *P* < 0.001, Wilcoxon rank sum test; avg. decrease of 38±14% and 22±8% for mot and col. at *t* > 200 ms, means ± SD, *N* = 100 models).

Although our cross-validation procedure inferred 3D input subspaces, most of the inferred input variance was contained in a 2D plane (fig. S3A). LDS models with 2D and 3D inputs performed similarly (Fig. 2A and fig. S2, C and D), whereas models with 1D input performed worse (Fig. 2A and fig. S2, E and F). The input plane was spanned by dimensions that separately captured variance related to input coherence (mot and col) and coherence magnitude (∣mot∣ and ∣col∣), implying that inputs were represented along a curved 1D manifold within the plane (*15*). Such curved representations were found in both models (Fig. 4, D and E), both contexts of the $\left\{A,B^{\mathrm{cx}}\right\}$ model (Fig. 4D) and also in the PFC data (figs. S3B and S4).

The input planes were highly aligned between model classes (16° to 31°, average planes, *N* = 100 models per class; fig. S3C), an effect not expected by chance (fig. S3D). In the $\left\{A,B^{\mathrm{cx}}\right\}$ model, the motion and color planes varied across contexts but only modestly (33° ± 10 mot, 46° ± 16 ∣mot∣, 25° ± 5 col., 27° ± 9 ∣col∣ dims, means ± SD, *N* = 100 models; Fig. 4D and fig. S3C) and less than expected by chance (fig. S3D). These small changes in input direction across contexts, together with the concurrent, modest change in input strength (Fig. 4B, top), fully account for changes in the output of the $\left\{A,B^{\mathrm{cx}}\right\}$ model (Fig. 4B, bottom).

In both models, the time course (Fig. 4, B and C, top, and fig. S4) and structure of the inputs (Fig. 4, D and E) is thus relatively simple. This finding alleviates a possible confound inherent in fitting LDS with time-dependent inputs. In principle, the fitted inputs could be very rich and effectively approximate on their own the dynamics of a very complex, nonlinear dynamical system. As we retrieved inputs of much lower dimensionality than the recurrent dynamics (3D versus 16D to 18D), this scenario appears unlikely. Constraining the inputs to be fixed over time results in only a small drop in performance (fig. S5, A and B, and Supplementary Text). The observed complexity of PFC responses thus need not be inherited from the external inputs but rather can be explained as resulting from approximately linear recurrent dynamics.

### Input integration relies on high-dimensional linear dynamics

**Fig. 5.**
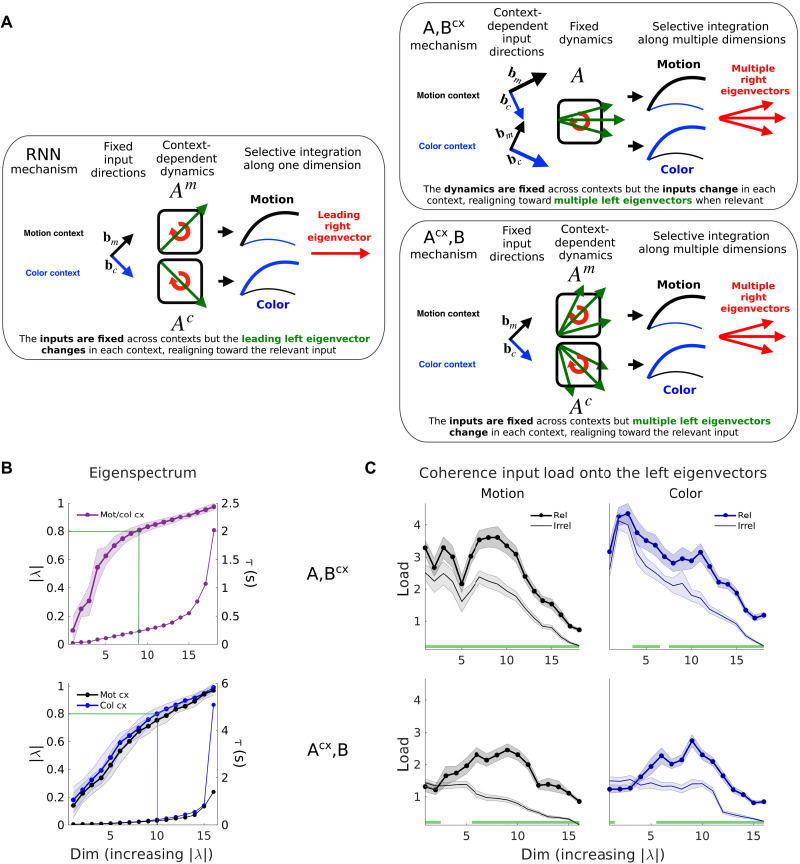
Selective integration requires multiple linear dynamics modes. (**A**) The RNN (left) had fixed input directions across contexts $b_{m,c}$. The dynamics in each context switched between two approximately linear regimes, defined by the linearized dynamics matrices $A^{m}$ and $A^{c}$. The leading left eigenvector of $A^{m,c}$ was realigned toward the relevant inputs in each context, loading them onto the slowest output mode of the dynamics (the leading right eigenvector, with associated eigenvalue close to 1), which defined a 1D integrator or line attractor (*1*). The two LDS models (right) realign either the inputs ($\left\{A,B^{\mathrm{cx}}\right\}$) or the left eigenvectors ($\left\{A^{\mathrm{cx}},B\right\}$) across contexts, loading the inputs onto multiple modes. The $\left\{A,B^{\mathrm{cx}}\right\}$ model also increases the relevant input norm (bigger input arrows, see Fig. 4B). (**B**) Average eigenvalues norm across 100 models (shades, SD). These set the rate of decay of each mode (time constant τ) and determine the stability of the dynamics (∣λ∣>1 expanding mode, ∣λ∣<1 decaying mode, ∣λ∣=1 integration mode). Slow modes have norms close to one (0.8<∣λ∣≤1, τ>224 ms, green lines; Materials and Methods). (**C**) Average coherence input loads (Materials and Methods) onto the eigenmodes of the dynamics across 100 models (shades, SEM), here shown for the strongest positive coherence inputs. The relevant input loads are significantly higher than the irrelevant loads across multiple eigenmodes (green bars, Wilcoxon rank sum test, *P* < 0.05), and the loading is large onto many modes, not just the slowest one. This is true for both models but is achieved through either a change in inputs ($\left\{A,B^{\mathrm{cx}}\right\}$) or dynamics ($\left\{A^{\mathrm{cx}},B\right\}$) across contexts [(A), right]. Monkey A data.

We analyzed the recurrent dynamics with an approach originally introduced for the nonlinear RNNs trained to solve the context-dependent integration task (*1*). Context-dependent computations in the RNNs reflect four key features of the local linear approximations of the dynamics (Fig. 5A, left). First, the (discrete-time) dynamics has one eigenvalue with norm close to one, and all other eigenvalues smaller than one, implying integration along a line attractor (*16*). Second, inputs are selected for integration by changing the direction of the leading left eigenvector of the dynamics (the “input mode” associated with the largest eigenvalue, i.e., slowest dynamics) such that it is orthogonal to the contextually irrelevant input. Third, the direction of the leading right eigenvector of the dynamics (the “output mode” associated with the largest eigenvalue), which determines the direction of the line attractor, is fixed across contexts. Fourth, the leading right and left eigenvectors have different directions, implying “non-normal” dynamics (*17*, *18*). We refer to these four features of the dynamics as the “RNN mechanism” and below compare them to the dynamics of the fitted models.

In agreement with the RNN mechanisms, both model classes inferred a largest eigenvalue with norm close to 1 (0.98±0.02, $\left\{A,B^{\mathrm{cx}}\right\}$; 0.96±0.03/0.99±0.03, mot/col. context, $\left\{A^{\mathrm{cx}},B\right\}$; means ± SD, *N* = 100 models), implying decay time constants longer than the trial duration (2.5 and 1.2/5 s). However, both models inferred an unexpectedly large number of additional eigenvalues associated with relatively slow decay (Fig. 5B; ∣λ∣>0.8, i.e., τ > 224 ms, for a 750-ms trial). Such “slow modes” were most prominent in the $\left\{A,B^{\mathrm{cx}}\right\}$ compared to the $\left\{A^{\mathrm{cx}},B\right\}$ models (55±7% versus 35±8% mot cx / 41±8% col. cx; means ± SD, *N* = 100 models). The large number of slow modes in both models suggest that PFC dynamics may be higher dimensional than predicted by the RNN mechanism.

We assessed context-dependent relations between the recurrent dynamics and the inputs by focusing on the input dimension representing coherence while ignoring the representation of coherence magnitude (Fig. 4, D and E). In the considered linear models, only the coherence component of an input can contribute to choice-dependent responses. To assess the alignment of inputs and recurrent dynamics, we first computed the “load” (the non-normalized projection; Materials and Methods) of the coherence component onto each left eigenvector at each time and then averaged over times (Fig. 5C). Consistent with the RNN mechanism, these input loads were overall larger for the relevant versus the irrelevant input in both models (Fig. 5C, green bars: *P* < 0.05, Wilcoxon rank sum test, *N* = 100 models). The load along the leading left eigenvector was close to zero for the irrelevant input, as in the RNN mechanism. Unexpectedly, however, the largest loads overall were consistently obtained for eigenvectors with intermediate eigenvalues (∣λ∣=0.7−0.8, τ=140−224ms) and thus relatively fast decay time constants (Fig. 5, B and C). Notably, the prominent differences in input loads across contexts reflect very different mechanisms in the two models: changes in the input strength and direction in the $\left\{A,B^{\mathrm{cx}}\right\}$ model and changes in the recurrent dynamics in the $\left\{A^{\mathrm{cx}},B\right\}$ model (Fig. 5A, right).

### Non-normal dynamics makes model-specific contributions to selective integration

**Fig. 6.**
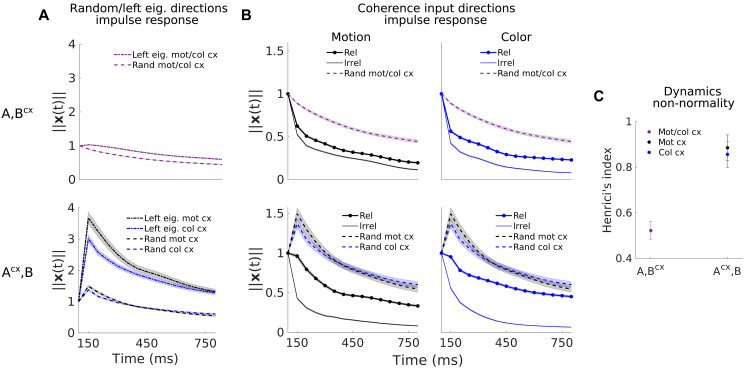
Non-normal transient dynamics contributes to selective integration in the $\left\{A^{\mathrm{cx}},B\right\}$ model. (**A**) Models $\left\{A^{\mathrm{cx}},B\right\}$ and $\left\{A,B^{\mathrm{cx}}\right\}$ mean impulse response for perturbations along random directions (dashed lines) and along the left eigenvectors (dotted lines), averaged across 100 models and across left eigenvectors or random perturbations (num pert. = num left eigv. = 16/18). This measure shows how the dynamics matrix transforms a perturbation (or input) of unit norm by tracking the state norm of the system ‖x(t)‖ over time. Note that the $\left\{A^{\mathrm{cx}},B\right\}$ system has a different impulse response for each context since the dynamics matrix A changes in each context. Shades, SEM across 100 models. (**B**) Impulse response for unit norm perturbations along the motion and color coherence input dimensions. For the $\left\{A,B^{\mathrm{cx}}\right\}$ model, the dynamics matrix is the same across contexts, and, thus, the difference in the impulse response between perturbations along the relevant and the irrelevant input dimensions arises because these input dimensions subtly change across contexts. For the model $\left\{A^{\mathrm{cx}},B\right\}$, the perturbations are applied along the same input directions across contexts since these are fixed, but the dynamics matrix changes, which causes a different transformation of the same input pulse in each context. Note that the impulse response along the input directions is substantially different from the average impulse response along random directions [dashed lines, the same as in (A)], which indicates processing selectivity of the dynamics along the input directions. Shades, SEM across 100 models. (**C**) Degree of non-normality of the two model classes (Henrici’s index, Materials and Methods). Error bars, SD across 100 models. Monkey A data.

The qualitative similarity in the eigenvalues (Fig. 5B and fig. S6A) and input loads (Fig. 5C) for the $\left\{A,B^{\mathrm{cx}}\right\}$ and $\left\{A^{\mathrm{cx}},B\right\}$ models masks a key difference in the recurrent dynamics they implement. Specifically, the two models implement dynamics with very different degrees of non-normality. We assess the strength of non-normality through one of its possible consequences, namely, the transient amplification of perturbations of the activity (Supplementary Text) (*17*, *18*). We simulated dynamics resulting from a short perturbation or pulse of activity at trial onset, along random state-space directions. For the $\left\{A,B^{\mathrm{cx}}\right\}$ model, the perturbations gradually decay over the course of the trial (Fig. 6A, top, dashed lines, average across pulses in random directions). For the $\left\{A^{\mathrm{cx}},B\right\}$ model, instead, activity following a perturbation is transiently amplified, i.e., the gradual decay is preceded by a transient increase in activity (Fig. 6A, bottom, dashed lines). For perturbations along the left eigenvectors, transient amplification is even more pronounced in the $\left\{A^{\mathrm{cx}},B\right\}$ model but still largely absent in the $\left\{A,B^{\mathrm{cx}}\right\}$ model (Fig. 6A, dotted lines). Dynamics is thus strongly non-normal in the $\left\{A^{\mathrm{cx}},B\right\}$ model, as in the RNN mechanism, but less so in the $\left\{A,B^{\mathrm{cx}}\right\}$ model (Fig. 6C and fig. S6D).

These differences in recurrent dynamics between models are also apparent in their responses to input perturbations (along the coherence dimension; Fig. 6B). In the $\left\{A,B^{\mathrm{cx}}\right\}$ model, input pulses are not transiently amplified, but rather immediately decay, whether they are relevant or not (Fig. 6B, top, thick and thin curves). In the $\left\{A^{\mathrm{cx}},B\right\}$ model, the relevant input is transiently “persistent,” because of non-normal dynamics (Supplementary Text), whereas the irrelevant input quickly decays (bottom). Also at longer timescales, the decay of a relevant input pulse is faster in the $\left\{A,B^{\mathrm{cx}}\right\}$ compared to the $\left\{A^{\mathrm{cx}},B\right\}$ model, indicating less accurate input integration. Overall, the recurrent dynamics in the $\left\{A,B^{\mathrm{cx}}\right\}$ model thus cannot sustain relevant input pulses as well as in the $\left\{A^{\mathrm{cx}},B\right\}$ model (Fig. 6B, top versus bottom thick curves). This difference explains why the $\left\{A,B^{\mathrm{cx}}\right\}$ model infers inputs that are stronger and less transient than in the $\left\{A^{\mathrm{cx}},B\right\}$ model (Fig. 4, B and C, top).

The features of the dynamics considered so far imply that the two LDS models implemented mechanisms of selection and integration that share key properties of the RNN mechanism. Like the RNNs, all LDS models ultimately relied on a context-dependent realignment between the inputs and a subset of the modes of the recurrent dynamics, either through a change of the inputs ($\left\{A,B^{\mathrm{cx}}\right\}$) or of the recurrent dynamics ($\left\{A^{\mathrm{cx}},B\right\}$). In addition, like the RNNs (*1*), the $\left\{A^{\mathrm{cx}},B\right\}$ model (but not the $\left\{A,B^{\mathrm{cx}}\right\}$ model) implemented strongly non-normal recurrent dynamics. However, while the RNNs implement only a few slow modes [and approximate a “line attractor” (*1*)], both LDS models inferred overall higher-dimensional dynamics, with a comparatively large number of slow modes. As we show below, the functional consequences of these slow modes become apparent when considering how the neural trajectories emerge from the recurrent dynamics.

### Input integration occurs in two distinct phases

Any explanation of how the neural trajectories predicted by the models emerge from the interaction of inputs and recurrent dynamics must include the properties of the right eigenvectors of the dynamics matrix. Whereas the left eigenvectors determine how inputs are coupled to the recurrent dynamics (Fig. 5C), the right eigenvectors determine “where” in activity space the inputs are mapped onto.

**Fig. 7.**
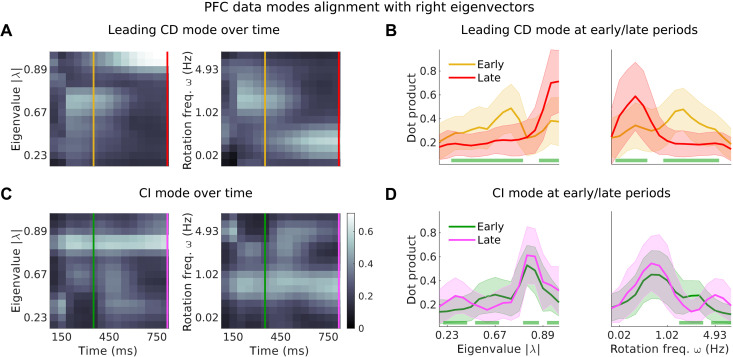
Integration of the relevant inputs occurs in two separate phases of the dynamics. (**A**) Largest variance dimension of the CD data (i.e., the leading singular vector of the data with the across-condition mean subtracted) in the motion context and, at each time step, projected onto the right eigenvectors of the dynamics from the $\left\{A^{\mathrm{cx}},B\right\}$ model (dot products for real eigenvectors, cosines of minimum subspace angles for complex conjugate pairs of eigenvectors; Materials and Methods). Left/right panel shows dot products sorted by increasing eigenvalue norm/rotation frequency of the right eigenvectors (averaged across 100 models). Yellow lines mark the early phase of the integration process, [*t* = 350 ms, the time at which the integrated motion signal in Fig. 4, (B and C), peaks and saturates]. Red lines indicate the late phase of the integration process (the last time step of the trial, where decision signals are the strongest (*1*)). (**B**) Mean distribution of alignments across 100 random models at the early and late phases [at times marked in (A)]. Shades, SD. Green bars indicate the eigenvalues along which the early and late alignment distributions significantly differ (Wilcoxon rank sum test, *P* < 0.001). (**C** and **D**) The same as (A) and (B) but for the CI data vectors (condition-averaged data vectors). Green/purple lines mark the same periods as yellow/red lines. Monkey A data.

We separately consider condition-dependent (CD) and condition-independent (CI) components of the neural trajectories. CD components were the primary focus of past accounts of these data (*1*) and late in the trial primarily capture choice-related activity. CI components, on the other hand, capture prominent structure in the trajectories that is common to all conditions and choices and appears related to the passage of time in a trial. To identify the modes of the dynamics contributing to CD or CI components, we computed the alignment between the right eigenvectors of the dynamics and the dimensions capturing most CD or CI variance at a given time in the trial (Fig. 7, $\left\{A^{\mathrm{cx}},B\right\}$ model, motion context; and fig. S7, all models and contexts). Only right eigenvectors that are well aligned with a given CD or CI dimension can contribute to response variance along that dimension.

The alignment between CD dimensions and right eigenvectors suggests that input integration occurs in two phases characterized by distinct dynamics. Early in the trial, CD responses occur primarily along right eigenvectors corresponding to modes implementing relatively fast decay and fast rotations (∣λ∣=0.7−0.8, decay time constant τ=140−224 ms, rotation frequency f>1 Hz; Fig. 7, A and B, yellow lines). Late in the trial, CD responses instead occur along right eigenvectors with very slow decay and weak or no rotations (∣λ∣>0.9, τ>475 ms, f<0.25 Hz, red lines). This transition occurs consistently across model classes, contexts, and model initializations (fig. S7). The differences in decay constants and rotational frequencies of the best aligned modes early versus late in the trial are highly significant (Fig. 7B and fig. S7B, *P* < 0.001, Wilcoxon rank sum test). These observations imply that the relevant input is initially integrated along multiple decaying and rotational modes. In line with these findings, the relevant inputs are loaded most strongly onto left eigenvectors with intermediate eigenvalues (Fig. 5C). Later in the trial, the relevant input is further integrated and maintained along at set of different, more persistent and nonrotational modes.

The CI components in the responses are mediated by modes that differ from those mediating the CD components and that appear largely fixed throughout the trial (Fig. 7, C and D, and fig. S7, C and D). At all considered times, the CI components are best aligned with a fixed set of modes that decay more slowly than the early CD-aligned modes but more quickly than the late CD-aligned modes (∣λ∣=0.8−0.9, τ=224−475 ms) and are associated with rotational frequencies that are smaller than those in early CD-aligned modes but faster than late CD-aligned modes (f=0.25−1 Hz).

The inferred modes of the dynamics can thus be grouped into three nonoverlapping sets, accounting for different components in the trajectories. The first and second sets account for early and late choice-related activity, while the third set accounts for choice-independent activity. The existence of these three different components in the PFC responses likely explains why the LDS models infer dynamics that is relatively high-dimensional and involves many modes associated with relatively slow decay.

To further validate the existence of multiple phases of the dynamics, we examined activity trajectories along dimensions aligned with the CD and CI components. We defined context-independent early and late CD dimensions (averaged across contexts), which are primarily aligned with the first and second set of dynamics modes (yellow and red lines in Fig. 7A), and a single CI dimension, which is primarily aligned with the third set of modes (green line in Fig. 7A).

**Fig. 8.**
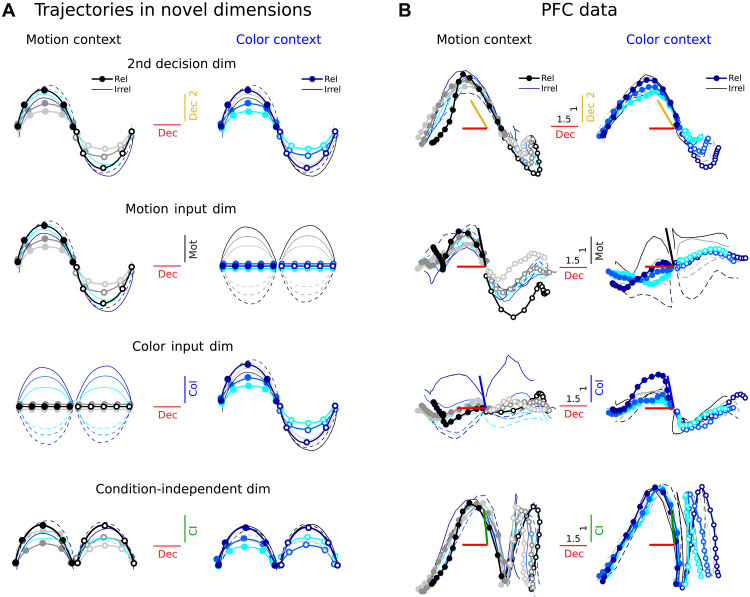
The LDS models help find multiple computational dimensions in PFC. (**A**) Expected trajectories along a hypothetical secondary decision dimension (top) that reflects transient decision signals and a dimension that captures CI signals (bottom), plotted against the evolution along a persistent decision dimension (the same plotting conventions as in Fig. 3A). Contrast these with known dimensions that reflect motion and color inputs (middle, as in Fig. 3A). The hypothetical dimensions capture additional features of the population trajectories. (**B**) PFC data trajectories from monkey A along the early integration (secondary decision), decision, and CI dimensions (defined as the top singular vector of the CD data at the early and late periods, and the CI data at the early period, Fig. 7, averaged across contexts). The same plotting conventions as in Fig. 3B. Middle panels show the trajectories along the LDS-identified input coherence dimensions, averaged across contexts and models. The data projections along them resembled the input projections found by TDR (Fig. 3B; TDR-LDS input alignments: $\left\{A,B^{\mathrm{cx}}\right\}$, mot = 55°, col. = 42°, for mean input coherence dimensions across contexts and 100 models; $\left\{A^{\mathrm{cx}},B\right\}$, mot = 44°, col. = 31°, mean dimensions across 100 models; the alignments are higher than expected by chance, fig. S6B). The across-condition mean has been subtracted to the trajectories in the middle panels to emphasize coherence-related input variance. The dimensions have been orthogonalized with a QR decomposition (*1*) (starting with decision, and then dec 2, motion, color, and CI). Colored bars show the alignments before the orthogonalization step (note that the inferred LDS coherence input dimensions are almost orthogonal to the decision dimension). Trajectories have been smoothed with a Gaussian filter for visualization (sliding window size, 5 bins).

Projections along the early CD dimension and the CI dimension reveal prominent features in the trajectories that are not apparent in other subspaces (Fig. 8, A and B), confirming their potential importance in explaining the observed dynamics. The late CD dimension approximately matched the choice axis identified by Mante *et al.* (average angular difference: 18° across contexts, much less than chance; fig. S6, B and C). Consistent with its alignment to slowly decaying modes, it captured a steady build-up of decision signals in both contexts over time (Fig. 8B, top; red dimension, dec) (*1*). The early CD dimension, which aligns to more rapidly decaying modes, instead captures transient choice-related activity that emerges early in the trial but later decays (Fig. 8B, top, yellow dimension, dec 2). Unlike activity along the input dimensions, which reflects the sign of a given input regardless of context (Fig. 8, A and B, middle; black, motion coh; blue, color coh), activity along the early CD dimension only reflects the sign of the contextually relevant input and is not modulated by the irrelevant input (Fig. 8, A and B, middle versus top). Last, projections onto the CI dimension reveal components of the responses that are common to both choices (Fig. 8, A and B, bottom). As shown above, additional dimensions capture variance due to coherence magnitude (∣col∣ and ∣mot∣ in Fig. 4, D and E, and fig. S8, A and B). All the inferred dimensions explained substantial fractions of the data variance (1 to 9%; fig. S8C) that are comparable to those captured by previously defined task-related dimensions (fig. S8D) (*1*). Overall, these projections support the existence of two phases of integration and illustrate how dimensions based on LDS fits can isolate meaningful components of the computations implemented by the neural dynamics.

### Task-optimized RNNs do not capture all features of the PFC data

The properties of inputs and dynamics in the LDS models appear to differ in several ways from those expected from a line attractor of the kind implemented by RNNs trained to solve the contextual integration task. Specifically, both LDS models infer multidimensional inputs, rely on a large number of slow dynamics modes, and process inputs in two phases (early versus late choice dimensions). However, it is not immediately clear that these features reflect meaningful differences between the linear LDS models and the nonlinear RNNs. Rather, some LDS features may reflect somewhat trivial consequences of approximating nonlinear dynamics with a linear system.

We repeated all the above analyses on simulated responses of a trained RNN and found that the highlighted features of PFC dynamics are not captured by the trained RNN (figs. S14 to S18). The LDS fits of the RNN responses infer inputs that are largely 1D (fig. S15, C to E) and do not provide any evidence of multiple phases of input integration (fig. S17). In the RNN, as in PFC, the inferred slow dynamics is not limited to a single mode, unlike in a perfect line attractor (fig. S16A and Fig. 5B). The RNN tends to implement integration along a 1D manifold that is curved (*1*), rather than perfectly straight, and thus cannot be approximated by a single linear mode. Nonetheless, the number of inferred slow modes in the RNN is substantially smaller than in PFC (20 to 22% of modes are slow on average, *N* = 100 models, fig. S16A; versus 35 to 55% in the PFC data).

The analyses of RNN responses also reiterate the challenges in establishing which of the two mechanisms implemented by the LDS models is more likely to be implemented by PFC. By design, the inputs to the RNN are not modulated by context, which matches the $\left\{A^{\mathrm{cx}},B\right\}$ models, but not the $\left\{A,B^{\mathrm{cx}}\right\}$ models. Yet, as for the PFC data, both model classes fit the RNN data equally well (fig. S14). The $\left\{A,B^{\mathrm{cx}}\right\}$ fits of the RNN responses display some idiosyncratic properties suggestive of parameter fine-tuning, like a very large number of latent dimensions (table S5) and extreme levels of non-normal amplification (fig. S16, C to E). Such fine-tuning may reflect the mismatch between the underlying mechanisms of integration. The fits of PFC responses did not display evidence of such fine-tuning, meaning that, also in this respect, both model classes are equally valid descriptions of the PFC data.

### Neural perturbations disambiguate among contextual integration mechanisms

The $\left\{A,B^{\mathrm{cx}}\right\}$ and $\left\{A^{\mathrm{cx}},B\right\}$ model classes capture the PFC data equally well, but differences in their dynamics (Fig. 6, A and C) and input properties (Figs. 4, B and C, and 6B) suggest that they could be told apart by causal perturbations of PFC circuits (Fig. 6). The effects of causal perturbations may reflect these differences directly in the measured neural population activity, without the need to fit the recorded activity with LDS models.

**Fig. 9.**
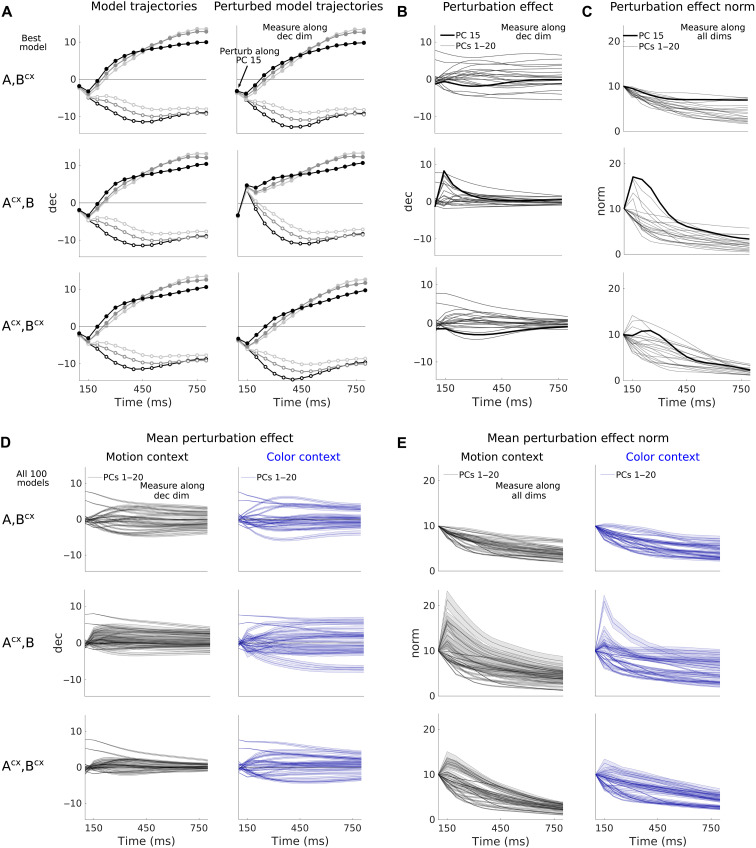
The LDS model classes predict different responses to activity perturbations. (**A**) Model-generated trajectories (left) and model-generated perturbed trajectories (right) along the decision dimension (as in Fig. 3B) for six task conditions (all motion coherence levels under the weakest positive color coherence; motion context). Perturbations are along PC 15 of the PFC data from the motion context, given at *t* = 100 ms after random dots onset (first shown time point). The perturbation vectors are of norm 10 but had only a small projection onto the decision dimension (see first time point). (**B**) Difference between the perturbed and unperturbed trajectories, which reveals the perturbation effect (thick lines, PC 15; thin lines, PCs 1 to 20). The effect is identical across the six conditions as model dynamics is linear. Best $\left\{A,B^{\mathrm{cx}}\right\}$, $\left\{A^{\mathrm{cx}},B\right\}$, and $\left\{A^{\mathrm{cx}},B^{\mathrm{cx}}\right\}$ models. (**C**) Norm of the perturbation effect over time within the entire observations space. For the PC 15 direction (thick lines), the perturbation results in strong transient amplification for model $\left\{A^{\mathrm{cx}},B\right\}$, but not for model $\left\{A,B^{\mathrm{cx}}\right\}$, and weaker amplification for the $\left\{A^{\mathrm{cx}},B^{\mathrm{cx}}\right\}$ model. (**D**) Mean perturbation effect across 100 fitted models for the first 20 PCs from the motion and color context data [as in (B); shades, 95% confidence interval]. The averages do not reveal large differences across model classes along the decision dimension. (**E**) Norm of the perturbation effect [as in (C)] across 100 models for the first 20 PCs [plotting as in (D)]. The average norm reveals the strong transient amplification properties of the $\left\{A^{\mathrm{cx}},B\right\}$ model and their absence in the $\left\{A,B^{\mathrm{cx}}\right\}$ model. Model $\left\{A^{\mathrm{cx}},B^{\mathrm{cx}}\right\}$ lies in between.

We evaluated this idea by simulating perturbations applied along the principal component (PC) dimensions of the recorded neural activity, which can be computed without any model fitting (Supplementary Text). We applied a perturbation to the activity in the first time-step of the analyzed trial-epoch and then used the models to predict the effect of the perturbation on activity in the remainder of the trial. The predicted effects of perturbations along some PC dimensions are readily visible in the activity trajectories for the best models in each class (Fig. 9A, perturbations along PC 15; left, unperturbed; right, perturbed). We quantified the effect of such perturbations by computing, at each time in the trial, the difference between perturbed and unperturbed trajectories (Fig. 9, B and C; individual lines, perturbations along different PC dimensions). We then either considered the component of this difference along the decision axis (Fig. 9B) or computed the norm of the difference in the full state space (Fig. 9C).

The perturbation effects clearly distinguish between the $\left\{A^{\mathrm{cx}},B\right\}$ and $\left\{A,B^{\mathrm{cx}}\right\}$ model classes, in particular, when considering its norm in the full state space (Fig. 9C, top two rows). Perturbations along several PC dimensions result in strong transient amplification in the $\left\{A^{\mathrm{cx}},B\right\}$ model, whereas perturbations consistently decay in the $\left\{A,B^{\mathrm{cx}}\right\}$ model. These findings are consistent with those of perturbations along random activity directions (Fig. 6A) and mainly reflect the highly non-normal dynamics of the $\left\{A^{\mathrm{cx}},B\right\}$ models (Fig. 6C). The differences between models are also apparent when considering only perturbation effects along the decision axis (Fig. 9B), but these effects overall are less pronounced than those on the full norm of the activity (Fig. 9C).

Here, we also evaluated the perturbation effects in a third model class, the $\left\{A^{\mathrm{cx}},B^{\mathrm{cx}}\right\}$ model, for which both inputs and internal dynamics can be modulated by context. Notably, these models are as good as the $\left\{A^{\mathrm{cx}},B\right\}$ and $\left\{A,B^{\mathrm{cx}}\right\}$ models in explaining the PFC data (Fig. 2A). The perturbation effects in the best $\left\{A^{\mathrm{cx}},B^{\mathrm{cx}}\right\}$ model are intermediate between those in the $\left\{A^{\mathrm{cx}},B\right\}$ and $\left\{A,B^{\mathrm{cx}}\right\}$ models, in that perturbations along multiple dimensions are amplified but less so than for the $\left\{A^{\mathrm{cx}},B\right\}$ model. The $\left\{A^{\mathrm{cx}},B^{\mathrm{cx}}\right\}$ model relies on internal dynamics (fig. S12J) and inputs (fig. S12, A to F) whose properties are intermediate between those of the $\left\{A,B^{\mathrm{cx}}\right\}$ and $\left\{A^{\mathrm{cx}},B\right\}$ model classes.

In addition to simulating perturbations of PFC activity, we also evaluated whether perturbations of the motion and color inputs could distinguish between models (fig. S13 and Supplementary Text). In simulation, we transiently switched off one of the inputs, a manipulation that experimentally could be implemented by briefly reducing the motion or color coherence to 0%. The effects of input perturbations were largest in the $\left\{A,B^{\mathrm{cx}}\right\}$ models, reflecting the stronger inferred inputs for this class compared to the $\left\{A^{\mathrm{cx}},B\right\}$ models (Fig. 4, B and C). However, input perturbations overall resulted in only subtle differences between classes, which may be challenging to validate experimentally.

Critically, the comparably larger effects of activity perturbations were highly consistent across models within a class (Fig. 9, D and E, analogous to Fig. 9, B and C, but for 100 models in each class; shading, confidence interval over models in a class). The robustness of these effects suggests that perturbations along a sufficient number of high-variance dimensions should make it possible to experimentally distinguish between linear dynamics that account for the condition-averaged trajectories equally well. The number of transiently amplified PC dimensions and the strength of this amplification may provide a quantitative measure to place PFC dynamics along a spectrum of solutions that is bookended by the $\left\{A^{\mathrm{cx}},B\right\}$ and $\left\{A,B^{\mathrm{cx}}\right\}$ models.

## DISCUSSION

The complex and highly heterogeneous activity patterns observed in prefrontal areas are thought to be critical for the computations implemented in these regions (*19*). In this study, we inferred candidate mechanisms for one such computation, contextual decision-making, by fitting interpretable LDS models directly to PFC activity. We found that two distinct mechanism of contextual-integration were consistent with the PFC responses: a switch in recurrent dynamics and a modulation of inputs. Both mechanisms required multidimensional inputs and high-dimensional integration dynamics to reproduce the complex dynamical portrait of PFC population activity. This finding stands in contrast to mechanisms inferred with alternative approaches that rely on models with limited complexity, which are easier to interpret but may miss potentially important features of the measured activity (*1*, *20*–*24*).

The first LDS mechanism is broadly consistent with past accounts of PFC responses in this task (*1*, *20*–*22*), in that the input selection relies on non-normal, context-dependent recurrent dynamics. Apart from this role in inputs selection, our analysis revealed how non-normal dynamics might additionally result in the transient amplification of relevant inputs in PFC. Non-normal transient amplification was previously proposed to be involved in the processing of external inputs (*17*, *25*–*28*) and their maintenance in working memory (*29*), in producing transient activations during movement generation (*30*), and in mediating robustness to perturbations (*31*). Our observation of two distinct stages in PFC dynamics during decision formation is evocative of the proposal that transient amplification may optimally load information onto an attractor (*27*). In contrast to such optimal loading, however, we inferred inputs that were not preferentially aligned with the most amplifying dimensions of the dynamics (fig. S6, E to G). The loading of inputs across many modes (Fig. 5C) might alternatively reflect optimal input discrimination strategies in non-normal recurrent networks (*32*).

The second LDS mechanism relies on a modest modulation of the inputs (*33*). Our LDS fits reveal the strength of top-down modulation of sensory areas that would be required to explain context-dependent responses in PFC. The inferred modulation strengths (38±14% mot, 22±8% col.; Fig. 4B) are in the range of some attentional effects observed in sensory areas (*34*, *35*), although other studies reported weaker or stronger modulation (*36*–*40*). Unlike recent modeling of sensory and prefrontal responses during contextual decisions in mice (*40*) and humans (*41*), we found that irrelevant inputs are not completely gated out before reaching PFC. Notably, not just the input strength but also its direction was modulated by context (Fig. 4D). A change in input direction could be achieved with top-down modulation if the input originated in multiple areas or subpopulations that are modulated independently (Supplementary Text). Alternatively, input amplitude and direction could both be modulated by nonlinear dynamics occurring within PFC (*21*, *23*, *42*), a possibility that we did not explicitly model here.

Our analyses provide insights into how exactly the two proposed mechanisms would have to operate to explain the PFC activity in terms of the time course and context dependence of the inputs, the properties of the internal dynamics, and the interactions of both inputs and dynamics. These distinct features result in precise, quantitative predictions that could be experimentally tested, as we discuss below. Such predictions could not have been made on the basis of a qualitative description of the data but are possible thanks to our data-driven modeling approach.

Both LDS models implemented input integration in two distinct phases, whereby choice-related signals first emerged along relatively fast decaying dimensions with rotational dynamics and then transitioned toward orthogonal dimensions with slower, nonrotational dynamics. This finding is consistent with the proposal that individual task-related signals are encoded dynamically along multiple dimensions at different timescales (*12*). Our early and late choice dimensions were well aligned with the early and middle choice dimensions in Aoi *et al.* (*12*) (35° and 22°, respectively; much more than chance, fig. S6, B and C). Our LDS fits show how these multiple choice dimensions could emerge from the interaction of inputs and recurrent dynamics and lead to a different interpretation of the underlying mechanisms. Whereas such past accounts of the data concluded that recurrent dynamics in PFC is strongly rotational late in the trial (*12*), in the LDS fits, rotational recurrent dynamics primarily shapes the early choice responses (Fig. 7). While these features of the LDS differ in several ways from dynamics in RNNs implementing approximate line attractors (*1*, *16*), in agreement with such simpler models, the late choice signals predominantly emerged along a single, context-independent integration dimension (fig. S9) (*1*).

The key features of the inferred mechanisms of context-dependent integration were consistently found across the motion and color inputs in monkey A and the motion input in monkey F (figs. S19 to S23). However, as previously reported (*1*, *12*), representations of color inputs were instead weak or absent in monkey F (fig. S20, A to D). Unlike in monkey A, activity in monkey F also revealed evidence of strong motion integration in both contexts [fig. S20, A and B; consistent with the observed choices in that monkey (*1*)]; a somewhat weaker or absent separation of integration into two phases (fig. S22); and overall stronger CI signals, which were highly aligned with choice signals (figs. S19B and S22, C and D). All these features of activity in monkey F were captured by models that ultimately relied on similar mechanisms as those in monkey A (figs. S19 to S23).

The LDS models provide several insights into the properties of potential inputs into PFC, beyond their contextual modulation. First, both mechanisms inferred multidimensional inputs carrying information about both signed coherence and coherence magnitude. The resulting curved representation of coherence, which might arise from nonlinear circuit interactions, agrees with findings in parietal and frontal areas (*11*, *12*, *15*). Notably, in our models, the different input components were inferred entirely from the data rather than being hand-designed (*12*). Second, both models inferred inputs that were somewhat transient, although the fits penalized large magnitude inputs. The inputs weakened ($\left\{A,B^{\mathrm{cx}}\right\}$ mechanism) or progressively decayed ($\left\{A^{\mathrm{cx}},B\right\}$ mechanism) late in the trial (Fig. 4, B and C). However, models with time-invariant inputs cannot be ruled out as they performed almost as well (figs. S5B and S2, G and H). This confirms that the complexity of PFC responses is well approximated by linear dynamics and not necessarily inherited from inputs with rich dynamics.

Our models provide an alternative to previously proposed approaches for inferring the properties of inputs into an area. One advantage over past approaches (*43*–*45*) is that we make minimal assumption about the properties of the inputs, like their dimensionality. Several studies have emphasized the importance of inferring inputs to understand cortical computations (*11*, *43*, *46*–*50*), but such efforts are complicated by unavoidable model degeneracies that arise when attempting to distinguish inputs from recurrent contributions without access to the upstream areas from which the inputs originate (*43*, *49*, *51*, *52*). Our finding that two fundamentally different mechanisms of input selection explain PFC responses equally well is a reflection of such degeneracy. Ultimately, the inferred inputs and choice-related signals may reflect computations distributed across several cortical areas (*2*, *51*).

Our modeling approach decomposes the dynamics of a complex system into linear parts that are easier to interpret, similar to switching LDS models (*53*). These models have proven useful to uncover dynamical motives that strongly correlate with behavior. As an example, in the hypothalamus of mice, persistent and rotational activity modes operating at different timescales were found to precisely encode social behaviors (*54*). In combination with methods from control theory, LDS models can also be used to infer inputs that are optimal for a given task, like bringing brain activity into healthy regimes in biomedical applications (*55*) or optimally configuring cortical dynamics during movement preparation (*47*, *48*, *56*). We found that our fitted LDS models are fully controllable and applied methods from control theory to identify the most amplifying dimensions of the dynamics (fig. S6, E to G) (*27*), but an exhaustive analysis of this type is beyond the scope of our study.

The LDS models explained the data essentially as well as our novel TFR model, which sets an upper bound to the goodness of fit achievable by an LDS. In PFC, intuitive linear descriptions may thus apply to all regions of state space and not only to local regions around fixed points (*1*). While we fitted activity from only a relatively short time window from each trial (the 750 ms of random dots presentation), nonlinear models may not outperform linear models in capturing cortical dynamics even on longer timescales (*57*). Nonetheless, analyses based on nonlinear models are becoming increasingly common, given their flexibility in capturing complex neural data (*43*) and in modeling biological constraints that cannot be captured by linear models (*46*) [but see (*31*)].

A crucial aspect of our data-driven modeling approach is that it is well suited to testing multiple alternative hypotheses about the mechanisms underlying the observed dynamics, but caution must be taken in selecting among competing theories when modeling complex systems like the brain (*14*, *58*). Several LDS mechanisms explained the data similarly well ($\left\{A,B^{\mathrm{cx}}\right\}$ and $\left\{A^{\mathrm{cx}},B\right\}$ models with time-varying 3D inputs, Figs. 2A and 3, C and D; 2D inputs, fig. S2, C and D; and time-constant 3D inputs, figs. S5B and S2, G and H), whereas others explained the data less well (models with time-varying 1D inputs, Fig. 2A and fig. S2, E and F) or only poorly (a {A,B} model, fully constrained across contexts, with time-varying 3D inputs, Fig. 2A and fig. S2B).

Models combining contextual modulation of both inputs and internal dynamics ($\left\{A^{\mathrm{cx}},B^{\mathrm{cx}}\right\}$) also explain the data (Fig. 2A). Notably, these models show aspects of both the $\left\{A,B^{\mathrm{cx}}\right\}$ and $\left\{A^{\mathrm{cx}},B\right\}$ models, implementing a mechanism that essentially “interpolates” between the two other solutions (fig. S12 and Supplementary Text). Given the flexibility and adaptability of PFC circuits, one could well imagine that some PFC contextual computations might be more input driven, while others might be dynamics driven; the relative contribution of both mechanisms might depend on different factors (e.g., metabolic considerations). We based our discussion on the two simpler models ($\left\{A,B^{\mathrm{cx}}\right\}$ and $\left\{A^{\mathrm{cx}},B\right\}$) since this allowed us to describe the range of solutions identified by all three model classes.

Our best LDS models share key features with mechanisms of context-dependent integration recently inferred by a study in rats (*24*), which relied on pulsatile inputs to distinguish between alternative mechanisms of input selection and integration. Similarly, the candidate mechanisms we identified ($\left\{A,B^{\mathrm{cx}}\right\}$, $\left\{A^{\mathrm{cx}},B\right\}$, and $\left\{A^{\mathrm{cx}},B^{\mathrm{cx}}\right\}$) could be distinguished by their dynamics following activity perturbations or input perturbations. The effects of such perturbations would differ between mechanisms due to their different degree of non-normality and transient amplification (Fig. 6, A and C, and fig. S12J) and their different input properties (Fig. 4, B and C, and fig. S12, A to F). The outcome of such a perturbation experiment could thus be used to place the dynamics of prefrontal circuits along the spectrum of mechanisms bookended by the extremes of the $\left\{A,B^{\mathrm{cx}}\right\}$ and $\left\{A^{\mathrm{cx}},B\right\}$ models.

Alternatively, input and recurrent contributions to the dynamics may sometimes be distinguished on the basis of the properties of trial-by-trial variability in simultaneously recorded population responses (*51*). Given that we did not have access to simultaneously recorded data, we could not use trial-by-trial residual variability to estimate properties of the dynamics that might disambiguate between such contributions. Notably, unlike modeling based on trial-by-trial variability alone (*51*), our approach based on condition-averaged responses allowed us to estimate not just the properties of the internal dynamics but also those of the external inputs. Future work may combine both approaches to account for both condition-averaged trajectories and trial-by-trial variability.

Methods for inferring neural population dynamics of the kind proposed here will likely play a key role in uncovering the neural computations underlying behavior. While abstract mental processes were originally hypothesized to reflect structural changes at the level of single neurons [Santiago Ramón y Cajal, see (*59*)], more recent evidence suggest that cognitive functions arise at the neural population level and depend critically on the ability of neural circuits to flexibly switch between dynamical regimes (*60*–*63*). Ultimately, a complete description of neural computations will also explain how neural dynamics emerges from the rich and dynamic structural components of biological circuits (*64*–*66*). The lawful characterization of population level dynamics amounts to a theoretical abstraction of the neural computations emerging from such a rich neural circuit and provides a key bridge in linking lower-level biological structure to behavior.

## MATERIALS AND METHODS

### Experimental procedures and data

#### Subjects and task

Two adult male rhesus monkeys were trained in a contextual two-alternative forced-choice visual discrimination task. The monkeys had to discriminate either the color or the motion of a random dots display based on context, which was indicated by the fixation cue (color context, blue cross; motion context, yellow square; Fig. 1A). The presentation of the random dots lasted for 750 ms, after which the monkeys had to wait for a variable delay and report their decision. This was done by saccading to one of two diametrically opposite targets, as indicated by the color or motion evidence. The strength of the evidence was modified by varying the motion and color coherence of the random dots. This was determined by the percentage of dots moving coherently or that were colored the same. Six different coherence settings were used: three strength levels and two directions. The later indicated whether the evidence was pointing toward (choice 1) or away from (choice 2) one of two choice targets—placed at the receptive field (RF) location of the recorded neurons (Fig. 1A, white circles). When the evidence pointed toward the RF of the neurons, their firing rates typically increased above baseline. Therefore, positive values were used to define the in-RF evidence. On the contrary, when the evidence pointed away from the RF of neurons, their firing rates typically decreased, and, hence, negative values were used to define the out-RF evidence. Considering all possible motion and color coherence value pairings (6 × 6), 36 different random dots configurations were presented, which defined the 36 task conditions. The motion and color evidence in a given trial could be congruent or incongruent. When incongruent, it was necessary for the monkey to ignore the irrelevant signals to perform the correct decision. All surgical and behavioral procedures conformed to the guidelines established by the National Institutes of Health and were approved by the Institutional Animal Care and Use Committee of Stanford University. For further details on the animal procedures and task, we refer to the original study (*1*).

#### Neural data

Electrophysiological recordings were performed during the task in PFC regions, likely comprising the frontal eye fields and surroundings. Both single-unit and multi-unit activity was isolated from the recordings. We referred to them as units or neurons, for simplicity. Only a few neurons were recorded simultaneously in each trial, but their activity was collected for multiple trials under the 36 different task conditions. Population responses were then constructed by pooling the condition-averaged activity of all neurons. For that, the firing rate of the neurons was computed in each trial using a 50-ms sliding square window from spike trains sampled at 1 ms. Activity was then averaged across trials under the same condition and *z*-scored, as in (*1*). However, we did not apply any smoothing to the data before fitting the models (only in the analysis, for visualization purposes). Thus, the data consisted of a pseudo-population of raw per-condition averaged PSTHs. The population size was *N* = 727 for monkey A and *N* = 574 for monkey F. We included only neurons that had recorded activity under all conditions and for all times. As in the original study, we focused our analysis on the period of random dots presentation (750 ms, from 100 ms after dots onset to 100 ms after dots offset) and we analyzed only correct trials.

### Models

#### LDS model

The LDS model considered was a non-probabilistic version of a standard LDS or state space model, with equations

$$\begin{array}{cc} x_{k}\left(t\right) & =Ax_{k}\left(t-1\right)+Bu_{k}\left(t\right)\\ y_{k}\left(t\right) & =Cx_{k}\left(t\right)+d \end{array}$$ (1)

where the vector $x_{k}\left(t\right)$ represents the latent state at time step *t* and task condition *k*, $y_{k}\left(t\right)$ are the observations (a vector containing the PFC condition-averaged PSTHs), and $u_{k}\left(t\right)$ the external input vector. The dynamics matrix A determines the transition between subsequent latent states. The initial conditions $x_{0}$ specify the latent state at *t* = 0. The matrix B defines the input dimensions. The external inputs drive the dynamical system at each time step and define input vectors Bu(t) that live in the latent subspace spanned by the columns of B. Therefore, the external inputs are assumed linearly mixed in the population at each time step. Note that the input vectors Bu(t) can point in different directions over time, but these changes are always confined within the input subspaces. Note as well that in our analysis of the inputs, we always consider the pair Bu(t) since there might be multiplicative degeneracies: A given scaling of B can always be compensated by the inverse scaling of u(t). The input term in Eq. 1 can be decomposed to make explicit its color and motion components $B_{m}u_{m}\left(t\right)+B_{c}u_{c}\left(t\right)$. The loading matrix C maps the low-d latent state onto the high-dimensional neural space. This matrix is constrained to be orthonormal, which simplifies the interpretation of the inferred dynamics given that orthonormal mappings preserve the geometrical and dynamics properties of the low-d trajectories. The constant vector d acts as a bias. This LDS model can be seen as a low-d RNN that reads out onto a high-dimensional output space.

We orthogonalized C post hoc through a similarity transformation based on the singular value decomposition (svd) of C (*67*, *68*). We first computed the svd of $C={\mathrm{USV}}^{⊺}$. We then transformed all LDS parameters $\Theta =\left(A,B,x_{0},C,d,u\right)$ using the matrix $T={\mathrm{SV}}^{⊺}$ to form $\tilde{\Theta }=\left({\mathrm{TAT}}^{-1},\mathrm{TB},Tx_{0},{\mathrm{CT}}^{-1},d,u\right)$. The loading matrix of the transformed parameters $\tilde{\Theta }$ is $\tilde{C}=U$, which has orthonormal columns.

To capture changes in activity across contexts $y_{k}^{\mathrm{cx}}\left(t\right)$, we fitted an LDS model jointly to the PFC data from each context. The model could learn independent parameters for each context (based on the data from each context) or a single parameter across contexts (using the joint data from both contexts). Both the dynamics matrix $A^{\mathrm{cx}}$ and the motion and color subspaces $B_{m,c}^{\mathrm{cx}}$ could be context-dependent (*cx* = mot or col. context). The $B_{m,c}^{\mathrm{cx}}$ matrices could have different norms, and, hence, contextual modulation of inputs could be implemented through changes in both input subspace orientation and norm. The external input signals $u_{m,c}\left(t\right)$ and the mapping C were assumed fixed across contexts.

For each motion and color input dimension, six external input time courses were learned, corresponding to the six different coherence values in the task (three strength levels and two directions). These were inferred by pooling data from all task conditions were a particular coherence level and direction was presented and, therefore, were shared across task conditions (i.e., there were 36 task conditions, but only 6 motion and 6 color input traces were inferred per input dimension). The model incorporated additional input constraints, which simplified their temporal structure and were found to improve generalization performance. Time courses were constrained to be the same for all coherence levels of the same direction. That is, a single time course was shared for positive coherences (in-RF evidence) and another one for negative coherences (out-RF evidence). The coherence strength level was learned as a scalar value that multiplied the time course $u\left(t\right)=T_{\text{in},\text{out}}\left(t\right){\mathrm{coh}}_{1,\ldots ,6}$. We also fitted a model constrained to learn fixed inputs in time, with $T_{\text{in},\text{out}}\left(1,\ldots ,t\right)=1$. The resulting input vectors (Bu) for this model also live in the input subspace defined by the input matrices B, but unlike the input vectors for the time-varying input model Bu(t), these do not move within the input subspaces over time and remain fixed throughout the trial (both in strength and direction). Orthonormal 2D subspaces that demix coherence and coherence magnitude variance (coh and ∣coh∣) were found within each inferred 3D input subspace by linearly regressing the inferred external input values against the experimental coherence values and their magnitudes (Fig. 4, D and E).

A different vector of initial conditions was also learned for each context $x_{0}^{\mathrm{cx}}$. This parameter helped the model recreate the separation of trajectories in state space found across contexts [contextual axis in the Mante *et al.* study (*1*)]. Note that this feature cannot account for the contextual differences in input integration since the model is linear, so the relationship between inputs and dynamics modes is the same everywhere in state space. A fully constrained model across contexts, with flexibility only in the initial conditions, fails to selectively integrate and poorly reproduces the data (Fig. 2A, {A,B} model, and fig. S2B). The initial conditions simply add a shift to the overall dynamics in an input-independent manner since $x_{0}$ is the same across all task conditions, so it could only capture baseline changes across contexts. This can be seen in the next equation, which illustrates the unfolding of the dynamics from the initial state and makes the dynamics and inputs convolution explicit

$$x\left(t\right)=A^{t}x_{0}+\sum_{t^{\prime }=1}^{t}\;A^{t-t\prime }\;Bu\left(t^{\prime }\right)$$ (2)

This equation also illustrates the presence of a summation degeneracy in the model. The first term defines CI effects, but these can also be captured by the input term. For this reason, in Figs. 4 and 8 and figs. S3B, S4, and S8 (and associated supplementary figures of the RNN and monkey F extended data analysis), we subtracted out the across-condition mean from the input/data trajectories along the input dimensions.

The model was implemented in Python and optimized using gradient descent (ADAM algorithm) to minimize the data reconstruction MSE,

$$\mathrm{MSE}=\frac{1}{\text{NTKC}}\sum_{t,k,\mathrm{cx}}{\left\|y_{t,k}^{\mathrm{cx}}-{\hat{y}}_{t,k}^{\mathrm{cx}}\right\|}_{2}^{2}$$ (3)

where *N* = number of neurons, *T* = trial duration, *K* = number of conditions, and *C* = number of contexts. Since the data were *z*-scored, the MSE captured the fraction of unexplained variance in the data by the model. The cost function incorporated an input norm penalty to constrain the space of possible solutions and to favor learning small inputs. This encouraged that task-related variables in the data other than the inputs, in particular, integration signals, were generated dynamically by the model. Incorporating the penalty minimally affected performance and helped provide consistent solutions across fits even when parameters were initialized at random. Therefore, we incorporated such penalty in all our model fits and randomly initialized all parameters. The resulting objective function was

$$\text{Cost}=\frac{1}{\text{NTKC}}\sum_{t,k,\mathrm{cx}}{\left\|y_{t,k}^{\mathrm{cx}}-{\hat{y}}_{t,k}^{\mathrm{cx}}\right\|}_{2}^{2}+\lambda_{\mathrm{inp}}\sum_{t,k,\mathrm{cx}}{\left\|B^{\mathrm{cx}}u_{t,k}^{\mathrm{cx}}\right\|}_{2}^{2}$$ (4)

The input penalty weight $\lambda_{\mathrm{inp}}$ was set to $10^{-5}$. Lower values resulted in inconsistent solutions across random initializations, with varying performance. Higher values ($>10^{-2}$) resulted in substantial error increases and poor convergence, especially for the $\left\{A^{\mathrm{cx}},B\right\}$ model. Inputs inferred in the range $\lambda_{\mathrm{inp}}=10^{-5}-10^{-2}$ were qualitatively similar and had comparable errors. The parameters were randomly initialized by sampling from a Gaussian distribution with zero mean and SD of 0.01. The ADAM optimizer learning rate was set to 0.009 and the rest of parameters to default. The convergence criteria was set to ΔCost $<10^{-5}$, maximum iterations to 10,000, and minimum iterations to 5000.

Note that the LDS was simply optimized to minimize the MSE of the condition-averaged PSTHs. We did not learn any observations noise model or inferred a latent state distribution, contrary to more standard formulations of the LDS, which are fully probabilistic [and typically infer Gaussian latents, or Gaussian latents combined with Poisson observations (*69*)]. We considered this a simpler case given that our data were trial averaged. Furthermore, our focus was to analyze the parameters of the dynamical model, which are part of the prior distribution over the latents in the probabilistic LDS, and not the data-corrected posterior distribution. The latents in our model are simply generated through forward prediction from the learned initial conditions $x_{0}$ based on the learned inputs and dynamics parameters (i.e., using Eq. 2).

#### TFR model

The model consists of a factorization of the data tensor structure into three main low-rank tensors

$$Y_{\mathrm{ntk}}\approx C_{\mathrm{nl}}{\mathrm{AB}}_{\mathrm{ltu}}U_{\mathrm{utk}}$$ (5)

where n= number of neurons, t= time steps, k= conditions, l= latent dimensionality, u= input dimensionality + 1D baseline. The tensor C (an orthonormal matrix) sets the rank of the factorization and maps the low-d core tensor AB into the high-dimensional neural space. The inputs tensor U captures the CD effects in the data and acts as a regressor, when this is known. When learned, as it is the case here, it is used to capture task-related variables, such as motion and color input signals. Note that similar to the LDS, these signals are assumed linearly mixed in the population at each time step.

In the previous equation, for clarity (as in Fig. 2C), we omitted an indicator tensor T that emulates the LDS-like convolution of inputs and dynamics

$$Y_{\mathrm{ntk}}\approx C_{\mathrm{nl}}{\mathrm{AB}}_{lt\prime \prime u}T_{\mathrm{tt}\prime t\prime \prime }U_{\mathrm{ut}\prime k}$$ (6)

where $T_{\mathrm{tt}\prime t\prime \prime }=\delta \left(t-t\prime =t\prime \prime \right)$. One can see how this model encompasses the LDS by writing

$${\mathrm{AB}}_{lt\prime \prime u}T_{\mathrm{tt}\prime t\prime \prime }=\left\{ \begin{array}{cc} A_{\mathrm{ll}}^{t-t\prime }B_{\mathrm{lu}} & t\geq t\prime \\ 0 & \text{otherwise} \end{array}\right.$$ (7)

where A and B correspond to the LDS dynamics and input subspace matrices, respectively. This equation shows that the TFR model has the flexibility to learn different parameters to capture the data at each point in time: In the AB core tensor, each entry along the temporal dimension can be of any value. In the LDS model, on the contrary, the parameters at each point in time are constrained to be powers of time of the dynamics matrix A (times the input matrix B). This is a subset of the possible parameters the TFR model can learn and specifies a constrained relationship between subsequent points in time that must follow linear dynamics.

The inputs incorporated constraints analogous to the LDS. First, inputs were repeated across conditions with an additional indicator tensor Q

$$Y_{\mathrm{ntk}}\approx C_{\mathrm{nl}}\;{\mathrm{AB}}_{lt\prime \prime u}\;T_{\mathrm{tt}\prime t\prime \prime }\;Q_{\mathrm{ukc}}\;U_{\mathrm{ct}\prime }$$ (8)

where c=(6×u)+1 indexes the six coherence conditions for each input dimension, plus baseline (that captures CI effects). In this way, the tensor U is designed to extract common task-related variables across conditions. Second, the temporal structure of the inputs was constrained to be the same for coherences of the same direction. For that, the input tensor U was factorized further as follows

$$Y_{\mathrm{ntk}}\approx C_{\mathrm{nl}}\;{\mathrm{AB}}_{lt\prime \prime u}\;T_{\mathrm{tt}\prime t\prime \prime }\;Q_{\text{ukcd}}\;P_{c}\;R_{\mathrm{dt}\prime }$$ (9)

where d=(2×u)+1 indexed the two possible coherence directions, per input dimension, plus the baseline.

The parameters of the TFR model can be computed by alternating the estimation of the tensors W=CAB and U. For that, one can consider the tensor unfolding $Y_{\left(n\right)\left(\mathrm{tk}\right)}$ and compute C and AB via reduced rank regression, with fixed U. Then, knowing W, the least squares estimate of U can be computed. In practice, we estimated the parameters following the same optimization procedure we used for the LDS, which provided identical results. That is, the model was implemented in Python and optimized using ADAM, with objective given by the data reconstruction MSE.

The TFR model is related to existing regression-based methods that find task-related variance in the data (*1*, *12*, *70*) but with the difference that TFR incorporates task regressors that are themselves learned from the data. Another key distinction is that TFR considers a joint factorization of the whole data tensor structure, similar to other studies (*71*), but the tensor components relate to the parameters of the task and are themselves low-d.

#### Parameter count for the LDS and TFR models

In both the LDS and the TFR model classes, the number of data points well exceeded the number of model parameters. In the LDS, we included an input penalty to the cost when fitting the models, so the effective number of parameters is even lower. Furthermore, we estimated the latent and input dimensionality using cross-validation, demonstrating that the models generalize well with the chosen number of parameters.

Monkey A: data points = 785,160; number of parameters, LDS $\left\{A,B^{\mathrm{cx}}\right\}$ = 14,605, LDS $\left\{A^{\mathrm{cx}},B\right\}$ = 13,215, TFR $\left\{{\mathrm{AB}}^{\mathrm{cx}}\right\}$ = 12,438.

Monkey F: data points = 619,920; number of parameters, LDS $\left\{A,B^{\mathrm{cx}}\right\}$ = 8603, LDS $\left\{A^{\mathrm{cx}},B\right\}$ = 8694, TFR $\left\{{\mathrm{AB}}^{\mathrm{cx}}\right\}$ = 8848.

#### RNN model

We generated data from an RNN model of the same type as used by Mante and colleagues (*1*)

$$y\left(t\right)=A\text{tanh}\left[y\left(t-1\right)\right]+b_{m}u_{m}+b_{c}u_{c}+b^{\mathrm{cx}}$$ (10)

Briefly, the model was a nonlinear RNN trained using back propagation to solve the same contextual decision-making task as the monkeys. Contrary to the LDS, the RNN was not optimized to reproduce the complex and heterogeneous responses of PFC neurons, i.e., to match PFC’s dynamics. This network was designed with the same built-in assumptions as in the original model (Fig. 1C), namely, that the external coherence input signals $u_{m}$ and $u_{c}$ were noisy but constant in time, with the mean proportional to the strength of the coherence evidence, and that these reached the circuit through two fixed input dimensions across contexts $b_{m}$ and $b_{c}$. The model had the flexibility to learn different contextual input vectors $b^{\mathrm{cx}}$, whose activation changed the dynamics of a fixed, nonlinear recurrent network (with connectivity *A*). This allowed the model to switch its state between two approximately linear regimes ($A_{\mathrm{app}}^{\mathrm{cx}}$= $A_{\mathrm{app}}^{\mathrm{mot}}$/$A_{\mathrm{app}}^{\mathrm{col}}$), performing different computations in each context, namely, selecting the contextually relevant input signals for integration toward a choice and dynamically discarding the irrelevant ones. In the original study, the RNN population activity $y_{\mathrm{RNN}}^{\mathrm{cx}}$ was analyzed and qualitatively compared with the PFC activity, revealing some shared features that were suggestive of a common contextual-integration mechanism between PFC and the network. The network could be “reverse engineered” to understand the mechanism underlying such computation by linearizing the dynamics around the identified fixed points of the system [obtaining different local dynamics matrices $A_{\mathrm{app}}^{\mathrm{mot}/\mathrm{col}}$, which, however, were similar in dynamics and could be averaged (*1*)]. In this work, we instead focused on analyzing the properties of LDS models fit to the RNN population activity $y_{\mathrm{RNN}}^{\mathrm{cx}}$ (the *z*-scored condition-averaged responses, as in the PFC data, but from 100 RNN units) and recovered one or two dynamics matrices ($A^{\mathrm{mot}/\mathrm{col}}$ in the $\left\{A,B^{\mathrm{cx}}\right\}$ model; $A^{\mathrm{mot}}$ and $A^{\mathrm{col}}$ in the $\left\{A^{\mathrm{cx}},B\right\}$ model) that approximated the global dynamics of the RNN population in both contexts. For further details on the RNN training and analysis, we refer to the original study (*1*).

### Dynamics analysis

#### Eigenspectrum and time constants

The eigenspectrum of the LDS dynamics matrices contains both real and imaginary eigenvalues (fig. S6A), which come in complex-conjugate pairs

$$\begin{array}{c} \lambda =\lambda_{\mathrm{re}}+\lambda_{\mathrm{im}}i\\ \lambda^{†}=\lambda_{\mathrm{re}}-\lambda_{\mathrm{im}}i \end{array}$$ (11)

In discrete time dynamical systems, the absolute value of the eigenvalues determines the rate of decay or growth of each dynamic mode (*72*) (in continuous time models, instead, this is controlled by the real part of the eigenvalues). Modes are stable if they either decay or persist

$$\begin{array}{cc} \lambda & \leq 1\;\;\forall \lambda \;\text{real}\\ |\lambda |=\sqrt{\lambda_{\mathrm{re}}^{2}+\lambda_{\mathrm{im}}^{2}} & \leq 1\;\;\forall \lambda \;\text{complex} \end{array}$$ (12)

The slower the decay, the slower or more persistent a given mode is, and the greater input information is preserved along it. The time constant measures the time at which the initial state will have decayed by 37% (1/e = 0.37) along a given mode. Considering that each time step is 50 ms (the data binning size), the time constant is computed as

$$\begin{array}{c} x_{t}={|\lambda |}^{t}x_{0}\\ \left(1/e\right)x_{0}={|\lambda |}^{t}x_{0}\\ \tau =\frac{\text{log}\left(1/e\right)}{\text{log}|\lambda |}50 \end{array}$$ (13)

We classify a mode as slow if it has a norm close to one, that is, if ∣λ∣>0.8. This corresponds to a decay time constant of τ>224 ms, which encompasses approximately a third of the trial duration. Given that the inferred external inputs in the two models are strong for the first third of the trial (Fig. 4, A and B), inputs mapped onto such slow modes largely persist until the end of the trial, albeit with some decay for modes ∣λ∣=0.8−0.9. In particular, by the second third of the trial, inputs would have decayed by at most 37%. We consider the slowest modes to have ∣λ∣>0.9 and time constant τ>475 ms. These are strongly persistent and preserved most input information until the end of the trial. The relatively fast decaying modes (∣λ∣=0.7−0.8, τ=140−224 ms) are somewhat persistent but lose most input information by the end of the trial.

Many of the eigenvalues were imaginary, indicating the presence of rotational dynamics in the data (*61*). Some of the eigenvalues were negative, which also indicate the presence of oscillations (*55*). A few models identified slightly unstable eigenmodes (with eigenvalue norm slightly bigger than 1), but this is expected when learning from finite trial lengths and limited data samples (*68*). However, the models inferred from monkey F data, in particular for the $\left\{A,B^{\mathrm{cx}}\right\}$ model, seemed to use instability properties of the dynamics to capture specific features of the data (fig. S21, A and D).

#### Rotational dynamics measure

As mentioned above, the existence of complex eigenvalues indicates the presence of rotational dynamics in the data. Rotations are confined to the planes defined by pairs of complex-conjugate eigenvectors, with directions spanned by the real and imaginary components of the vectors. On each plane, state trajectories are shaped by the rotation matrix J, which derives from the dynamics matrix A expressed in the Jordan normal form (*72*). As an example, for a 2D system with two distinct complex eigenvalues, which come as a complex-conjugate pair $\lambda -\lambda^{†}$ (Eq. 11), if we consider their phase plane representation in polar coordinates

$$\lambda_{\mathrm{re}}=|\lambda |\text{cos}\omega \;\lambda_{\mathrm{im}}=|\lambda |\text{sin}\omega$$ (14)

where

$$\omega =\text{arctan}\left(\frac{\lambda_{\mathrm{im}}}{\lambda_{\mathrm{re}}}\right)$$ (15)

the rotation matrix J is given by

$$J=\left[\begin{array}{cc} \lambda_{\mathrm{re}} & -\lambda_{\mathrm{im}}\\ \lambda_{\mathrm{im}} & \lambda_{\mathrm{re}} \end{array}\right]=|\lambda |\left[\begin{array}{cc} \text{cos}\omega & -\text{sin}\omega \\ \text{sin}\omega & \text{cos}\omega \end{array}\right]$$ (16)

Rotations evolve in time following powers of J, with amplitude over time (the rate of decay or growth) given by the absolute value of the eigenvalues, and with rotation frequency ω

$$J^{t}={\left(|\lambda |[\begin{array}{cc} \text{cos}\omega & -\text{sin}\omega \\ \text{sin}\omega & \text{cos}\omega \end{array}]\right)}^{t}={|\lambda |}^{t}\left[\begin{array}{cc} \text{cos}\omega t & -\text{sin}\omega t\\ \text{sin}\omega t & \text{cos}\omega t \end{array}\right]$$ (17)

Note that the frequency increases when the ratio $\frac{\lambda_{\mathrm{im}}}{\lambda_{\mathrm{re}}}$ is big. The rotation frequency ω is given in rad/s and f=ω/(2π) in Hz. Since the data were downsampled at 20 Hz (50-ms bins), the frequency is given by f=20ω/(2π) in Hz (the value reported in Fig. 7). For real modes, the rotation frequency is zero.

#### Non-normality measure

The Henrici’s index measures the degree of non-normality of the dynamics and is given by (*73*)

$$H=\frac{\sqrt{{\left\|A\right\|}_{F}^{2}-\sum_{i}{|\lambda_{i}|}^{2}}}{{\left\|A\right\|}_{F}}$$ (18)

This is a normalized metric with values between 0 and 1, with 0 indicating that the system is normal and 1 that is maximally non-normal. A system is normal when its dynamics can be described with an orthonormal eigenvector basis. A system is non-normal when its eigenvectors do not necessarily form an orthonormal basis, and the transformation to eigenvector coordinates may involve a strong distortion of the phase space (*73*). In normal linear networks, the network responses are explained with a linear combination of exponentially decaying modes (if the system is stable), with timescales defined by the corresponding eigenvalue (Eq. 13). In non-normal stable networks, however, more complex patterns can emerge, which often involve transient responses where the network activity temporarily grows, but eventually decays as in normal systems.

A crucial property of non-normal systems is that they have different left and right eigenvectors

A=RΛL (19)

with $L=R^{-1}$, whereas for normal systems $L=R^{†}$ († = conjugate transpose). This non-normal property allowed the RNN trained by Mante *et al.* to change the leading left eigenvectors across contexts while keeping the right eigenvectors pointing in the same direction (*1*).

#### Input loads

The input loads are defined by the non-normalized projection of the coherence inputs onto the left eigenvectors, averaged across all time steps. To compute the input loads, we start by expressing the latents in the left eigenvectors basis

$$\begin{array}{c} x\left(t\right)=Ax\left(t-1\right)+Bu\left(t\right)\\ x\left(t\right)=\left(R\Lambda L\right)x\left(t-1\right)+Bu\left(t\right)\\ Lx\left(t\right)=\Lambda Lx\left(t-1\right)+\mathrm{LB}u\left(t\right) \end{array}$$ (20)

where we have taken the eigendecomposition of the matrix A, with R containing the right eigenvectors in its columns and $L=R^{-1}$ the left eigenvectors in its rows. We have then left-multiplied by L. Defining α(t)=Lx(t), we obtain

α(t)=Λα(t−1)+LBu(t) (21)

The evolution of the latents in this basis is independent, that is, decoupled from one another—given that the matrix Λ is diagonal. Unrolling this equation, in time, we obtain

$$\alpha \left(t\right)=\Lambda^{t}Lx_{0}+\sum_{t\prime =1}^{t}\;\Lambda^{t-t\prime }\mathrm{LB}u\left(t\prime \right)$$ (22)

As the eigenmodes are independent, we can write down a set of uncoupled equations that describe the evolution of each eigenmode, one for each entry of the vector α, given by $\alpha_{l}$ with l indexing the latent dimension

$$\alpha_{l}\left(t\right)=\lambda_{l}^{t}l_{l}^{⊺}x\left(0\right)+\sum_{t\prime =1}^{t}\lambda_{l}^{t-t\prime }l_{l}^{⊺}Bu\left(t\prime \right)$$ (23)

and $l_{l}$ being the lth left eigenvector. The input “load” is defined by the last term of the summation, which corresponds to the non-normalized projection of the inputs onto the left eigenvectors (given that neither the input vectors nor the left eigenvectors are unit norm)

$${\text{load}}_{l}\left(t\right)=l_{l}^{⊺}Bu\left(t\right)$$ (24)

This term specifies how strongly the inputs are mapped onto the dynamic modes at each time step *t*, before being processed by the dynamics (i.e., in this basis, before being scaled by λ). The extent to which the inputs are mapped or “loaded” onto each mode depends on the alignment between the input vectors and each left eigenvector, as well as the norm of both vectors. For each pair of complex modes, the load is given by

$${\text{load}}_{l-l^{†}}\left(t\right)=2\|ℜ\left\{l_{l}^{⊺}\right\}Bu\left(t\right)ℜ\left\{r_{l}\right\}-ℑ\left\{l_{l}^{⊺}\right\}Bu\left(t\right)ℑ\left\{r_{l}\right\}\|$$ (25)

where ℜ{.} and ℑ{.} take the real and imaginary components of their arguments. The rationale for the expression above comes from the following. For complex modes, Eq. 24 contains imaginary numbers since the left eigenvectors are complex, so we cannot interpret the loads in this basis. However, we can do it in the original state vector basis x(t), which is real. To change basis, we use α(t)=Lx(t) and express x(t) as a linear decomposition of the state along each right eigenvector dimension. The coefficients of the linear decomposition are given by $\alpha_{l}\left(t\right)$, which contains the input loads

$$x\left(t\right)=R\alpha \left(t\right)=\sum_{l}\alpha_{l}\left(t\right)r_{l}$$ (26)

We can now make explicit the contribution due to real eigenmodes and complex eigenmodes, which come in complex conjugate pairs ($l-l^{†}$)

$$x\left(t\right)=\sum_{l-l^{†},\mathrm{img}}\left[\alpha_{l}(t)r_{l}+\alpha_{l}^{†}(t)r_{l}^{†}\right]+\sum_{l,\text{real}}\alpha_{l}\left(t\right)r_{l}$$ (27)

Because of the complex conjugacy, the imaginary numbers end up cancelling out in the summation, and only real terms survive. This is why in this basis, the state vector **x**(*t*) is real. In particular, the way the complex roots end up contributing to the state dynamics is given by their real and imaginary parts. This is because for each pair of complex conjugate roots, two complementary real solutions exist, which are given by the sum and difference modes $\alpha_{l^{\pm }}\left(t\right)$

$$\begin{array}{c} \alpha_{l^{+}}\left(t\right)=\frac{1}{2}\left(\alpha_{l}\left(t\right)+\alpha_{l}^{†}\left(t\right)\right)=ℜ\left\{\alpha_{l}\left(t\right)\right\}\\ \alpha_{l^{-}}\left(t\right)=\frac{1}{2i}\left(\alpha_{l}\left(t\right)-\alpha_{l}^{†}\left(t\right)\right)=ℑ\left\{\alpha_{l}\left(t\right)\right\} \end{array}$$ (28)

This can be seen by expanding the complex term in the state equation

$$\begin{array}{cc} \alpha_{l}\left(t\right)r_{l}+\alpha_{l}^{†}\left(t\right)r_{l†} & =\left(ℜ\left\{\alpha_{l}\left(t\right)\right\}+\mathrm{iℑ}\left\{\alpha_{l}\left(t\right)\right\}\right)\left(ℜ\left\{r_{l}\right\}+\mathrm{iℑ}\left\{r_{l}\right\}\right)\\ & +\left(ℜ\left\{\alpha_{l}\left(t\right)\right\}-\mathrm{iℑ}\left\{\alpha_{l}\left(t\right)\right\}\right)\left(ℜ\left\{r_{l}\right\}-\mathrm{iℑ}\left\{r_{l}\right\}\right)\\ & =2ℜ\left\{\alpha_{l}\left(t\right)\right\}ℜ\left\{r_{l}\right\}-2ℑ\left\{\alpha_{l}\left(t\right)\right\}ℑ\left\{r_{l}\right\}\\ & =2\left(\alpha_{l^{+}}\left(t\right)ℜ\left\{r_{l}\right\}-\alpha_{l^{-}}\left(t\right)ℑ\left\{r_{l}\right\}\right) \end{array}$$ (29)

Thus

$$\begin{array}{cc} x\left(t\right) & =\sum_{l-l^{†},\mathrm{img}}2\left(ℜ\left\{\alpha_{l}\left(t\right)\right\}ℜ\left\{r_{l}\right\}-ℑ\left\{\alpha_{l}\left(t\right)\right\}ℑ\left\{r_{l}\right\}\right)\\ & +\sum_{l,\text{real}}\alpha_{l}\left(t\right)r_{l} \end{array}$$ (30)

To understand how the inputs are loaded at each time step *t* into the dynamic modes to affect the latent state, we focus on the last term of the summation in the equation $\alpha_{l}\left(t\right)$ (Eq. 23), as we did before

$$x{\left(t\right)}_{\text{input}}=\sum_{l-l^{†},\mathrm{img}}2\left(ℜ\left\{l_{l}^{⊺}\right\}Bu\left(t\right)ℜ\left\{r_{l}\right\}-ℑ\left\{l_{l}^{⊺}\right\}Bu\left(t\right)ℑ\left\{r_{l}\right\}\right)+\sum_{l,\text{real}}l_{l}^{⊺}Bu\left(t\right)r_{l}$$ (31)

The last term contains the input loads along each real mode, $l_{l}^{⊺}Bu\left(t\right)$, which gives Eq. 24. This value indicates how much of the input is mapped along each right eigenvector direction $r_{l}$ (for *l* real). Thus, considering only this term, the latent state vector is reconstructed with a linear combination of real right eigenvectors, weighted by the input loads. Note, however, that the right eigenvectors are not orthogonal, so the result of the sum could be nontrivial, if, for instance, some of this vectors cancel out or give rise to transient amplification (Supplementary Text). The total input contribution or load along each direction $r_{l}$ is thus given by the norm of the vector $l_{l}^{⊺}Bu\left(t\right)r_{l}$. Since the real right eigenvectors are normalized, this is equal to $l_{l}^{⊺}Bu\left(t\right)$, which gives Eq. 24. Similarly, the load for each complex conjugate pair of modes is given by the norm of the vector $2\left(ℜ\left\{l_{l}^{⊺}\right\}Bu\left(t\right)ℜ\left\{r_{l}\right\}-ℑ\left\{l_{l}^{⊺}\right\}Bu\left(t\right)ℑ\left\{r_{l}\right\}\right)$, which gives Eq. 25. This vector lives within the 2D plane spanned by the real and imaginary components of the complex-conjugate right eigenvector pairs.

To compute the loads in Fig. 5C, we use the inferred inputs for the largest motion and color positive coherence values and project them along the coherence dimension. Thus, the loads are computed using the coherence component of Bu(t), for all times and all 100 randomly initialized models, and then averaged across time and models. For complex modes, the same load is shared across both complex conjugate pairs and is computed using Eq. 25.

#### Most amplifying dimensions

The most amplifying modes were found following (*27*), by computing the Observability Gramian Q and its associated eigenvectors. The most amplifying modes are defined by the eigenvectors with the largest associated eigenvalues. We computed the Observability Gramian by solving the following discrete-time Lyapunov equation

$$A^{⊺}\mathrm{QA}-Q+C^{⊺}C=0$$ (32)

where A is the LDS models dynamics matrix and C is the loading matrix. We considered only stable models (*27*), which, in our case, were 90% of the 100 $\left\{A,B^{\mathrm{cx}}\right\}$ models and 85% (mot cx), 60% (col cx) of the $\left\{A^{\mathrm{cx}},B\right\}$ models in monkey A.

#### Models constrained to have normal dynamics

To learn models with normal dynamics, we included the penalty ${\mathrm{AA}}^{⊺}-A^{⊺}A$ in our cost. To ensure that normality in the latent dynamics was accurately reflected in the reconstructed neural dynamics, we fixed C to the orthonormal-column matrix identified in the standard fits. We enforced normality with different penalty weights ($\lambda_{\mathrm{dyn}}=1$ and $\lambda_{\mathrm{dyn}}=1e5$). These analyses are discussed in Supplementary Text and fig. S5 (E to G).

### Additional analysis methods

#### Alignment metrics

We report alignments between different dimensions using either dot products or angles (in degrees). When computing alignments between a given vector and complex eigenvectors, we consider the plane spanned by the real and imaginary components of the pair of complex conjugate eigenvectors and compute the minimum subspace angle between the vector and the plane.

#### Statistical tests

To test for statistically significant differences between distributions, such as the relevant versus irrelevant load distributions in Fig. 5C, we used a Wilcoxon rank sum test with significance level (*P* values) of *P* < 0.001 (Figs. 4, B and C, and 7B and fig. S7B and associated supplementary figures of the RNN and monkey F extended data analysis) or *P* < 0.05 (Fig. 5C and associated supplementary figures of the RNN and monkey F extended data analysis; also fig. S17). This is a two-sided rank sum test of the null hypothesis that two independent samples come from distributions with equal medians.

## Supplementary Materials

### This PDF file includes:

Supplementary Text Figs. S1 to S24 Tables S1 to S5 References
