# Supplementary material for: Inferring context-dependent computations through linear approximations of prefrontal cortex dynamics
Source: Sci Adv. 2024 Dec 18;10(51):eadl4743. doi: 10.1126/sciadv.adl4743 (PMC11654703; doi:10.1126/sciadv.adl4743)
Supplement: Supplementary file 1 — Supplementary Text Figs. S1 to S24 Tables S1 to S5 References [file sciadv.adl4743_sm.pdf]

Science Advances
AAAS

# Supplementary Materials for

## Inferring context-dependent computations through linear approximations of prefrontal cortex dynamics

Joana Soldado-Magraner *et al.*

Corresponding author: Joana Soldado-Magraner, jsoldadomagraner@cmu.edu

*Sci. Adv.* **10**, eadl4743 (2024)
DOI: 10.1126/sciadv.adl4743

**This PDF file includes:**

Supplementary Text
Figs. S1 to S24
Tables S1 to S5
References

# Supplementary Figures 1–13

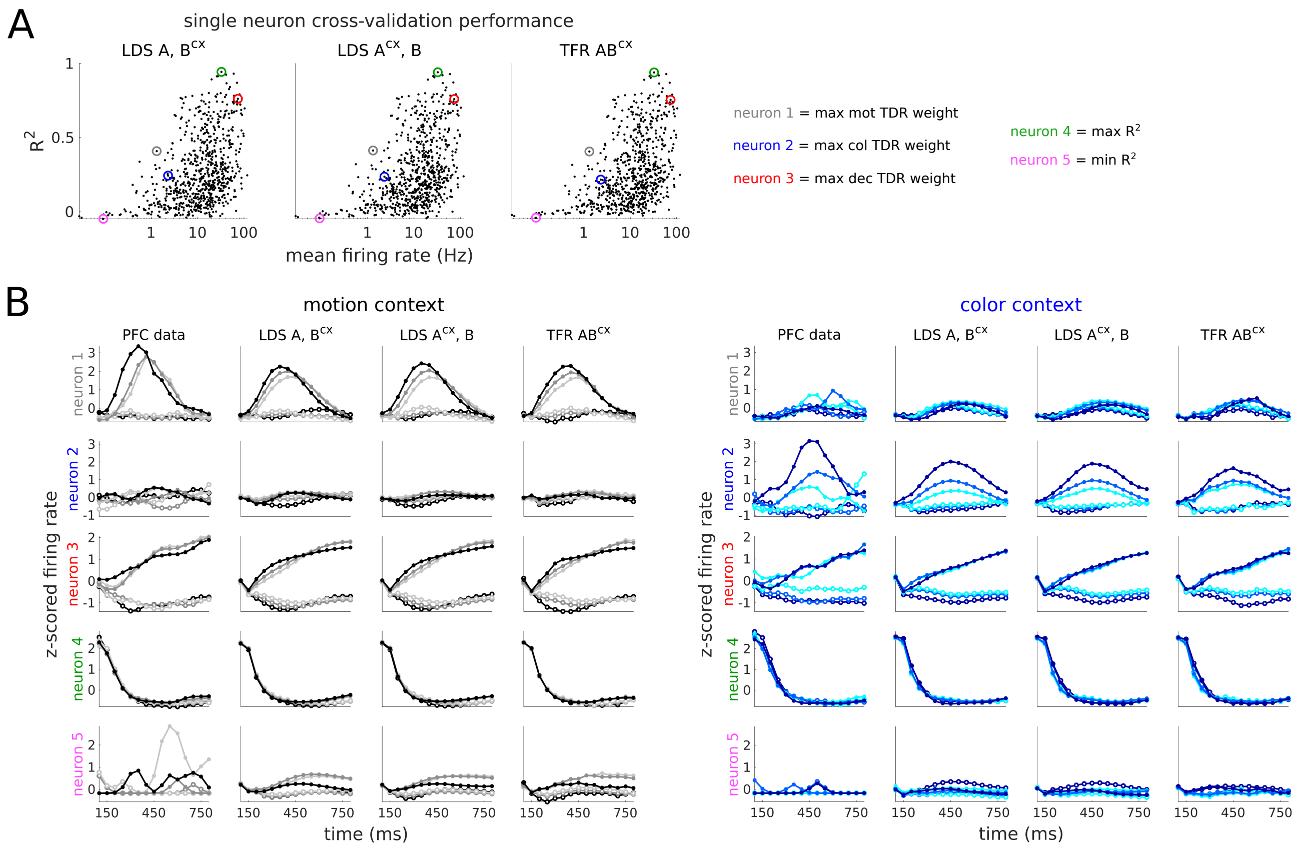


**Fig. S1**. **Individual neuron performance for the best LDS and TFR models.** (**A**) LDS and TFR models $R^2$ for all individual neurons, sorted by their mean firing rate as in Aoi et al. [12]. Highlighted in colors are five example neurons. Three of them have maximum selectivity to either motion, color or decision (in grey, blue, red), as measured by their weight onto the motion, color and decision population vectors found using targeted dimensionality reduction (TDR) [1]. The two other neurons were selected based on model performance (best neuron captured, max $R^2$, in green, and worse neuron captured, min $R^2$, in pink). (**B**) PFC data and LDS/TFR models cross-validated PSTHs for the 5 example neurons. The PSTHs are computed from z-scored data and model responses sorted by the relevant coherence value in each context (motion in the motion context, left, and color in the color context, right) and averaged across irrelevant coherence conditions, as in Mante et al. [1]. Color shades and filled/hollow circles indicate the strength and direction of the coherence evidence, respectively (same notation as in Fig. 1A). PFC data responses have been smoothed with a Gaussian kernel ($\sigma$=40ms) for visualization [1]. Data is from monkey A.

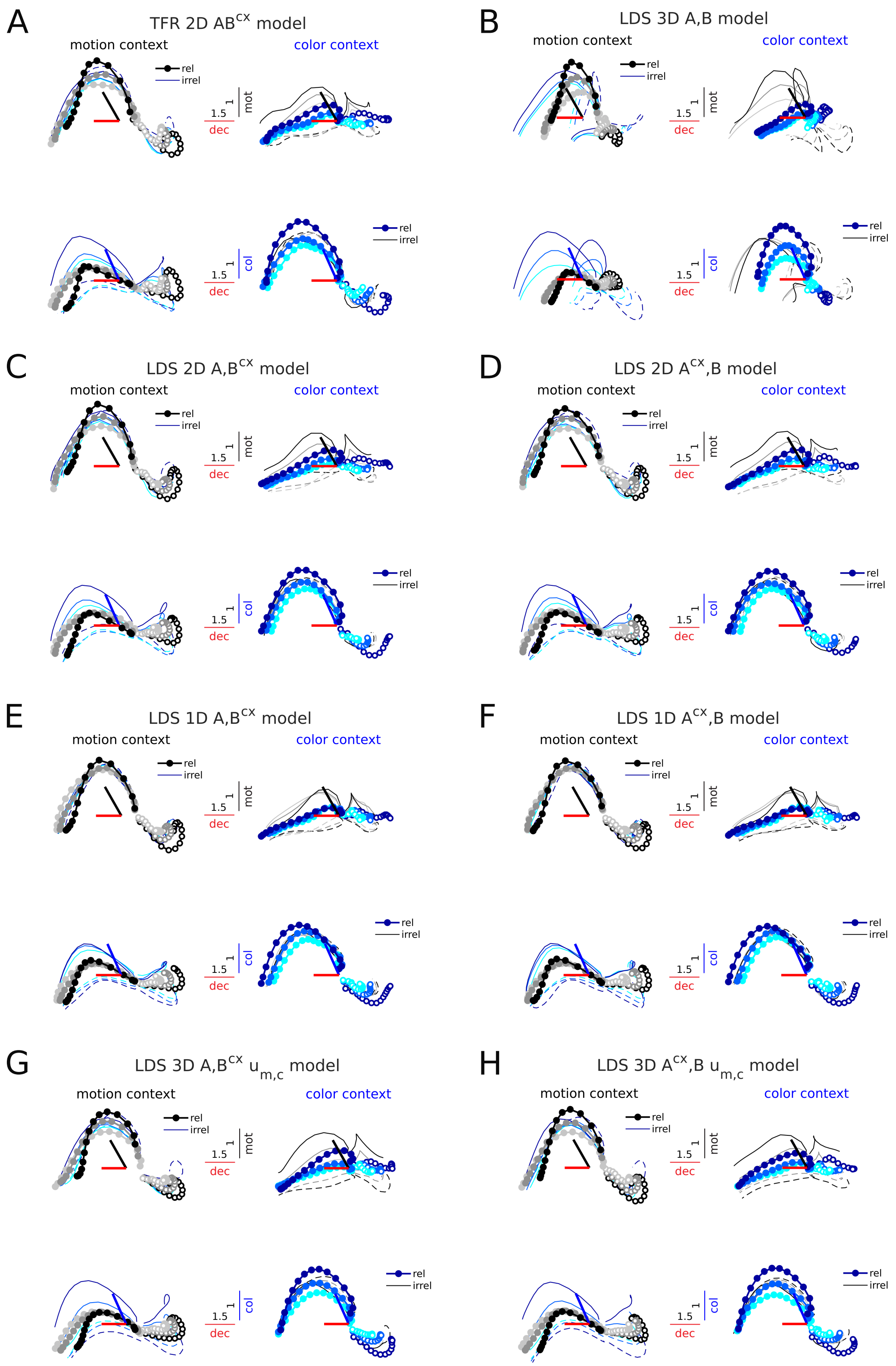

A
TFR 2D AB^cx model
motion context
color context
rel
irrel
mot
col
1
1.5
dec
B
LDS 3D A,B model
C
LDS 2D A,B^cx model
D
LDS 2D A^cx,B model
E
LDS 1D A,B^cx model
F
LDS 1D A^cx,B model
G
LDS 3D A,B^cx u_m,c model
H
LDS 3D A^cx,B u_m,c model

**Fig. S2. Population trajectories of the TFR model and alternative LDS models in the task-related subspace.** Cross-validated model trajectories (LOOCV) for the best TFR model and additional LDS models with various input dimensionalities and input/contextual constraints. (**A**) TFR $\{AB^{cx}\}$ model with 2D inputs. (**B**) LDS $\{A, B\}$ model with 3D inputs. This model poorly captures the trajectories, specially along the decision dimension. (**C,D**) LDS $\{A, B^{cx}\}$ and $\{A^{cx}, B\}$ models with 2D inputs. These capture the trajectories almost as well as the 3D models (Fig. 3C,D) (**E,F**) LDS $\{A, B^{cx}\}$ and $\{A^{cx}, B\}$ models with 1D inputs. Note that the trajectories along the input dimensions are not accurately captured, in particular for the irrelevant inputs, where trajectories poorly separate by coherence condition. (**G,H**) LDS $\{A, B^{cx}\mathbf{u}_{m,c}\}$ and $\{A^{cx}, B\mathbf{u}_{m,c}\}$ models with time-constant 3D inputs. These also perform nearly as well as the 3D LDS models with time-varying inputs (Fig. 3C,D, Fig. S5B). Same conventions as in Fig. 3. All trajectories have been smoothed with a Gaussian filter for visualization (sliding window size, 5-bins). This step did not change much the LDS trajectories, since they are inherently smooth, but it helped smooth-out substantially the TFR model trajectories, given that this model has no dynamical constraints that enforce smoothness. Monkey A data.

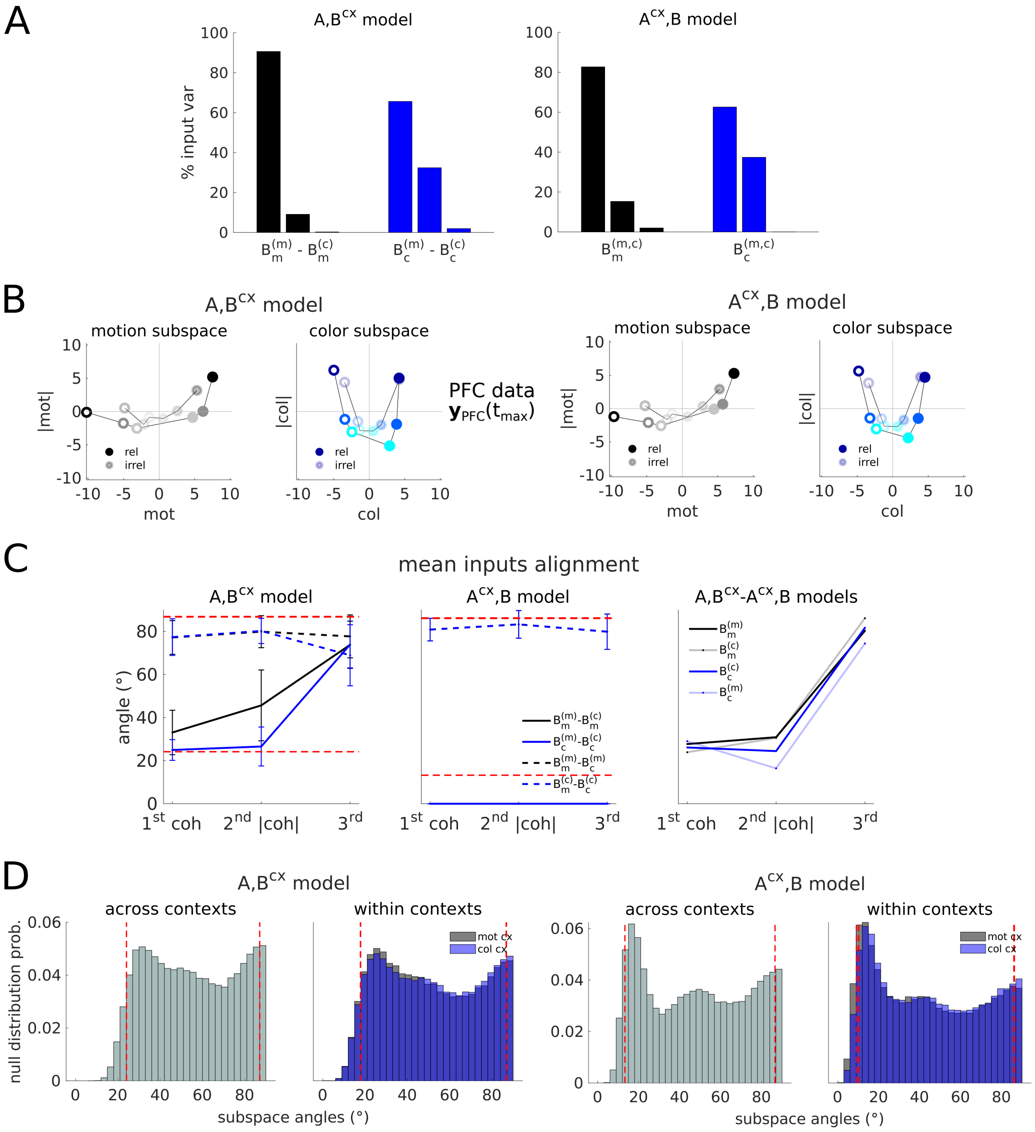

A
A,B^cx model
A^cx,B model
% input var
B_m^(m) - B_m^(c)
B_c^(m) - B_c^(c)
B_m^(m,c)
B_c^(m,c)
B
A,B^cx model
A^cx,B model
motion subspace
color subspace
|mot|
|col|
mot
col
rel
irrel
PFC data
y_PFC(t_max)
C
mean inputs alignment
A,B^cx model
A^cx,B model
A,B^cx-A^cx,B models
angle (°)
1st coh
2nd |coh|
3rd
B_m^(m)-B_m^(c)
B_c^(m)-B_c^(c)
B_m^(m)-B_c^(m)
B_m^(c)-B_c^(c)
B_m^(m)
B_m^(c)
B_c^(c)
B_c^(m)
D
A,B^cx model
A^cx,B model
across contexts
within contexts
null distribution prob.
subspace angles (°)
mot cx
col cx

**Fig. S3**. **Input variance, PFC data in the 2D input subspaces and alignment statistics.** (**A**) External inputs variance in the three orthonormalized input dimensions from both LDS models. For the $\{A, B^{cx}\}$ model variance is computed along the mean dimensions across contexts (first taking the average across contexts: mot avg. $B_m^{(m)} - B_m^{(c)}$, for dims 1-3, and col avg. $B_c^{(m)} - B_c^{(c)}$, for dims 1-3, then re-orthogonalizing the three input dimensions). Note that most of the input variance is concentrated in the first two input dimensions (the 2D plane capturing coherence an coherence magnitude variance, found via regression, and then orthonormalised using a QR factorization [1]) whereas the third dimension carry almost no input variance (see also Fig. S4C,F, top panels). Averages across 100 models. (**B**) PFC data at t=250ms for all coherences and both contexts, projected onto the same 2D coh-|coh| input planes as in Fig. 4D,E. The PFC data contains a curved representation of coherence information. CI signals have been subtracted. (**C**) Alignment between the motion and color input vectors within contexts (dashed lines), and between the motion or color input vectors across contexts (filled lines), for each of the three input dimensions, and the two LDS models (left and middle panels). Dashed red lines, 5th and 95th percentiles of a null distribution of alignments (see (**D**)). Error bars, std across a 100 randomly initialized models. Note that the inferred coherence and coherence magnitude dimensions for both motion and color are largely stable across contexts in the $\{A, B^{cx}\}$ model (i.e. they are highly aligned, filled lines). However, the across-contexts alignments for the third input dimension are close to orthogonal, indicating that this dimension is not common across contexts. Right panel, alignments between the mean input directions (across 100 models) from each LDS model class. The coh and —coh— directions are highly consistent across models. (**D**) Null distribution of alignments (subspace angles) from randomly sampled 3D subspaces within and across contexts drawn aligned to the data covariance [74], orthonormalized, and then projected onto the low-d subspaces from each LDS model class (defined by the columns of the loading matrices $C$). The null distributions from the two model classes are different since they learned different $C$ matrices. Random samples s≈33,000 orthonormal subspaces, or 100,000 vectors. Dashed red lines, 5th and 95th null distribution percentiles. Alignments not expected by chance fall in regions $\leq$ the 5th or $\geq$ the 95th percentiles of the control distributions. Additionally, given the binomial nature of the distribution, where both high and low alignments are expected, random alignments should on average lie around $50°$ and have large variance. This is not what is typically obtained from the data (panel (**C**)). Furthermore, in all 100 models from the $\{A, B^{cx}\}$ class, the highest alignments consistently occurred between the two color and two motion dimensions across contexts, and not between the motion and color dimensions within contexts (first panel in (**C**), filled vs. dashed lines). This was true only for the first two input dimensions, but not the third, where all alignments are very low. Similarly, the first two mean input dimensions were highly aligned across model classes, but not the third ((**C**), third panel). Monkey A data.

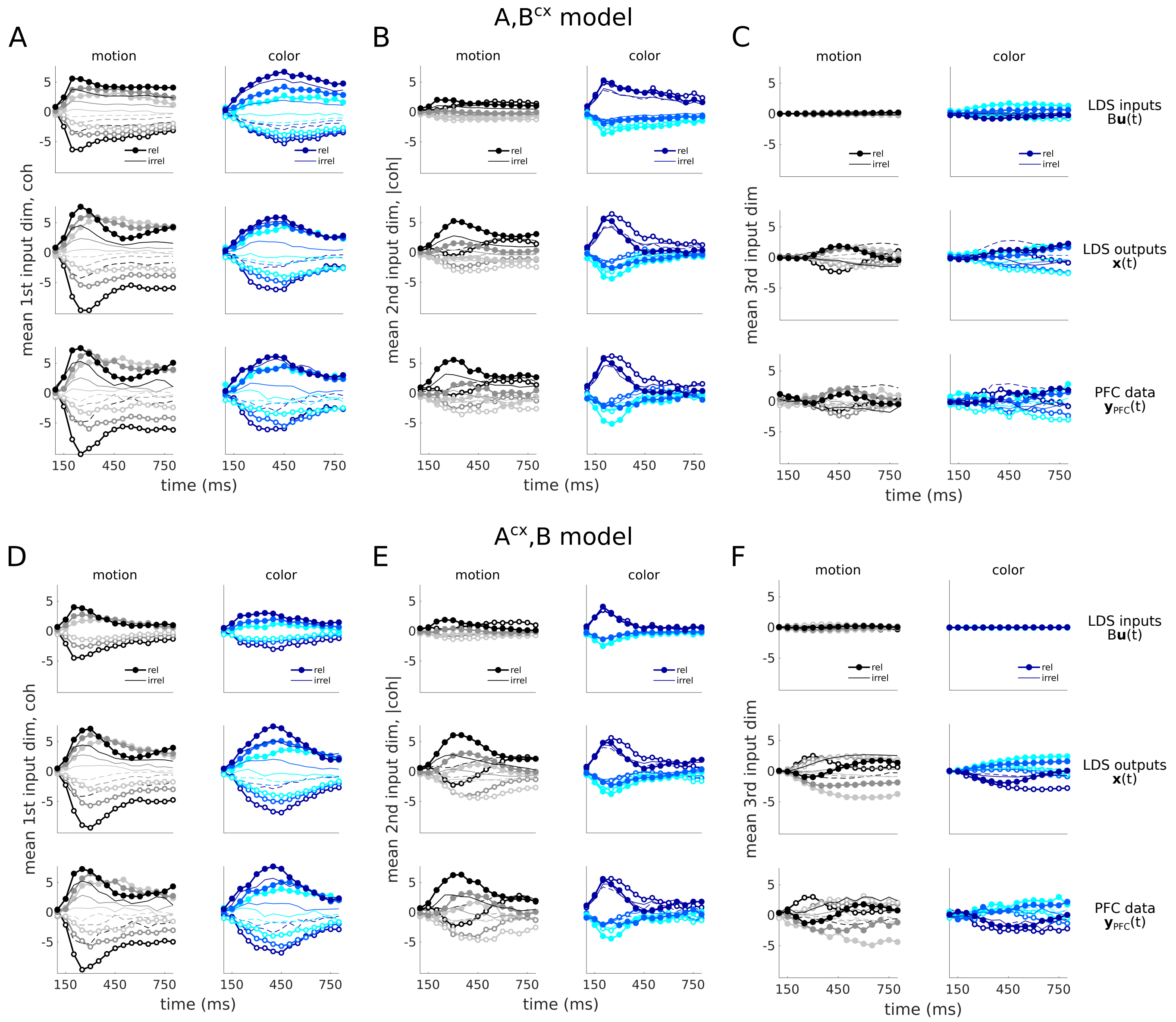
A,B^cx model
A
B
C
motion
color
rel
irrel
mean 1st input dim, coh
mean 2nd input dim, |coh|
mean 3rd input dim
LDS inputs
Bu(t)
LDS outputs
x(t)
PFC data
y_PFC(t)
time (ms)
A^cx,B model
D
E
F

**Fig. S4**. **LDS models external inputs, latents and PFC data in the LDS input dimensions.** LDS external inputs, LDS cross-validated latents (outputs) and PFC data trajectories projected along the three input dimensions found in the $\{A, B^{cx}\}$ ((**A-C**)) and $\{A^{cx}, B\}$ ((**D-F**)) models, for all coherence conditions and contexts (relevant vs. irrelevant). (**A,D**) First input dimension, capturing coherence related variance (coh). (**B,E**) Second input dimension, capturing coherence magnitude related variance (|coh|). (**C,F**) Third input dimension, orthogonal to the coh and |coh| dimensions, capturing little input and relatively little output/data variance compared to the other dimensions (in particular, for color). For the $\{A, B^{cx}\}$ model, projections are shown onto the direction bisecting the two color and two motion directions found for each context. All data is from means over 100 models initialized at random. The three mean color and motion input dimensions are orthogonalized with QR-factorization. For all trajectories the mean across conditions has been subtracted out to remove condition independent signals (CI). Latents and data trajectories are generated for all 36 task conditions and plotted along the motion/color input dimensions with color/motion conditions averaged out, as in [1]. Same plotting conventions as in Fig. 3. Monkey A data.

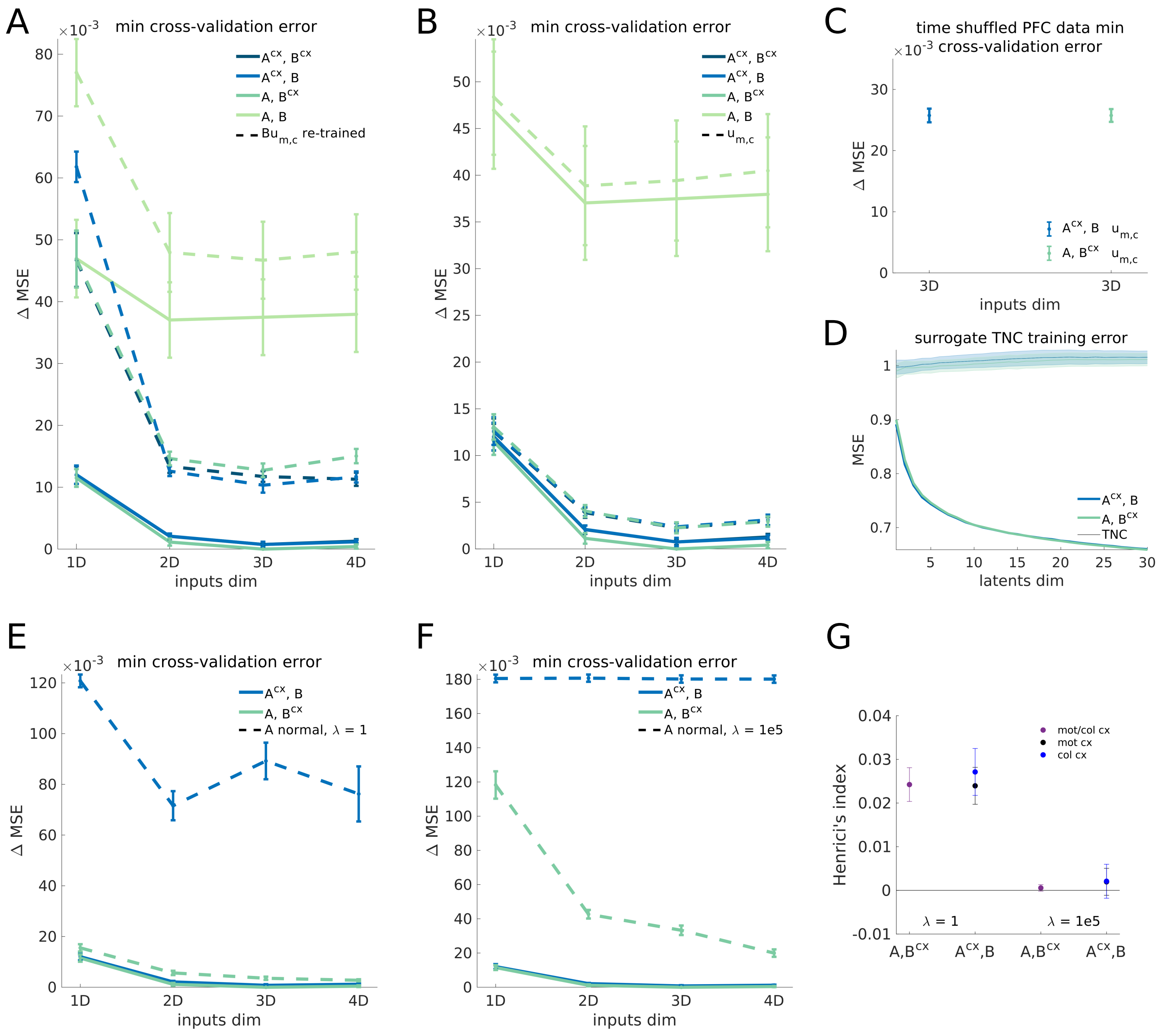

A
min cross-validation error
×10^-3
Δ MSE
$A^{cx}$, $B^{cx}$
$A^{cx}$, B
A, $B^{cx}$
A, B
$Bu_{m,c}$ re-trained
1D
2D
3D
4D
inputs dim
B
min cross-validation error
×10^-3
Δ MSE
$A^{cx}$, $B^{cx}$
$A^{cx}$, B
A, $B^{cx}$
A, B
$u_{m,c}$
inputs dim
C
time shuffled PFC data min cross-validation error
×10^-3
Δ MSE
$A^{cx}$, B $u_{m,c}$
A, $B^{cx}$ $u_{m,c}$
3D
3D
inputs dim
D
surrogate TNC training error
MSE
$A^{cx}$, B
A, $B^{cx}$
TNC
latents dim
E
min cross-validation error
×10^-3
Δ MSE
$A^{cx}$, B
A, $B^{cx}$
A normal, λ = 1
inputs dim
F
min cross-validation error
×10^-3
Δ MSE
$A^{cx}$, B
A, $B^{cx}$
A normal, λ = 1e5
inputs dim
G
Henrici's index
mot/col cx
mot cx
col cx
λ = 1
λ = 1e5
A,$B^{cx}$
$A^{cx}$,B
A,$B^{cx}$
$A^{cx}$,B

**Fig. S5**. **Performance of LDS models with time-constant inputs, randomized data controls and normality constraints.** (**A**) Leave-one-condition-out cross-validation performance (LOOCV) of the LDS models with time-varying inputs in Fig. 2A (filled lines) and the same models after re-training their input parameters $B\mathbf{u}_{m,c}$, but constraining $\mathbf{u}_{m,c}$ to be constant in time, and with the rest of the parameters kept the same (dashed lines). See next panel for performance of a similar model but fully optimized (all parameters re-trained). (**B**) Performance of LDS models with $\mathbf{u}_{m,c}$ constant in time where all parameters, including the dynamics matrix, are optimized to fit the data (dashed lines). The time-varying input models from Fig. 2A are also shown for reference (filled lines). (**C**) Performance of the best time-constant models ($\{A, B^{cx}\mathbf{u}_{m,c}\}$ and $\{A^{cx}, B\mathbf{u}_{m,c}\}$ 3D models, dashed lines in (**B**)) when fitted to time-shuffled PFC data. Note that the performance drops substantially (Δ MSE = 26), being worse than the 1D input models and nearly as bad as the most contextually constrained $\{A, B\}$ models (see (**B**)). For all three subpanels ((**A**)-(**C**)) the minimum cross-validation errors are shown relative to the best performing LDS model (the time-varying $\{A, B^{cx}\}$ model with 3D inputs). Error bars indicate the standard error mean across LOOCV folds. (**D**) Training performance of the best time-varying LDS models on surrogate data sets randomized across time, neurons and conditions (TNC), but designed to preserve the primary statistics of the data [13]. The LDS models perform poorly on these type of data. To obtain the randomized TNC data sets the tensor maximum entropy method (TME) was used. Shades indicate standard error mean across 30 surrogates. (**E**) LOOCV performance of the LDS $\{A, B^{cx}\}$ and $\{A^{cx}, B\}$ models (as in Fig. 2A, filled lines), and the same models but trained with normal dynamics constraints (dashed lines), for a penalty weight of $\lambda_{dyn} = 1$ (**F**) Same as in (**E**) but for a stronger normality penalty weight, $\lambda_{dyn} = 1e5$ (**G**) Dynamics non-normality index for both LDS models under the different normality penalty weights. Error bars, std across 100 models. Monkey A data.

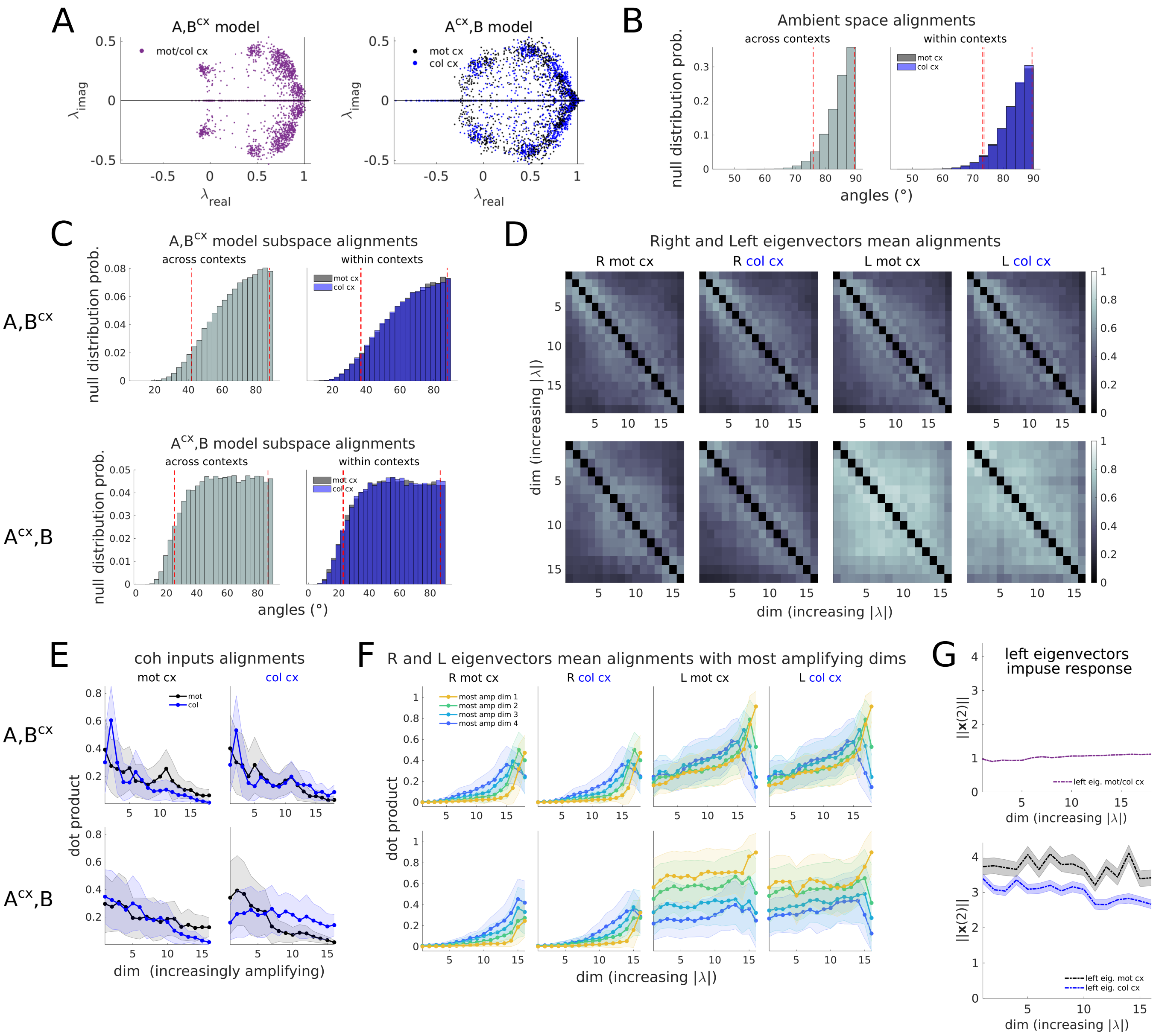

A
A,B^cx model
mot/col cx
A^cx,B model
mot cx
col cx
λimag
λreal
B
Ambient space alignments
across contexts
within contexts
null distribution prob.
angles (°)
C
A,B^cx model subspace alignments
A^cx,B model subspace alignments
A,B^cx
A^cx,B
D
Right and Left eigenvectors mean alignments
R mot cx
R col cx
L mot cx
L col cx
dim (increasing |λ|)
E
coh inputs alignments
dot product
dim (increasingly amplifying)
F
R and L eigenvectors mean alignments with most amplifying dims
most amp dim 1
most amp dim 2
most amp dim 3
most amp dim 4
G
left eigenvectors impuse response
left eig. mot/col cx
left eig. mot cx
left eig. col cx
||x(2)||

**Fig. S6**. **LDS models dynamics properties.** (**A**) LDS eigenspectrums for the 100 randomnly initialized models from each model class. (**B,C**) Null distribution of alignments for randomly sampled pairs of vectors, within contexts or across contexts, drawn aligned to the data covariance [74] in the ambient (high-d) space ((**B**)) or projected onto the low-d LDS subspaces from each model class ((**C**), defined by the columns of the loading matrices $C$). Random samples s=100,000 vectors. Dashed red lines, 5th and 95th null distribution percentiles. Alignments not expected by chance fall in regions $<$ the 5th or $>$ the 95th percentiles of the control distributions. (**D**) Mean alignments among the left/right eigenvectors from each model class. $\{A, B^{cx}\}$, top, $\{A^{cx}, B\}$, bottom, averages across 100 models from each class. Note that the left eigenvectors in the $\{A^{cx}, B\}$ model are highly aligned, which explains its strong non-normal dynamics (Fig. 6C). (**E**) Motion and color input coherence vector alignments with respect to dynamic dimensions of various degrees of amplification, sorted from the least to the most amplifying modes (Methods), for the two contexts (left and right panels) and the two LDS model classes (top, bottom). Mean $\pm$ std across 100 models. Motion and color inputs do not strongly align with the most amplifying dimensions. If anything, they preferentially align with the least amplifying dimensions. Note that in Fig. 5C we found that coherence inputs were strongly loaded onto the relatively fast decaying left eigenmodes. Indeed, these intermediate left eigenmodes do not align particularly strongly to the most amplifying modes, compared to the alignments for other eigenmodes (see (**F**), L panels). (**F**) Right (R) and Left (L) eigenvectors alignments with respect to the four most amplifying modes of the dynamics (Methods), for both models and both contexts. Mean $\pm$ std across 100 models. Note that all left eigenvector dimensions, from fast, to intermediate, to slow, have moderate to strong alignments with the most amplifying modes. In fact, all left eigenvector directions amplify inputs similarly (see next panel). Interestingly, for the right eigenvectors, only the slowest directions align to the most amplifying dimensions (albeit moderately). (**G**) Impulse response along each left eigenvector direction, measured at the time right after the unit norm perturbation (t=2). The state at this time indicates the degree of transient amplification immediately after the pulse (see also Fig. 6A for response over time, averaged across all left eigenvectors). The state norm at t=2 is slightly bigger than 1 for all $\{A, B^{cx}\}$ left eigenvectors, indicating that all these directions slightly amplify inputs. For the $\{A^{cx}, B\}$, in both contexts, the impulse response is much bigger than one for all left eigenvectors, indicating that all these directions strongly amplify inputs. This confirms that the dimensions where the inputs are mostly loaded, the intermediate or relatively fast decaying dimensions, are not particularly amplifying, relative to the fastest and slowest dimensions. Monkey A data.

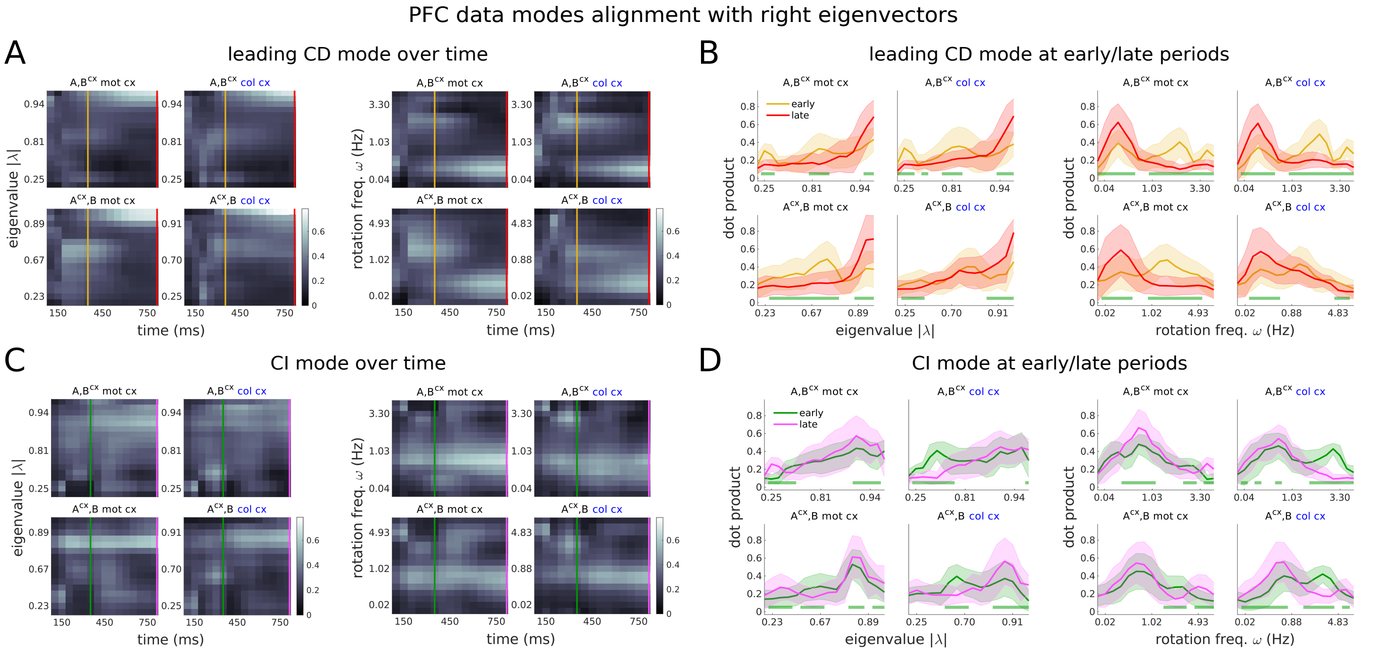


**Fig. S7. PFC integration phases for both LDS models across contexts.** Same as in Fig. 7 but for both models and contexts. (**A,B**) The two distinct integration phases are consistently observed across model classes ($\{A, B^{cx}\}$ and $\{A^{cx}, B\}$), contexts (mot and col cx) and model instantiations ((**B**), the distribution of alignments across 100 randomly initialized models are fairly narrow, mean $\pm$ std). Note that for the $\{A^{cx}, B\}$ model in the color context, the early and late alignment distributions are not significantly different along the intermediate set of eigenmodes (at significance level p<0.001, (**B**)). However, the early distribution clearly peaks around the intermediate modes, which have relatively fast decaying dynamics ((**B**), left) and fast rotations ((**B**), right). The early and late alignment distributions are always significantly different along the largest modes, which have the slowest dynamics ((**B**), left) and very small rotation frequencies ((**B**), right). (**C,D**) CI signals are integrated along a different set of slow modes than CD signals, consistently across models and contexts (the peaks of the CI distributions, (**D**), lie in different ranges of slow eigenvalues than the early and late CD distribution peaks, (**B**)). The integration of CI signals do not clearly separate in two phases given that the alignments are largely steady across the trial ((**C**)). Indeed, the distribution of alignments early vs. late largely overlap ((**D**), green and magenta distributions). Monkey A data.

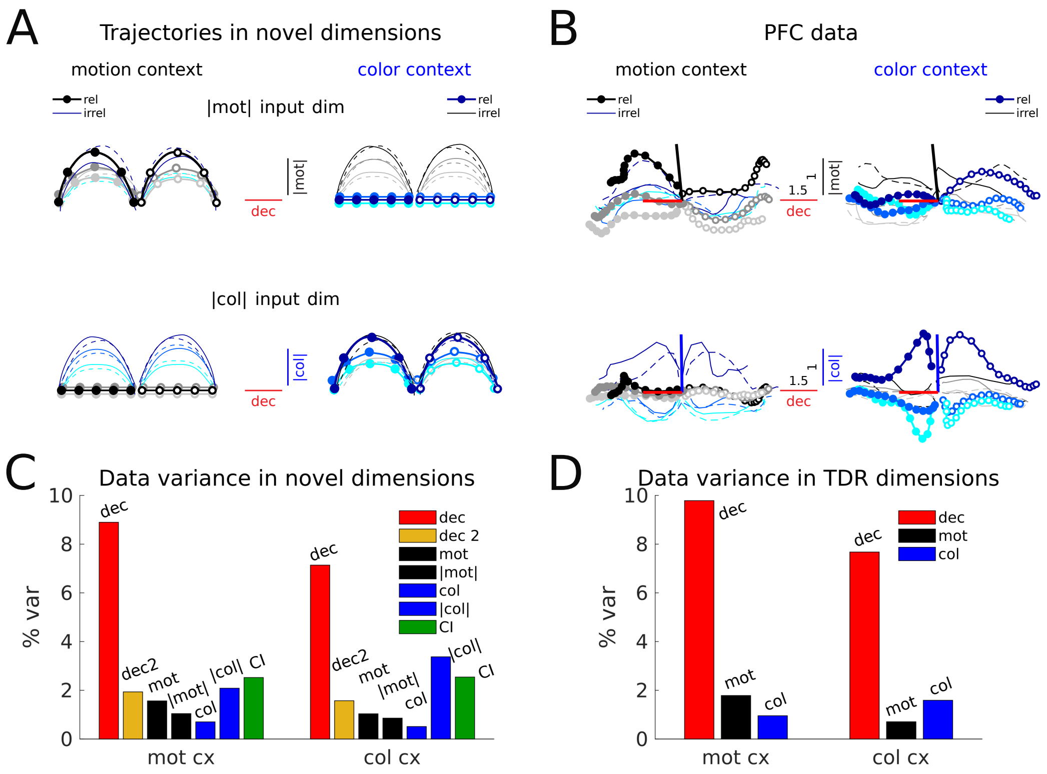


**Fig. S8**. **PFC data trajectories in coherence magnitude dimensions and data variance in the novel and TDR dimensions.** (**A,B**) Same as in Fig. 8, but for the LDS-inferred coherence magnitude dimensions (averaged across contexts and models). The condition independent (CI) variance has been subtracted-out from the trajectories to emphasise input-related variance. (**C**) PFC data variance in the novel decision, secondary decision, motion coherence, motion coherence magnitude, color coherence, color coherence magnitude and condition independent (CI) dimensions. Note that the variance that is reflected in each dimension is the total variance, and not the isolated task-related variance. For instance, there is substantial CI variance in the input dimensions, in particular the color coherence magnitude ones, which we removed in panel (**B**) to emphasize input-related features. (**D**) Variance in TDR decision, motion and color dimensions. Note that this is the variance of the raw data, and not the variance of de-noised data as reported in [1], which was computed on the data projected onto its leading PCs. Monkey A data.

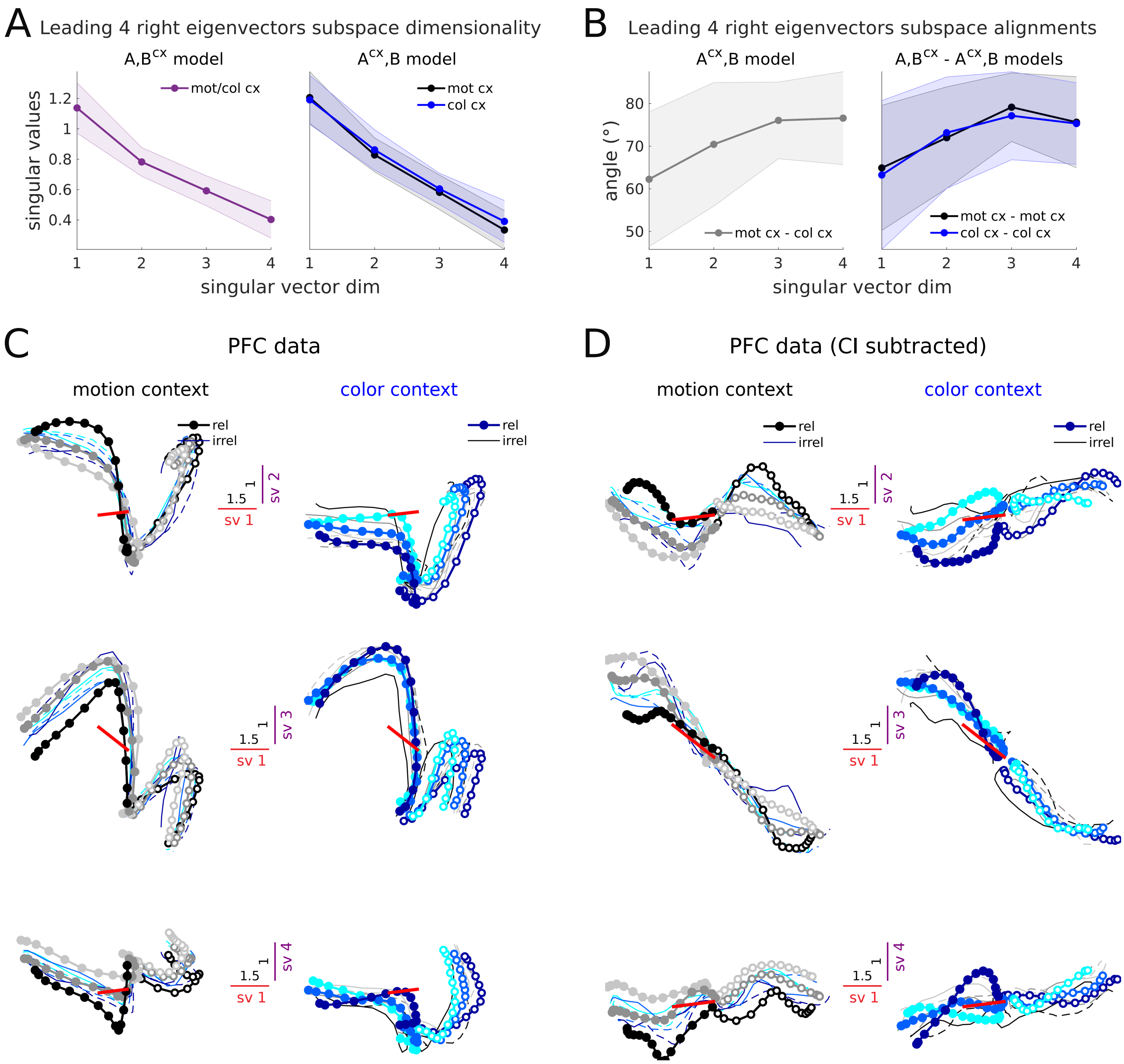

A
Leading 4 right eigenvectors subspace dimensionality
A,B$^{cx}$ model
mot/col cx
$A^{cx}$,B model
mot cx
col cx
singular values
singular vector dim
B
Leading 4 right eigenvectors subspace alignments
$A^{cx}$,B model
mot cx - col cx
A,B$^{cx}$ - $A^{cx}$,B models
mot cx - mot cx
col cx - col cx
angle (°)
singular vector dim
C
PFC data
motion context
color context
rel
irrel
sv 1
sv 2
sv 3
sv 4
D
PFC data (CI subtracted)
motion context
color context
rel
irrel

**Fig. S9. Choice signals in the slowest LDS subspace largely evolve along a single dimension across contexts.** (**A**) The four slowest dimensions inferred by the LDS models have very long time constants ($|\lambda| > 0.9$, time-constant $\tau > 475$ms, average across 100 models, Fig. 5B), so they are expected to define a 4D subspace that contains highly persistent signals. However, the associated right eigenvectors are not orthogonal (Fig. S6D), so these signals could occupy fewer than four dimensions. We measured the effective dimensionality of the subspace spanned by the four slowest right eigenvectors by taking the SVD of the matrix containing them. We found that the right eigenvectors effectively span four dimensions, since all singular values are well above zero. Mean $\pm$ std across 100 models. The dominant dimension (1st singular vector) is also the one most highly aligned across contexts in the $\{A^{cx}, B\}$ model and also across models (see next panel). (**B**) Alignments of the slowest subspace dimensions found across contexts in the $\{A^{cx}, B\}$ model (left panel) and across models (right panel). Mean $\pm$ std across 100 models. The alignments are moderate to weak and expected by chance from a control distribution of random vector alignments in the low-d LDS subspaces (alignments are within the 5th and the 95th percentiles of the control distributions, Fig. S6C). However, the largest alignments occur precisely for the first dimension. The moderate alignments may be explained by the effect of CI signals across contexts (see next panel) (**C**) PFC data projected in the four singular vector (sv) dimensions of the slowest subspace (averaged across models and contexts, and then re-orthonormalized). Importantly, the first dimension captures decision information, but dimensions 2 to 4 mostly capture condition independent (CI) variance, and some contextual variance (in particular sv2, where the trajectories from each context are slightly shifted vertically). (**D**) Same as (**C**) but after subtracting CI signals. Red bars show the alignment of the decision dimension found by TDR with respect to the four averaged singular vector dimensions (sv1: $36°$, sv2: $86°$, sv3: $65°$, sv4: $86°$). The first singular vector dimension highly aligns with the decision dimension ($36°$), and the third one moderately aligns to it ($65°$). These alignments are not expected by chance, considering a control distribution of random alignments in the ambient (high-d) space (alignments $<$ 5th percentiles of the control distributions, Fig. S6B). The 2D subspace spanned by these two singular vector dimensions (middle panels) contains trajectories mainly evolving along a single dimension, when CI signals are subtracted out (compare to middle panels in (**C**)). This dimension is common across contexts and strongly aligns to the decision axis found by Mante et al. (red bars). Similar results are obtained when projecting the data into the sv dimensions computed for each context and model independently. Monkey A data.

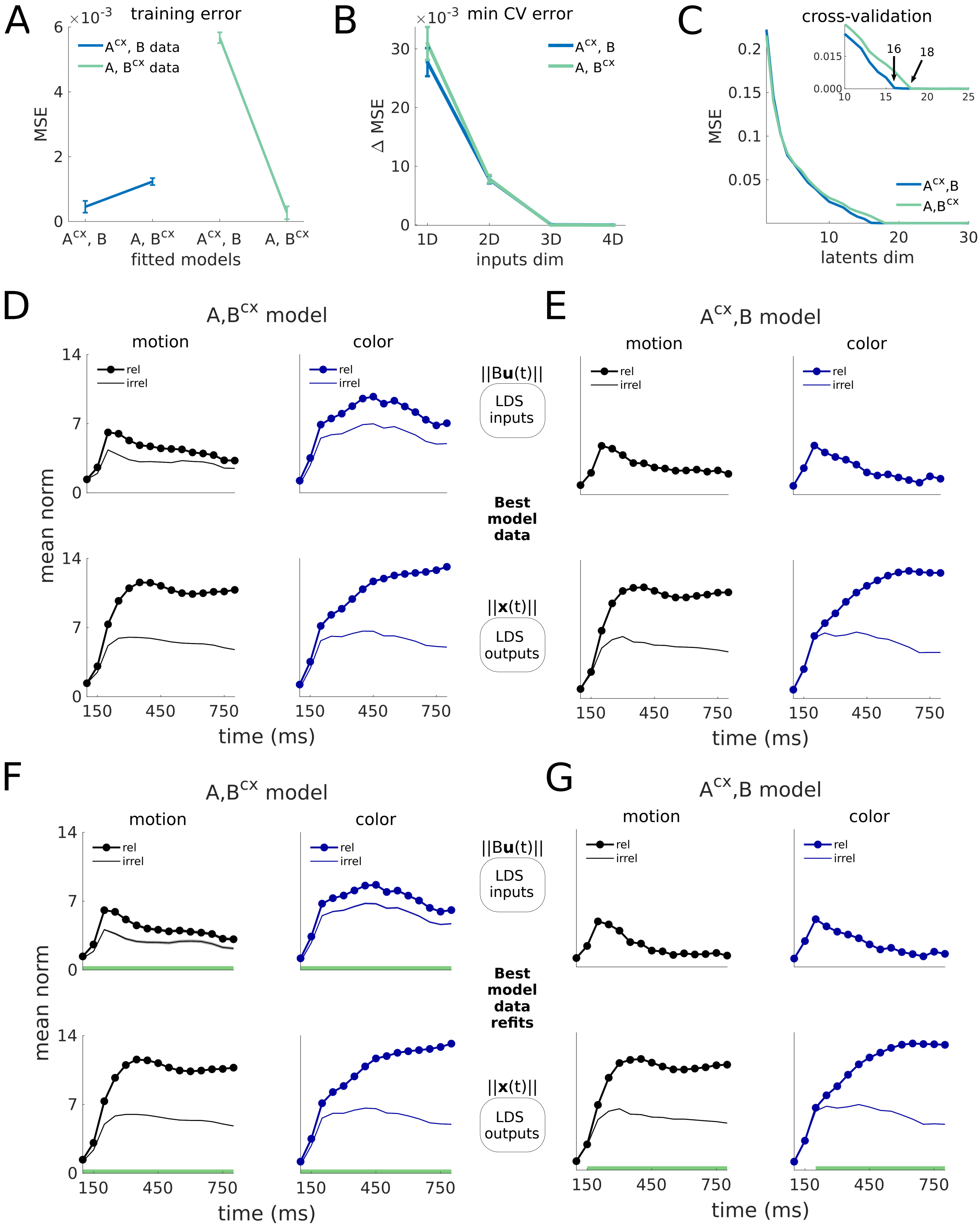

A
training error
MSE
$\times 10^{-3}$
$A^{cx}$, B data
A, $B^{cx}$ data
fitted models
B
min CV error
Δ MSE
inputs dim
C
cross-validation
16
18
latents dim
D
A,$B^{cx}$ model
motion
color
rel
irrel
||Bu(t)||
LDS inputs
Best model data
||x(t)||
LDS outputs
mean norm
time (ms)
E
$A^{cx}$,B model
F
A,$B^{cx}$ model
Best model data refits
G
$A^{cx}$,B model

**Fig. S10**. **LDS models: parameter recovery.** (**A**) Mean training performance of models re-fitted to model-generated data (N=100). The re-fitted models are either from the same class as the data or the wrong class. Model-generated data is from the best performing $\{A^{cx}, B\}$ and $\{A, B^{cx}\}$ models that were fitted to the PFC data (out of 100 models). Error bars, std. (**B**) Leave-one-condition-out cross-validation performance (LOOCV) for re-fitted LDS models of the same model class as the model-generated data. Shown are minimum cross-validation errors across different latent dimensionalities relative to the best performing LDS model ($\{A, B^{cx}\}$ model). Error bars indicate the standard error mean across LOOCV folds. The LOOCV saturates at input dim = 3, the true input dimensionality of the model-generated data. (**C**) Same as in (**B**) but showing the latent dimensionality for which the minimum LOOCV error is achieved (here, input dim = 3). The error saturates at xdim = 16 for the re-fitted $\{A^{cx}, B\}$ models and at 18 for the $\{A, B^{cx}\}$ models, which are the true latent dimensionalities of the model-generated data. (**D,E**) LDS models inputs and outputs. As in Fig. 4B,C but for the best performing model from each class. (**F,G**) Mean LDS models inputs and outputs from 100 models re-fitted to the data from the best model from each class (shown in (**D,E**)). Shades = sem. Monkey A data.

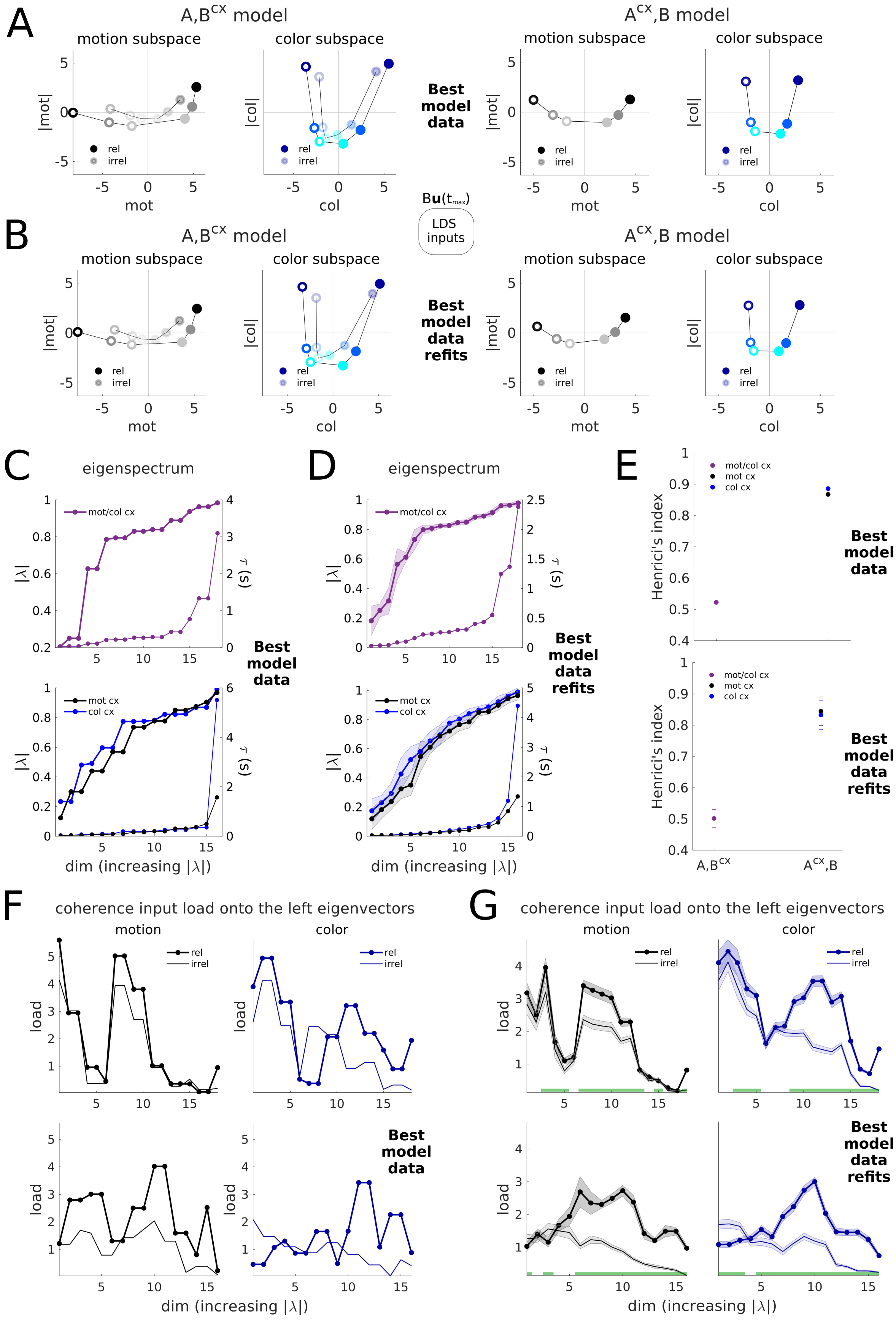

A
A,B^cx model
motion subspace
color subspace
Best model data
A^cx,B model
rel
irrel
mot
col
|mot|
|col|
Bu(t_max)
LDS inputs
B
Best model data refits
C
eigenspectrum
mot/col cx
mot cx
col cx
|λ|
τ (s)
dim (increasing |λ|)
D
E
Henrici's index
A,B^cx
A^cx,B
F
coherence input load onto the left eigenvectors
motion
color
load
G

**Fig. S11**. **LDS models: parameter recovery.** (**A**) LDS model inputs in coh-|coh| 2D planes (as in Fig. 4D,E) for the best models from each class. $\{A, B^{cx}\}$ model input planes alignments across contexts: $25°$ mot, $31°$ |mot|, $22°$ col, $26°$ |col| dims. (**B**) Mean inputs from re-fitted models of the same model class. $\{A, B^{cx}\}$ model input planes alignments: $28° \pm 4$ mot, $34° \pm 6$ |mot|, $24° \pm 3$ col, $26° \pm 4$ |col| dims, mean $\pm$ std. (**C**) Best models eigenspectrum (as in Fig. 5B). (**D**) Re-fitted models mean eigenspectrum. Shades=std. (**E**) Best models and re-fitted models non-normality index (as in Fig. 6C). (**F-G**) Best models and re-fitted models input loads (as in Fig. 5C). Re-fitted models, N=100. Monkey A data.

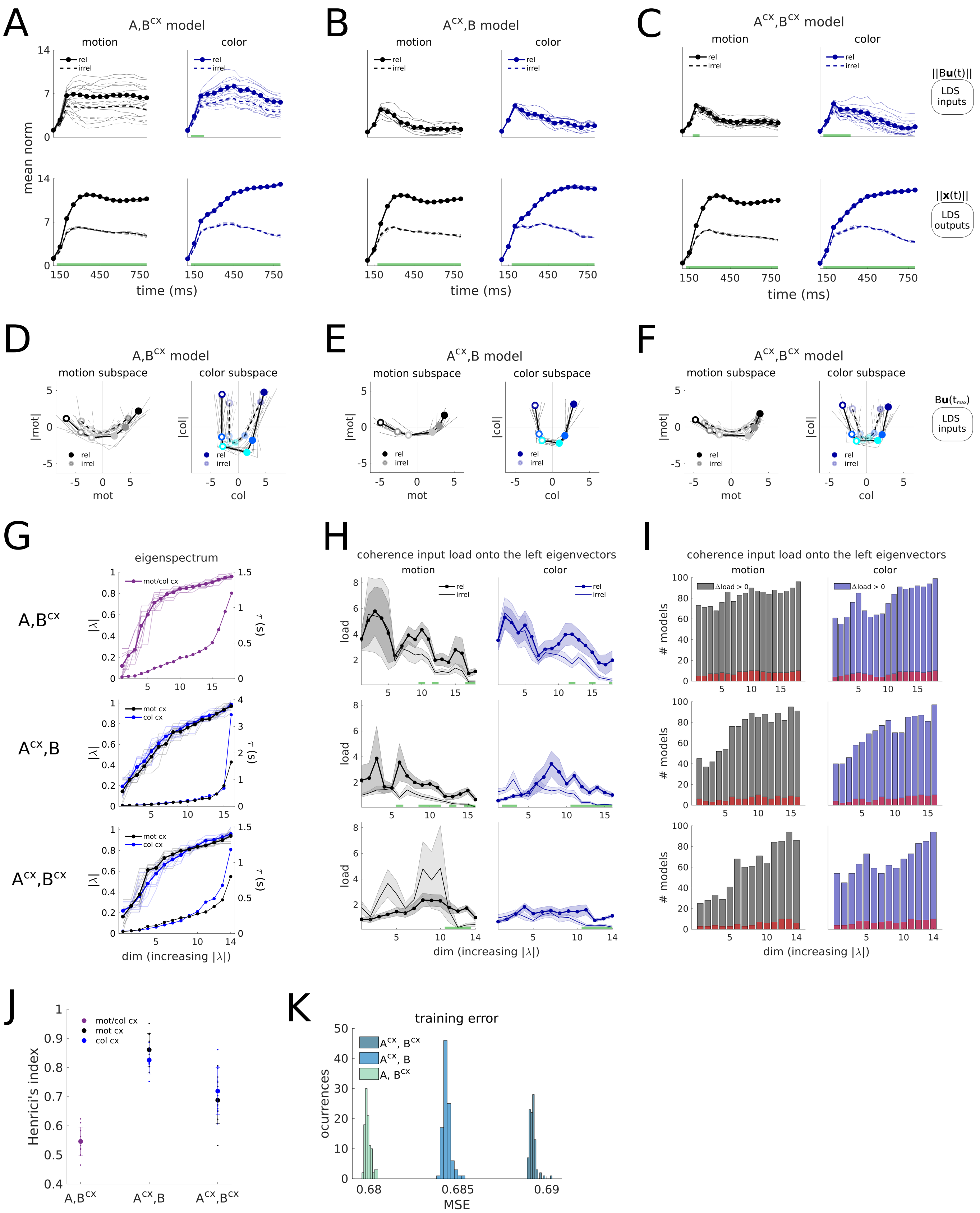

A
A,B$^{cx}$ model
motion
color
B
A$^{cx}$,B model
C
A$^{cx}$,B$^{cx}$ model
rel
irrel
mean norm
time (ms)
||Bu(t)||
LDS inputs
||x(t)||
LDS outputs
D
E
F
motion subspace
color subspace
|mot|
|col|
mot
col
Bu(t$_{max}$)
G
eigenspectrum
A,B$^{cx}$
A$^{cx}$,B
A$^{cx}$,B$^{cx}$
mot/col cx
mot cx
col cx
|λ|
τ (s)
dim (increasing |λ|)
H
coherence input load onto the left eigenvectors
load
I
# models
Δload > 0
J
Henrici's index
K
training error
ocurrences
MSE
A$^{cx}$, B$^{cx}$
A$^{cx}$, B
A, B$^{cx}$

**Fig. S12**. **LDS models: Best 10 models solutions.** (**A,B**) $\{A, B^{cx}\}$ and $\{A^{cx}, B\}$ best models inputs and outputs. As in Fig. 4B,C but for the 10 best performing models fitted to the PFC data. Thick dotted lines, mean relevant inputs and outputs; Thick dashed lines, mean irrelevant inputs and outputs. Thin lines, inputs and outputs for the best 10 model instances. (**C**) Same but for the $\{A^{cx}, B^{cx}\}$ model. (**D,E**) $\{A, B^{cx}\}$ and and $\{A^{cx}, B\}$ best models inputs in the coh-|coh| 2D planes (as in Fig. 4D,E). Thick lines, mean. Thin lines, individual models. Filled lines, relevant inputs. Dashed lines, irrelevant. Input planes alignments across contexts for the $\{A, B^{cx}\}$ model: 26° $\pm$ 5 mot, 36° $\pm$ 10 |mot|, 24° $\pm$ 4 col, 25° $\pm$ 7 |col| dims, mean $\pm$ std, N=10 models. (**F**) Same but for the $\{A^{cx}, B^{cx}\}$ model. Input planes alignments across contexts: 26° $\pm$ 8 mot, 54° $\pm$ 12 |mot|, 29° $\pm$ 5 col, 25° $\pm$ 5 |col| dims, mean $\pm$ std, N=10 models. (**G**) Eigenspectrum. Thick dotted lines, mean; thin lines, individual models. N=10. (**H**) Average input loads for the 10 best models (mean $\pm$ std). (**I**) Number of models with $\Delta$ load $> 0$ (relevant-irrelevant inputs load) onto each dynamic mode. Black and blue bars, motion and color input loads across all 100 models. Red bars, across the best 10 models. The relevant inputs are more strongly loaded than the irrelevant inputs along many modes, not just the slowest mode. This is consistent across a large fraction of the models fitted, including the 10 best models. (**J**) Dynamics non-normality index for the 10 best models from each model class. (**K**) Training performance of the 100 models fitted from each model class. Monkey A data.

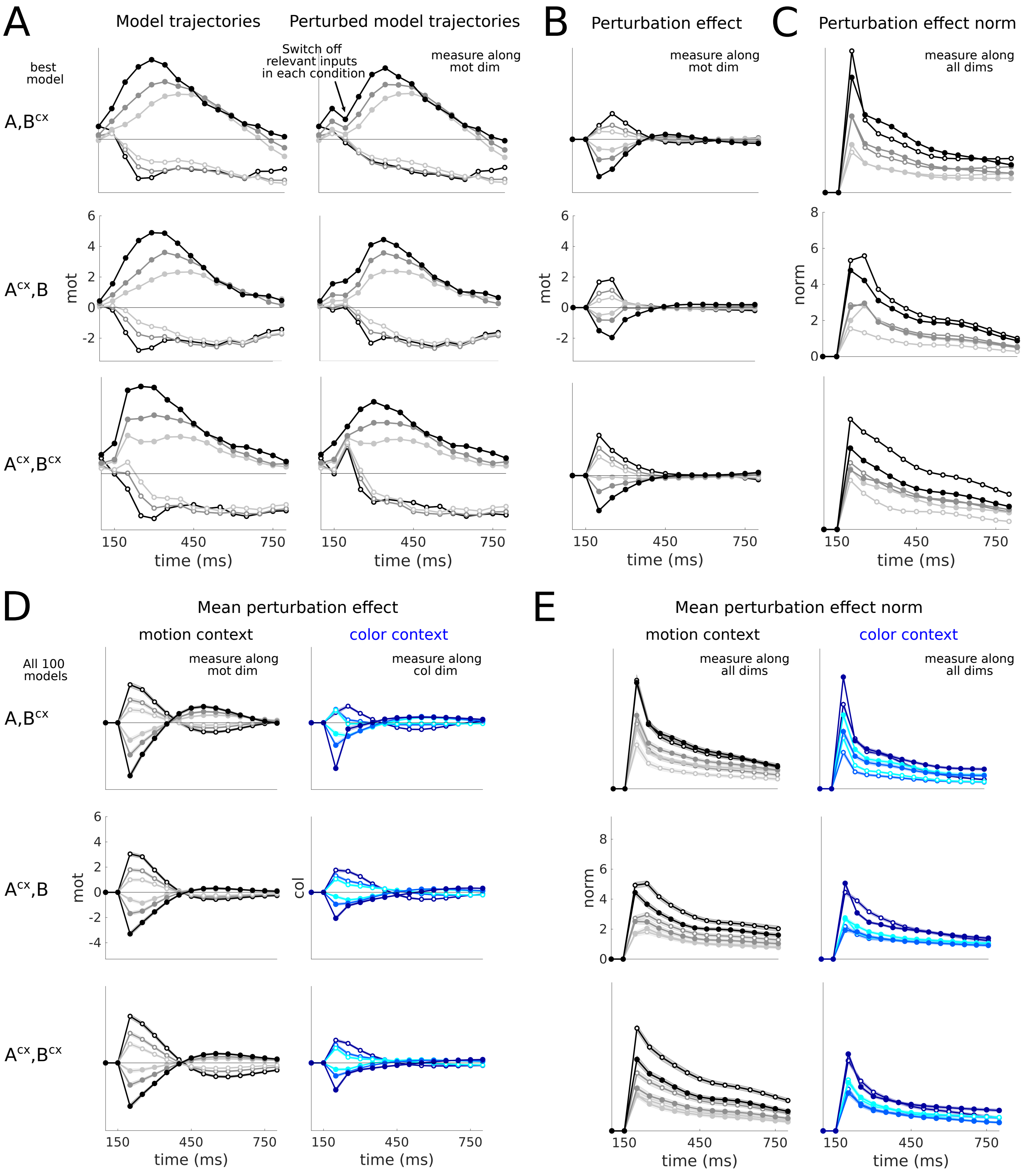
A
Model trajectories
Perturbed model trajectories
B
Perturbation effect
C
Perturbation effect norm
best model
Switch off relevant inputs in each condition
measure along mot dim
measure along mot dim
measure along all dims
A,B$^{cx}$
A$^{cx}$,B
A$^{cx}$,B$^{cx}$
mot
norm
time (ms)
D
Mean perturbation effect
motion context
color context
All 100 models
measure along mot dim
measure along col dim
mot
col
E
Mean perturbation effect norm
measure along all dims
norm

**Fig. S13**. **The LDS model classes predict different responses to input perturbations.** (**A**) Model generated trajectories (left panel) and model generated perturbed trajectories (right panel) projected along the motion dimension (as in Fig. 3B). Trajectories were generated for the motion context for all motion coherence conditions under the weakest positive color coherence (6 task conditions). An input perturbation was provided in the model by transiently ”switching off” the relevant inputs during each condition (by setting to zero the motion input in this case, for all 3 input dimensions) during the third time point of the trial (t=200ms after random dots onset). (**B**) Difference between the perturbed and unperturbed trajectories. In each of the 6 conditions a different perturbation was applied, since a different input was ”switched off” in each case. Because of this, the 6 revealed perturbations are distinct, and scale with the strength of the motion coherence. Best $\{A, B^{cx}\}$, $\{A^{cx}, B\}$ and $\{A^{cx}, B^{cx}\}$ models. (**C**) Norm of the perturbation effect measured within the entire observations space. The third time point reveals the strength of the perturbation. This is stronger for the $\{A, B^{cx}\}$ model. (**D**) Mean input perturbation effect across 100 fitted models. Input perturbations are applied to the motion and color inputs during the motion and color contexts, respectively (as in panel (**B**), but in the color context, the color inputs where switched off instead). Trajectories are colored by the relevant coherence condition; shades, 95% CI. The averages do not reveal large differences across model classes along the input dimensions. (**E**) Norm of the perturbation effect (as in (**C**)) averaged across 100 fitted models (plotting as in (**D**)). The average norm also does not reveal pronounced differences across the model classes, although the $\{A, B^{cx}\}$ model is more strongly impacted by input perturbations than the $\{A^{cx}, B\}$ model (see third time point). This is consistent across fitted models (see shades, 95% CI). Model $\{A^{cx}, B^{cx}\}$ lies in between. Monkey A data.

# Supplementary Figures 14–23

## Extended Analysis: Monkey F and RNN data.

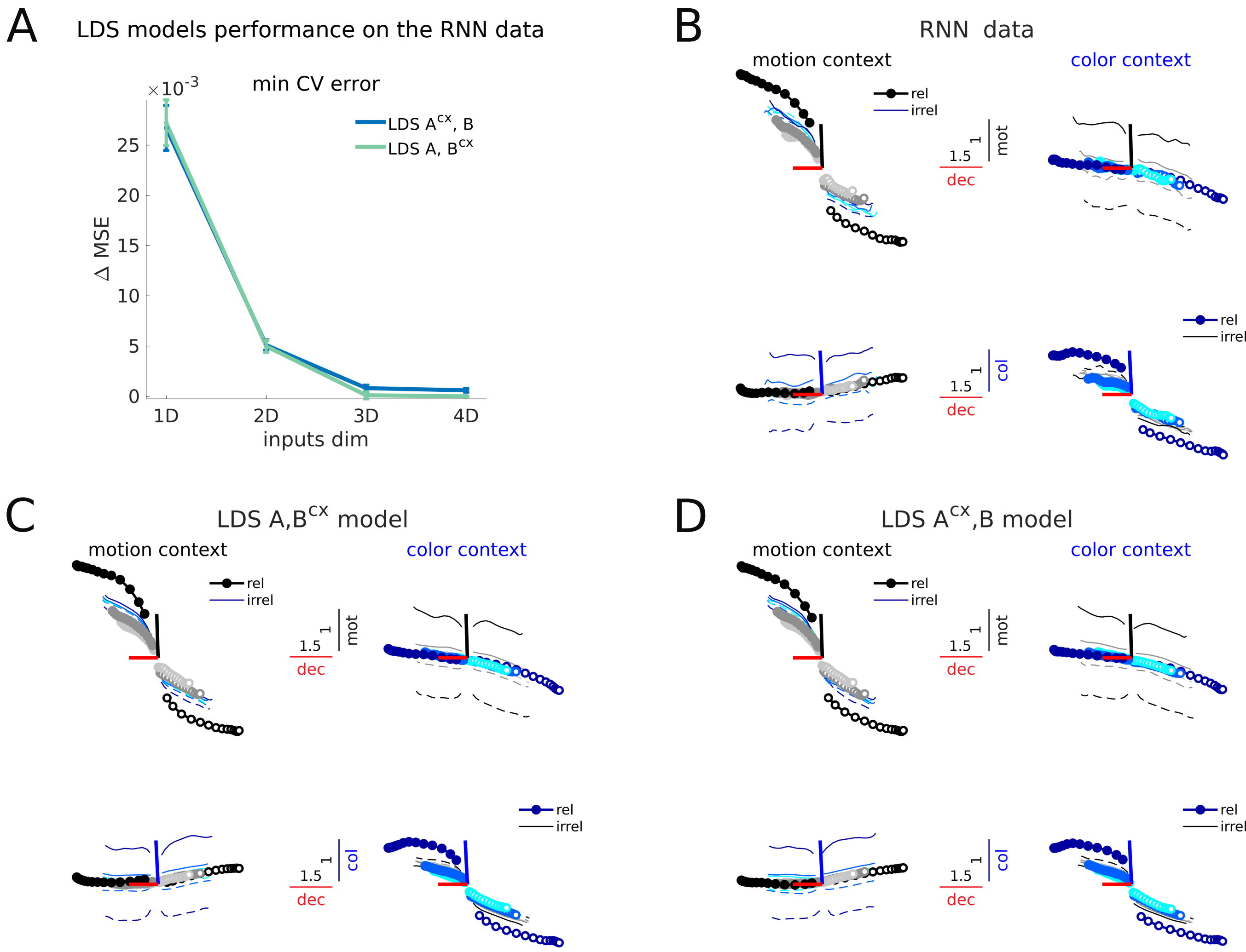

**Fig. S14**. **The LDS models accurately capture the RNN data.** (**A**) Same as Fig. 2A, but for the $\{A, B^{cx}\}$ and $\{A^{cx}, B\}$ models fitted to the RNN data. In this case the best performing model was the $\{A, B^{cx}\}$ model with 4D inputs. However, the performance of the $\{A, B^{cx}\}$ model with 3D inputs was not significantly different (Table S5). Therefore, in the RNN data the best performing models required at least 3D inputs, as for the PFC data (Fig. 2A). This was greater than the actual input dimensionality of the RNN model, which had 1D inputs (Fig. 1C, but see Fig. S15 for an explanation). The two LDS model classes performed equally well (Table S5), as in the PFC data (Fig. 2A), but the optimal latent dimensionality for the 3D $\{A, B^{cx}\}$ model was 26 and for the $\{A^{cx}, B\}$ model 14 (Table S5). The difference in dimensionality between the two model classes is larger than in the PFC data (18D vs. 16D, Table S1). This suggests that the $\{A, B^{cx}\}$ model struggles to capture the RNN data, since it needs more parameters. Note that the error for the two LDS models is close to zero (Table S5, LOOCV MSE≈0, or $\approx 0\%$ of variance missed), unlike for the PFC data from both monkeys (MSE≈0.73, or $\approx 73\%$ of variance missed, Supplementary Tables 1 and 3). Thus, the LDS models are able to approximate near perfectly the dynamics of the non-linear RNN in each context. This further validates the adequacy of our linear dynamics model-fitting approach. (**B-D**) Same as Fig. 3B-D but for the RNN data. Trajectories in the RNN task-related subspace are well captured by the both LDS models. Axes are defined by the RNN motion and color input vectors and the output vector (the decision readout) after training [1]. Note that the $\{A, B^{cx}\}$ model can capture well the input variance along the RNN input dimensions, which by design are fixed across contexts (Fig. 1C). In spite of this, this LDS model must have changed the inputs to achieve contextual integration (given that it can only change $B$), to accurately capture the substantial amount of variance along the decision axis.

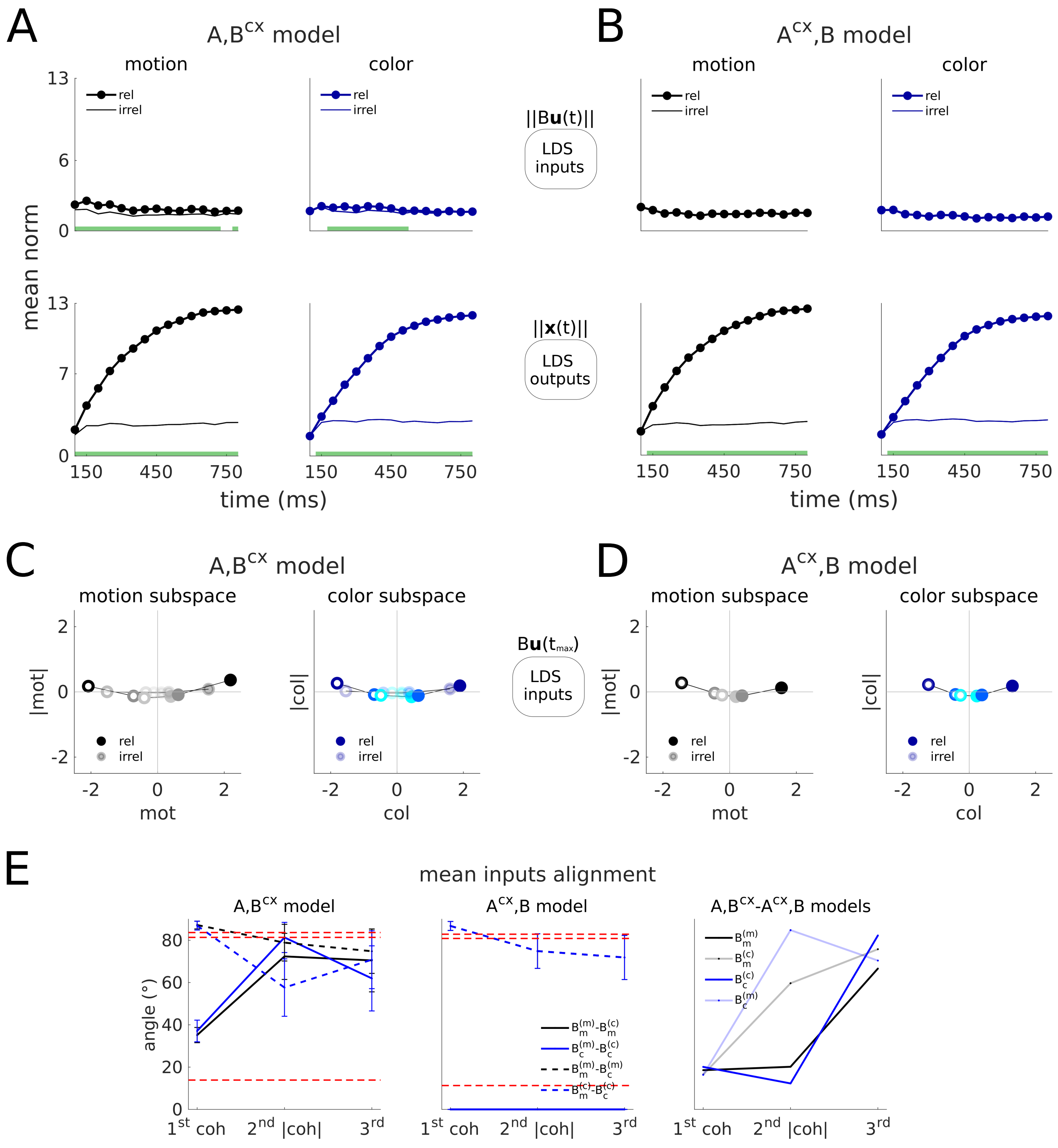

A
A,B^cx model
motion
color
rel
irrel
mean norm
13
6
0
7
150
450
750
time (ms)
B
A^cx,B model
||Bu(t)||
LDS inputs
||x(t)||
LDS outputs
C
A,B^cx model
motion subspace
color subspace
|mot|
|col|
mot
col
2
0
-2
Bu(t_max)
LDS inputs
D
A^cx,B model
E
mean inputs alignment
A,B^cx model
A^cx,B model
A,B^cx-A^cx,B models
angle (°)
80
60
40
20
0
1st coh
2nd |coh|
3rd
B_m^(m)-B_m^(c)
B_c^(m)-B_c^(c)
B_m^(m)-B_c^(m)
B_m^(c)-B_c^(c)
B_m^(m)
B_m^(c)
B_c^(c)
B_c^(m)

**Fig. S15. The LDS inputs inferred from the RNN data are largely constant over time and one-dimensional.** (**A-D**) Same as Fig. 4B-E but for the RNN data. (**A,B**) Inputs are learned nearly flat in both the $\{A, B^{cx}\}$ and $\{A^{cx}, B\}$ models, and almost identical across contexts in the $\{A, B^{cx}\}$ model, which is consistent with the ground truth RNN inputs being time-constant and fixed across contexts (Methods). Yet, both models, including the $\{A, B^{cx}\}$ model, strongly amplify the relevant inputs across contexts, but not the irrelevant ones. Note that the relevant inputs are much more strongly amplified than the irrelevant ones in the RNN, as expected from an optimal selective integration strategy [1], but the same is not found in the PFC data, were the irrelevant outputs are prominent and persist throughout the trial (Fig. 4B,C). (**C,D**) In the RNN the coherence representations are largely 1D since very little coherence magnitude modulation exists, unlike what is found for the PFC data (Fig. 4D,E). This is consistent with the ground truth RNN inputs being 1D and lacking a coherence magnitude component. (**E**) Same as Fig. S3C but for the RNN data. The alignments of the second inferred dimension (coherence magnitude) are not consistent across contexts in the $\{A, B^{cx}\}$ model (first panel), and neither across models for the irrelevant inputs (third panel, transparent lines), unlike what is found for the PFC data (Fig. S3C). Similar weak alignments are observed for the third input dimension, both in the RNN and the PFC data. This indicates that the coherence magnitude dimension in the RNN is not an invariant input feature. Instead, the additional input dimensions might be learned to compensate for the linear approximation.

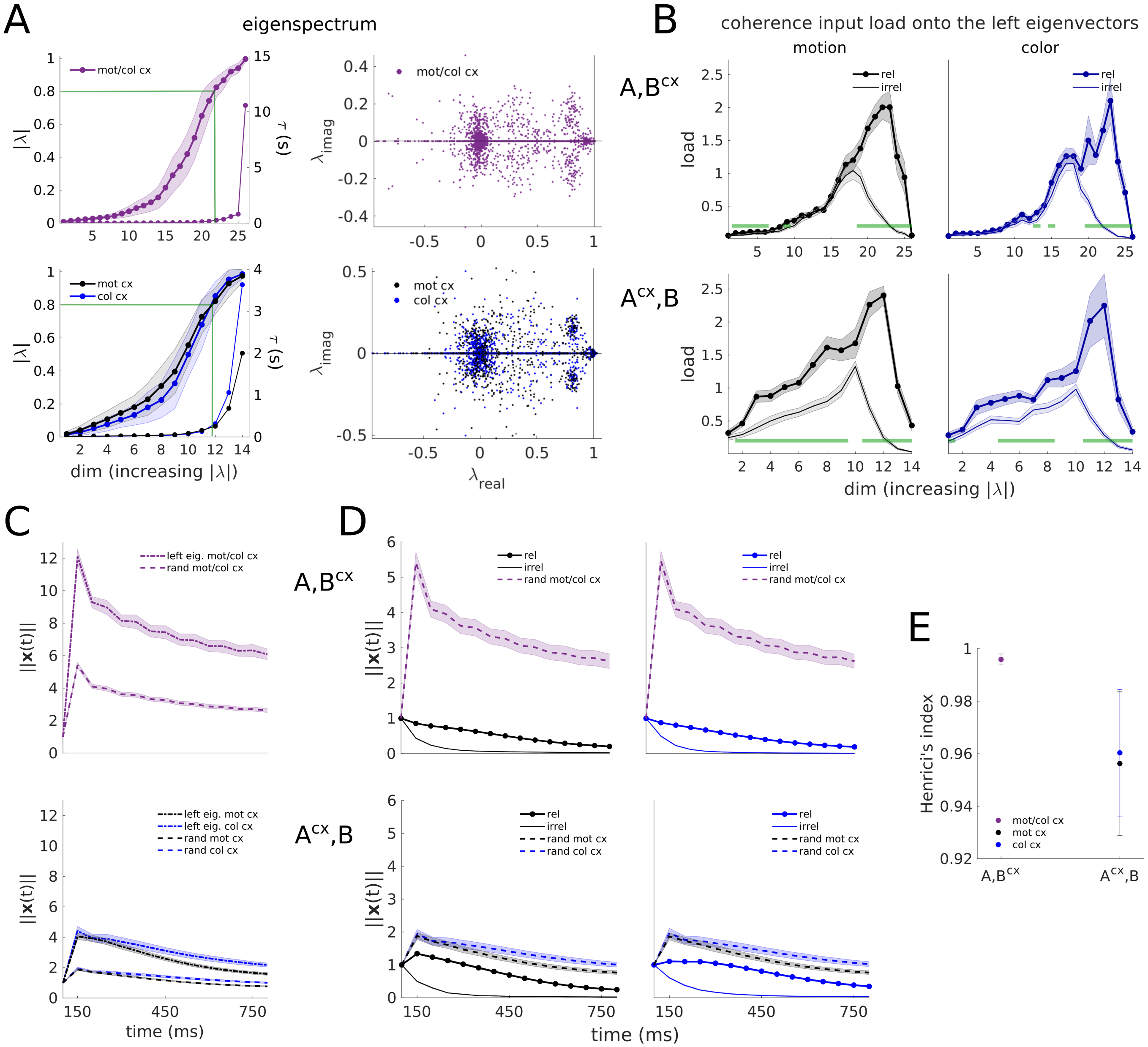
A
eigenspectrum
mot/col cx
|λ|
τ (s)
λimag
mot cx
col cx
dim (increasing |λ|)
λreal
B
coherence input load onto the left eigenvectors
motion
color
A,B^cx
A^cx,B
load
rel
irrel
dim (increasing |λ|)
C
left eig. mot/col cx
rand mot/col cx
||x(t)||
left eig. mot cx
left eig. col cx
rand mot cx
rand col cx
time (ms)
D
time (ms)
E
Henrici's index
mot/col cx
mot cx
col cx

**Fig. S16**. **The LDS dynamics inferred from the RNN data are high-dimensional, mediate relevant input selection largely through slow modes, and implement transient amplification of relevant inputs in the two models—but through extreme non-normality for the $\{A, B^{cx}\}$ model.** (**A,B**) Same as Fig. 5B,C and Fig. S6A, but for the RNN data. (**A**) The LDS models inferred from the RNN data are higher dimensional than expected for an idealized line attractor solution, with a single slow dimension and the rest of the dimensions fast decaying [16]. In particular, the LDS models learned several slow modes ($|\lambda| > 0.8$, green lines), as happened in the LDS data (Fig. 5B). However, the LDS models inferred from the RNN data had a smaller fraction of slow modes than the ones inferred from the PFC data ($\{A, B^{cx}\}$, $20 \pm 5\%$; $\{A^{cx}, B\}$, $21 \pm 7\%$ mot cx/ $22 \pm 5\%$ col cx, mean±std across 100 models; vs. $35 - 55\%$ in the PFC data). Furthermore, for the rest of the modes, most of them were very fast decaying ($|\lambda| < 0.4$, $\tau < 55$ms), unlike what was found in the PFC data, where most of the modes had either intermediate ($|\lambda| = 0.4 - 0.8$, $\tau = 98 - 224$ms) or slow eigenvalues ($|\lambda| > 0.8$). The largest eigenvalue was $1.00 \pm 0.02$ for the $\{A, B^{cx}\}$ model and $0.98 \pm 0.04$ / $0.99 \pm 0.04$ (mot/col cx) for the $\{A^{cx}, B\}$ model. The second largest eigenvalue was $0.94 \pm 0.03$ for the $\{A, B^{cx}\}$ model and $0.93 \pm 0.08$ / $0.95 \pm 0.06$ (mot/col cx) for the $\{A^{cx}, B\}$ model. The additional slow modes could have been learned to capture the curvature of the line attractor [1], or alternatively, to capture CI and contextual variance, as found in the PFC data (Fig. S9). Another possibility is that the higher dimensionality is a necessary feature of the mapping between low-rank linear RNNs and LDS models [75] (note that the RNN dynamics was low-rank, and approximately linear in each context [1]). Similarly, this might also in part explain the dimensionality of the LDS models inferred from the PFC circuit. (**B**) In both models, the coherence inputs inferred are most strongly loaded onto slow modes, rather than intermediate modes as in the PFC data (Fig. 5C). However, the inputs do not preferentially load onto the slowest modes ($|\lambda| > 0.9$, $\tau > 475$ms), as we found in the data—but in the RNN the slowest modes carry CI signals, rather than decision-related CD ones (Fig. S17) (**C-E**) Same as Fig. 6A-C but for the RNN data. (**C**) Left-eigenvector perturbations as well as random perturbations result in very strong amplification for the $\{A, B^{cx}\}$ model, unlike what is found in the PFC data (Fig. 6A). Accordingly, the degree of non-normality for this model is extremely high ((**E**)), unlike what is found in the PFC data (Fig. 6C). On the contrary, the response behavior of the $\{A^{cx}, B\}$ fitted to the RNN data is similar to the behavior in the PFC data (Fig. 6A). (**D**) Random perturbations are very strongly amplified by the $\{A, B^{cx}\}$ model, but the inputs are not. This indicates a high degree of specificity in the model, which might imply fine-tuning. The $\{A^{cx}, B\}$ model, on the contrary, processes inputs in a similar way as found in the PFC data (Fig. 6B), with the difference that the relevant inputs in this case are transiently ”amplified”, rather transiently ”persistent”. This might be simply due to the fact that for the RNN data the inputs are learned much weaker than for the PFC data (Fig. 4C, Fig. S15B), but the input pulse is provided with the same strength in both cases (unit norm). Also, the $\{A^{cx}, B\}$ model is slightly more non-normal for the RNN data ((**E**)) than the PFC data (Fig. 6C). This relatively high level of non-normality might also indicate some level of fine-tuning due to model mismatch—which might as well apply to the models from the PFC data, but to a lesser degree.

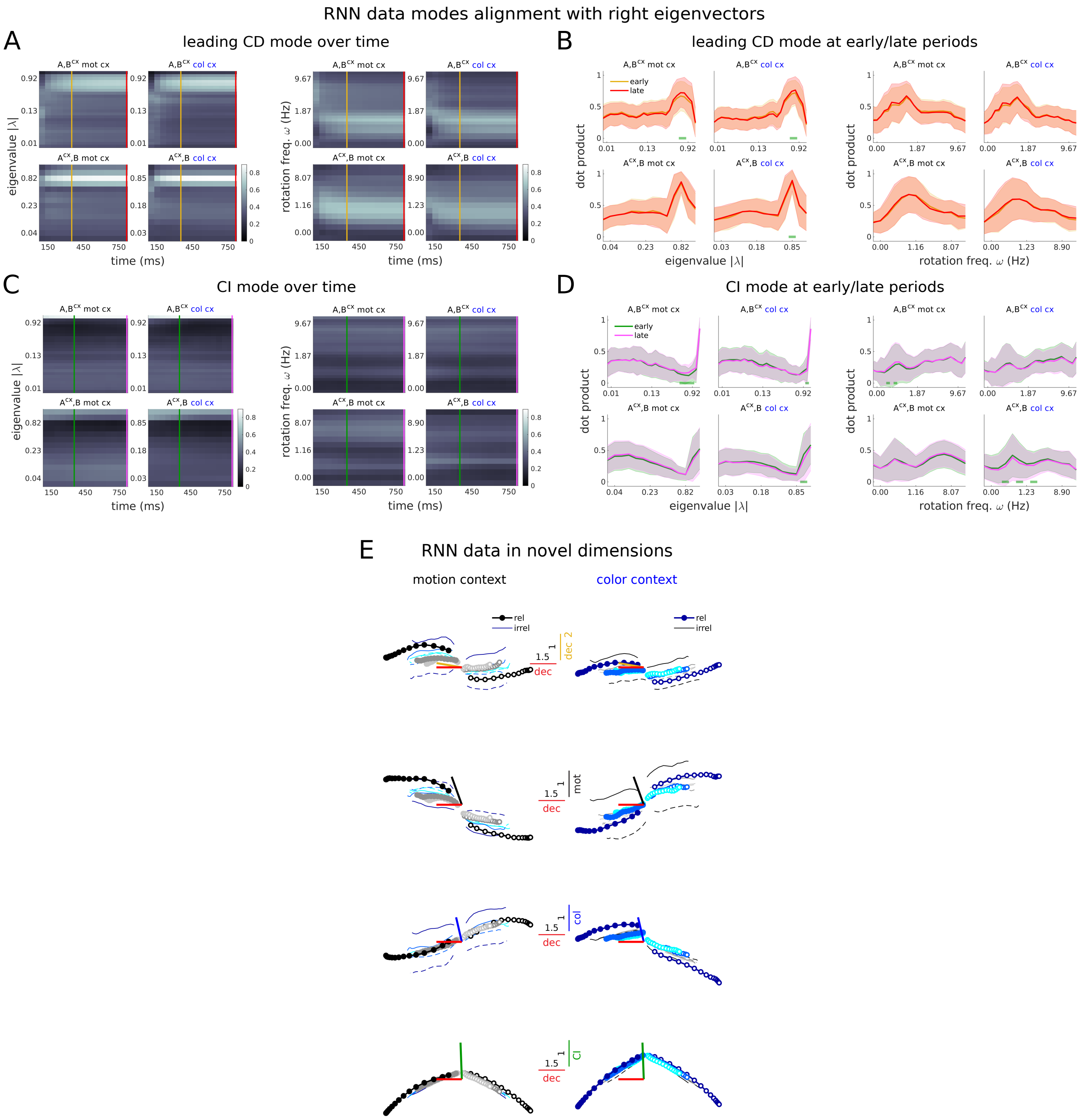
RNN data modes alignment with right eigenvectors
A
leading CD mode over time
B
leading CD mode at early/late periods
C
CI mode over time
D
CI mode at early/late periods
eigenvalue |λ|
rotation freq. ω (Hz)
time (ms)
dot product
early
late
E
RNN data in novel dimensions
motion context
color context
rel
irrel
dec 2
dec
mot
col
CI
1.5
1

**Fig. S17**. **The RNN integration process did not separate in two phases.** (**A-D**) Same as Fig. 7, Fig. S7, but for the RNN data. (**A,B**) The largest CD mode for the RNN data projects to slow modes throughout the whole trial, unlike what is found in monkey A's PFC data, where the CD vector projects to relatively fast decaying modes first and to the slowest modes later in the trial. Thus, the CD projection pattern in the RNN does not change over the course of the trial. Indeed, the distributions of projection early vs. late in the trial are practically indistinguishable (absence of green bars in (**B**), Wilcoxon rank-sum test, p$<$0.05). The CD vector aligns to slow modes ($|\lambda| > 0.8$), but not preferentially to the slowest modes ($|\lambda| > 0.9$), unlike what is found in monkey A's PFC data. The dynamics was also largely non-rotational ($\approx$1Hz), and not significantly different early vs. late in the trial (right panels in (**B**)). (**C,D**) The largest CI mode for the RNN data projects most strongly to the slowest modes, unlike what is found for monkey A's PFC data, where the CI vector targets slow modes, but not the slowest. (**E**) Same as Fig. 8B, but fore the RNN data. Top, the dimension inferred early in the trial highly aligns to the decision axis (small angle between red and yellow bars). This is consistent with the fact that early in the trial the RNN CD vector already projects to slow modes ((**A,B**)), and not to a different set of dimensions (the relatively fast decaying modes), as is found in monkey A's PFC data, which define a secondary decision dimension (Fig. 8B). Middle panels, trajectories in the LDS motion and color input coherence dimensions found from the RNN data fits. The coherence input vectors found by the LDS models are only moderately aligned with the ground truth RNN input vectors ($\{A, B^{cx}\}$: mot = 45°, col = 55°, for mean coherence input dimensions across 100 models and across contexts; $\{A^{cx}, B\}$, mot = 43°, col = 54°, for mean across 100 models), but higher than expected by chance (Fig. S6B). However, the RNN trajectories along the LDS coherence input dimensions (middle panels) are qualitatively similar to the trajectories along the ground truth RNN input vectors, and separate coherence information similarly well (Fig. S14B).

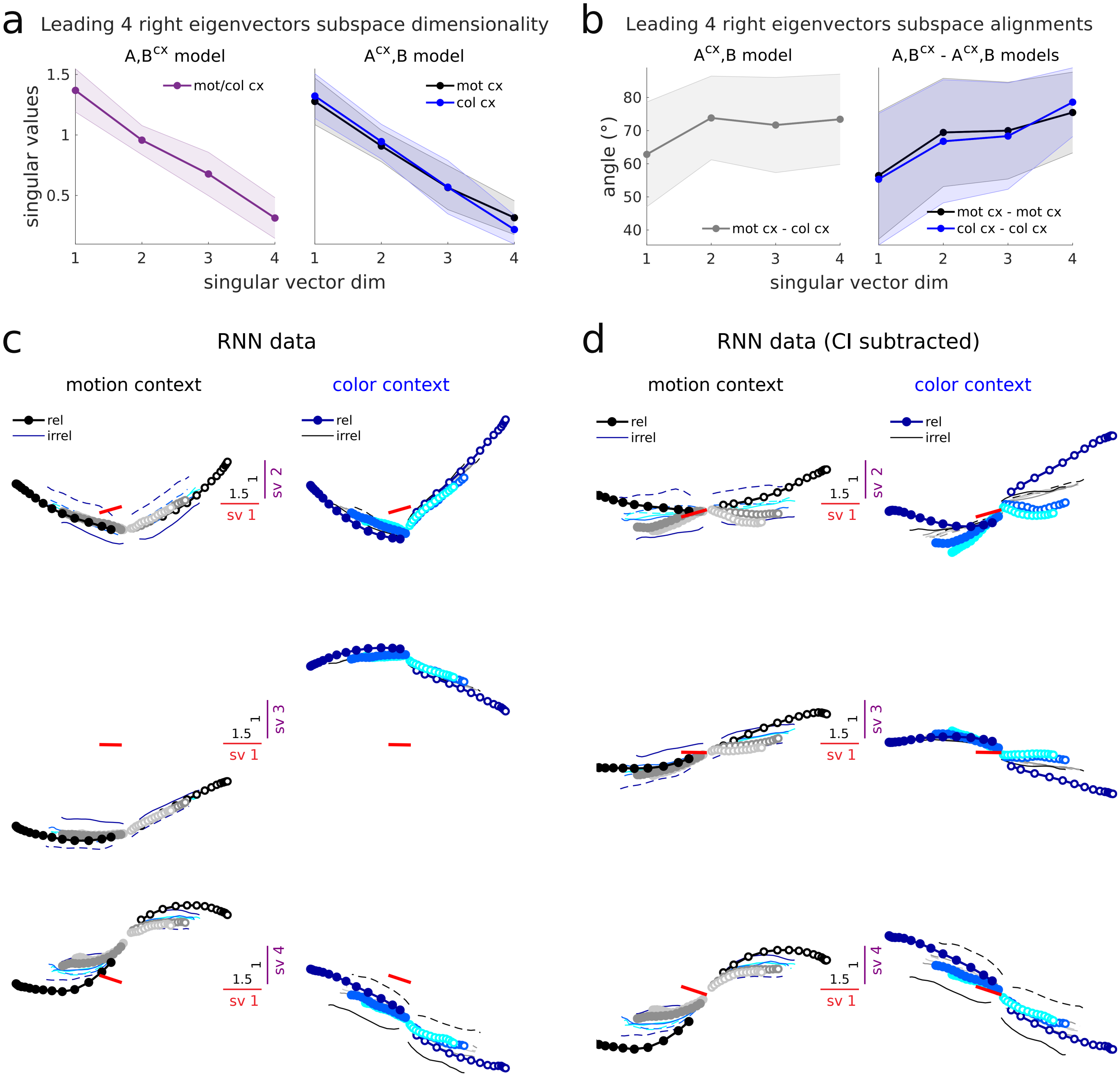
a
Leading 4 right eigenvectors subspace dimensionality
A,B^cx model
A^cx,B model
mot/col cx
mot cx
col cx
singular values
singular vector dim
b
Leading 4 right eigenvectors subspace alignments
A^cx,B model
A,B^cx - A^cx,B models
angle (°)
mot cx - col cx
mot cx - mot cx
col cx - col cx
singular vector dim
c
RNN data
motion context
color context
rel
irrel
sv 1
sv 2
sv 3
sv 4
d
RNN data (CI subtracted)
motion context
color context
rel
irrel

**Fig. S18**. **Choice signals in the slowest LDS subspace largely evolve along a single dimension across contexts in the RNN data.** Same as Fig. S9, but for the RNN data. (**A,B,**) The right eigenvectors effectively span four dimensions, since the singular values are not close to zero, as is found in the PFC data. Furthermore, the first first singular vector dimension is also the most aligned across contexts in the $\{A, B^{cx}\}$ model and across models. (**C,D,**) The first singular vector dimension (sv) captures decision information. However, sv dimensions 2 to 4 mostly capture condition independent (CI) variance and contextual variance. Indeed, only the first dimension aligns well with the RNN decision dimension (red bars), which is defined by the RNN output vector or readout after training [1] (sv1: $61°$, sv2: $85°$, sv3: $90°$, sv4: $84°$). Thus, the RNN trajectories mainly evolve along a single dimension in this 4D subspace, which aligns with the decision (or output) axis of the network. The other dimensions are used to capture the curvature of the approximate line attractor (sv4), CI features (sv2), and contextual separation (sv3, sv4).

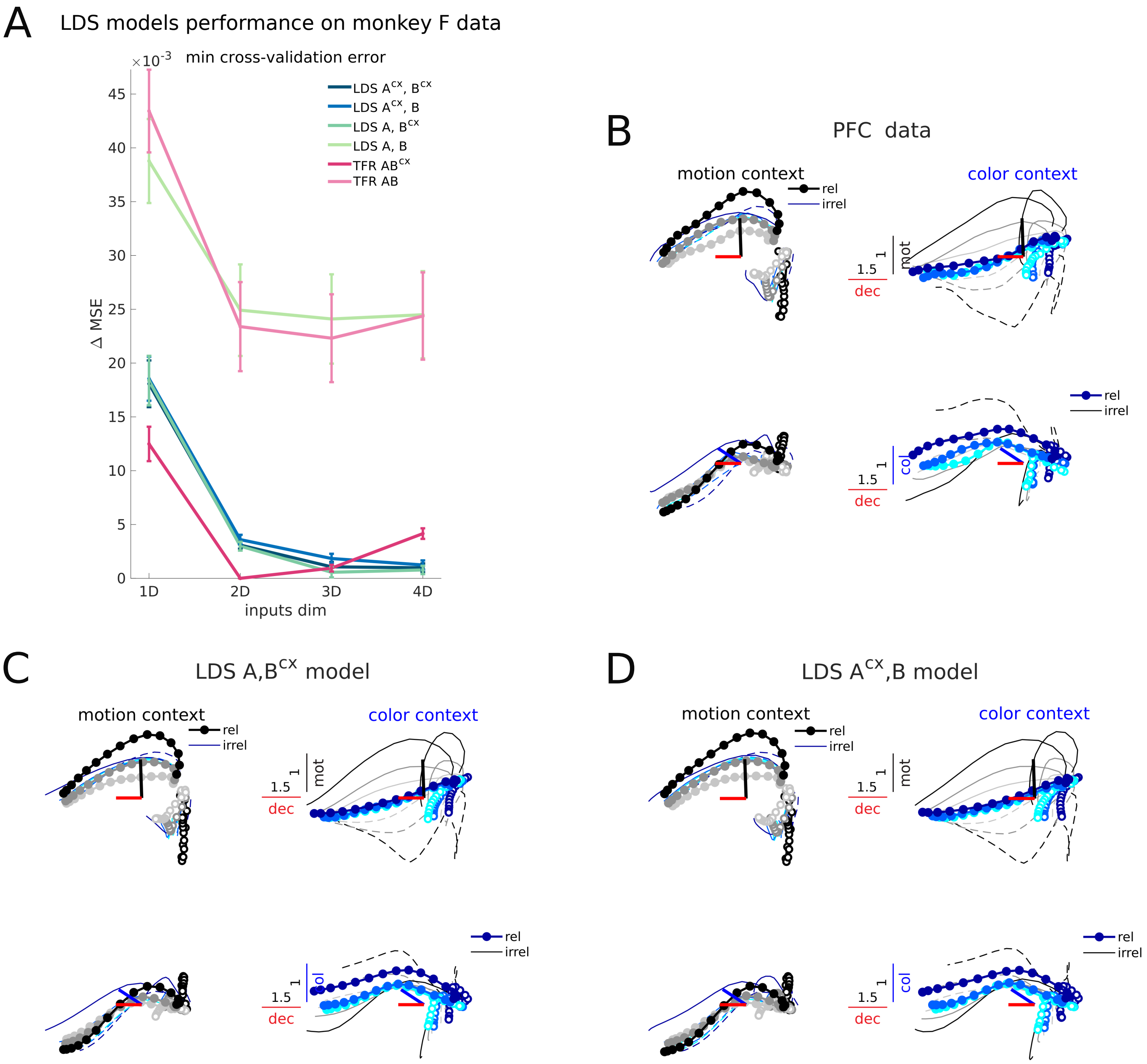


**Fig. S19**. **The LDS models accurately capture monkey F's data, perform comparably to TFR and require multi-dimensional inputs.** Same as Fig. S14, but for monkey F data. (**A**) The pattern of errors across models and input dimensions closely follows the one obtained for monkey A (Fig. 2A). The best performing model is also the $\{A, B^{cx}\}$ model with 3D inputs. The $\{A^{cx}, B\}$ model with 3D inputs performs similarly well (Table S3). (**B-D**) Same as Fig. 3B-D but for monkey F data. The LDS models accurately capture the trajectories in the contextually-stable task-relevant subspace. Note the presence of strong condition independent (CI) signals along the choice axis [1], which are also captured by the models.

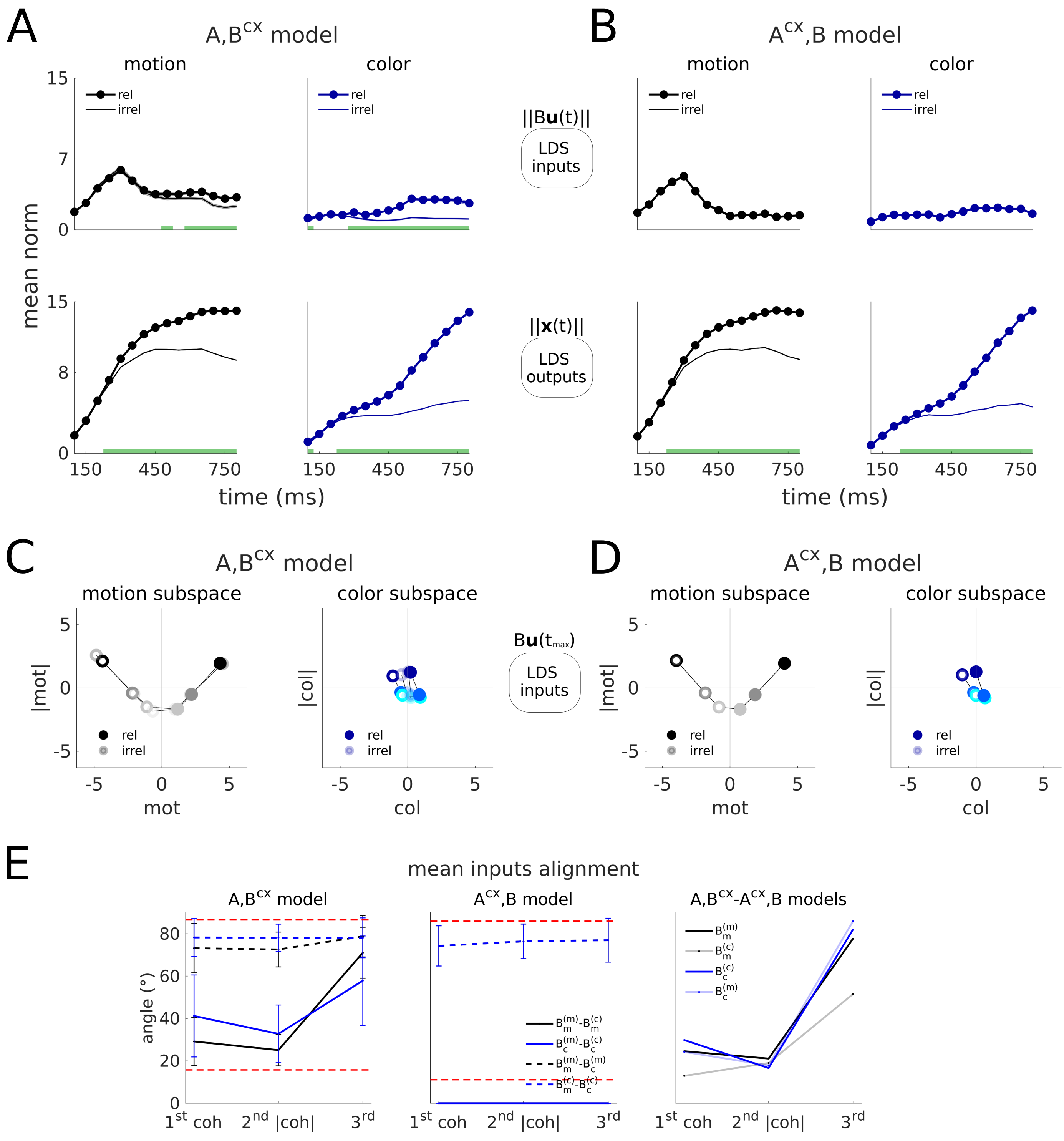
A
A,B$^{cx}$ model
motion
color
rel
irrel
mean norm
15
7
0
8
150
450
750
time (ms)
||B**u**(t)||
LDS inputs
||**x**(t)||
LDS outputs
B
A$^{cx}$,B model
C
motion subspace
color subspace
|mot|
|col|
mot
col
5
-5
B**u**($t_{max}$)
D
E
mean inputs alignment
A,B$^{cx}$-A$^{cx}$,B models
angle (°)
80
60
40
20
1$^{st}$ coh
2$^{nd}$ |coh|
3$^{rd}$
$B_m^{(m)}$-$B_m^{(c)}$
$B_c^{(m)}$-$B_c^{(c)}$
$B_m^{(m)}$-$B_c^{(m)}$
$B_m^{(c)}$-$B_c^{(c)}$
$B_m^{(m)}$
$B_m^{(c)}$
$B_c^{(c)}$
$B_c^{(m)}$

**Fig. S20**. **Monkey F's LDS inputs are largely stable across contexts and span curved manifolds, with motion inputs being strongly integrated in both contexts.** Same as Fig. S15, but for monkey F data. (**A,B**) The motion inputs from both models are similar to the inputs inferred for monkey A, in that these are transient in both the $\{A, B^{cx}\}$ and $\{A^{cx}, B\}$ models, but more sustained in the $\{A, B^{cx}\}$ model (top left panels). However, the $\{A, B^{cx}\}$ model infers motion inputs that are nearly identical in strength across contexts ((**A**), top left), unlike what is found for monkey A (Fig. 4B). Accordingly, the motion inputs are strongly integrated in both contexts, with the relevant outputs being only slightly stronger than the irrelevant ones ((**A**), bottom left). Another difference is that the inferred color inputs are very weak, although these increase slightly towards the end of the trial when relevant ((**A**), top right). Yet, the $\{A, B^{cx}\}$ model selectively and strongly integrates the color inputs ((**A**), bottom right). Both models generate identical outputs ((**A,B**), bottom). The color outputs increase more sharply in the middle of the trial, and continue growing until the end of the trial. On the contrary, motion outputs saturate towards the end of the trial (bottom left). This saturation is also observed in monkey A, for both the motion and the color outputs (Fig. 4B,C). Thus, color inputs might be integrated later in the trial in monkey F. An alternative explanation could be that color signals arriving into monkey F's PFC circuit are already integration signals, and our LDS models learn this particular input-ouput solution due to the fact that they incorporate an input penalty encouraging weak inputs (Methods). In line with this interpretation, we found that the LDS coherence input dimension is highly aligned with the decision dimension (Fig. S22E). (**C,D**) The motion coherence representations inferred by both models are strongly curved in this monkey, unlike for monkey A. The color inputs are weak along both the coherence and the coherence magnitude dimensions. (**E**) The pattern of alignments between the different input dimensions across models and contexts is consistent across monkeys. The inferred coherence and coherence magnitude dimensions for both motion and color are largely stable across contexts in the $\{A, B^{cx}\}$ model, but not the third dimensions (filled lines). One difference is that the motion coherence magnitude dimension is highly aligned across contexts in the $\{A, B^{cx}\}$ model in this monkey, but less so in monkey A (Fig. S3C). This is consistent with the motion coherence representations having a stronger curvature in monkey F than in monkey A ((**C,D**) vs. Fig. 4D,E).

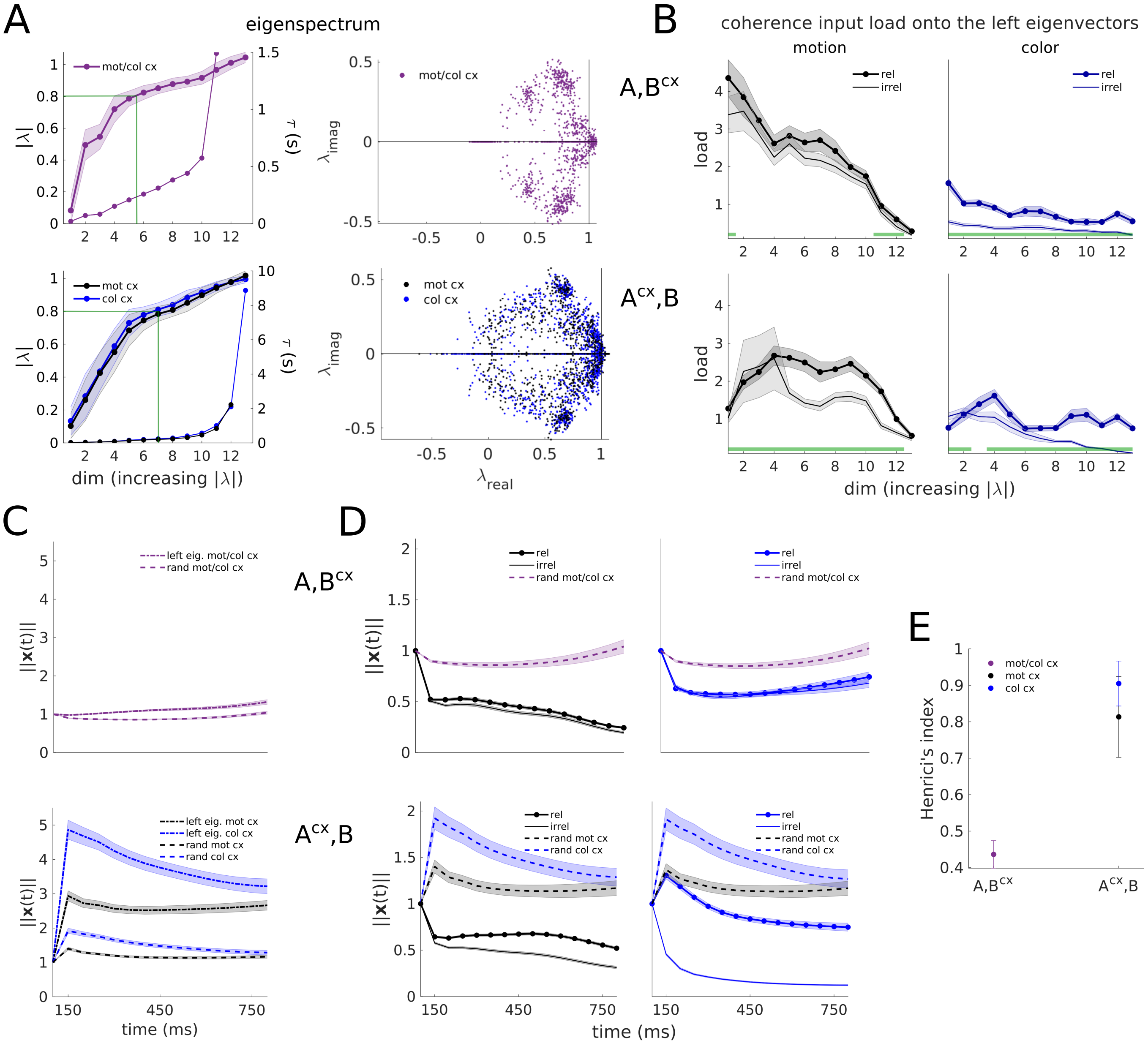
A
eigenspectrum
B
coherence input load onto the left eigenvectors
motion
color
mot/col cx
mot cx
col cx
dim (increasing |λ|)
A,B^cx
A^cx,B
rel
irrel
load
C
D
E
left eig. mot/col cx
rand mot/col cx
left eig. mot cx
left eig. col cx
rand mot cx
rand col cx
||x(t)||
time (ms)
Henrici's index

**Fig. S21. Monkey F's linear dynamics are high-dimensional, perform context-dependent input selection through several intermediate and slow modes and implement transient amplification of relevant inputs only in the $\{A^{cx}, B\}$ model.** Same as Fig. S16, but for monkey F data. (**A**) The inferred eigenspectrum from both models presents multiple slow modes, as in monkey A. The $\{A, B^{cx}\}$ model had also a larger fraction of slow modes than the $\{A^{cx}, B\}$ model (63 $\pm$ 7% vs. $46 \pm 8\%$ mot cx/ $53 \pm 8\%$ col cx, mean±std across 100 models). Note that the last two eigenvalues of the $\{A, B^{cx}\}$ model are slightly larger than 1 (unstable), so the time constant is not shown. Same for the last eigenvalue of the $\{A^{cx}, B\}$ model in the motion context. (**B**) The coherence inputs are not preferentially loaded onto the slowest modes, but rather, intermediate and fast modes, as in monkey A's data. However, the $\{A, B^{cx}\}$ model loads the motion inputs selectively only onto the second and third slowest modes (green bar). (**C**) In the $\{A, B^{cx}\}$ model the average impulse response across random perturbations grows over time, indicating the presence of unstable dynamics (top), which was not found in monkey A. On the contrary, the $\{A^{cx}, B\}$ model exhibited similar transient responses as monkey A (bottom). (**D**) The instability effect is also observed for perturbations along the color input dimension (top right panel). This feature might help integrate color input signals selectively, given that color inputs are very weak (Fig. S20A). The $\{A^{cx}, B\}$ model response to motion and color pulses is broadly similar to that of monkey A's. However, the responses were more amplified for the relevant color inputs in this monkey, and the relevant motion inputs exhibited transient amplification effects later in the trial, rather than immediately after the pulse. (**E**) The degree of non-normality of the LDS models is similar across monkeys (Fig. 6C).

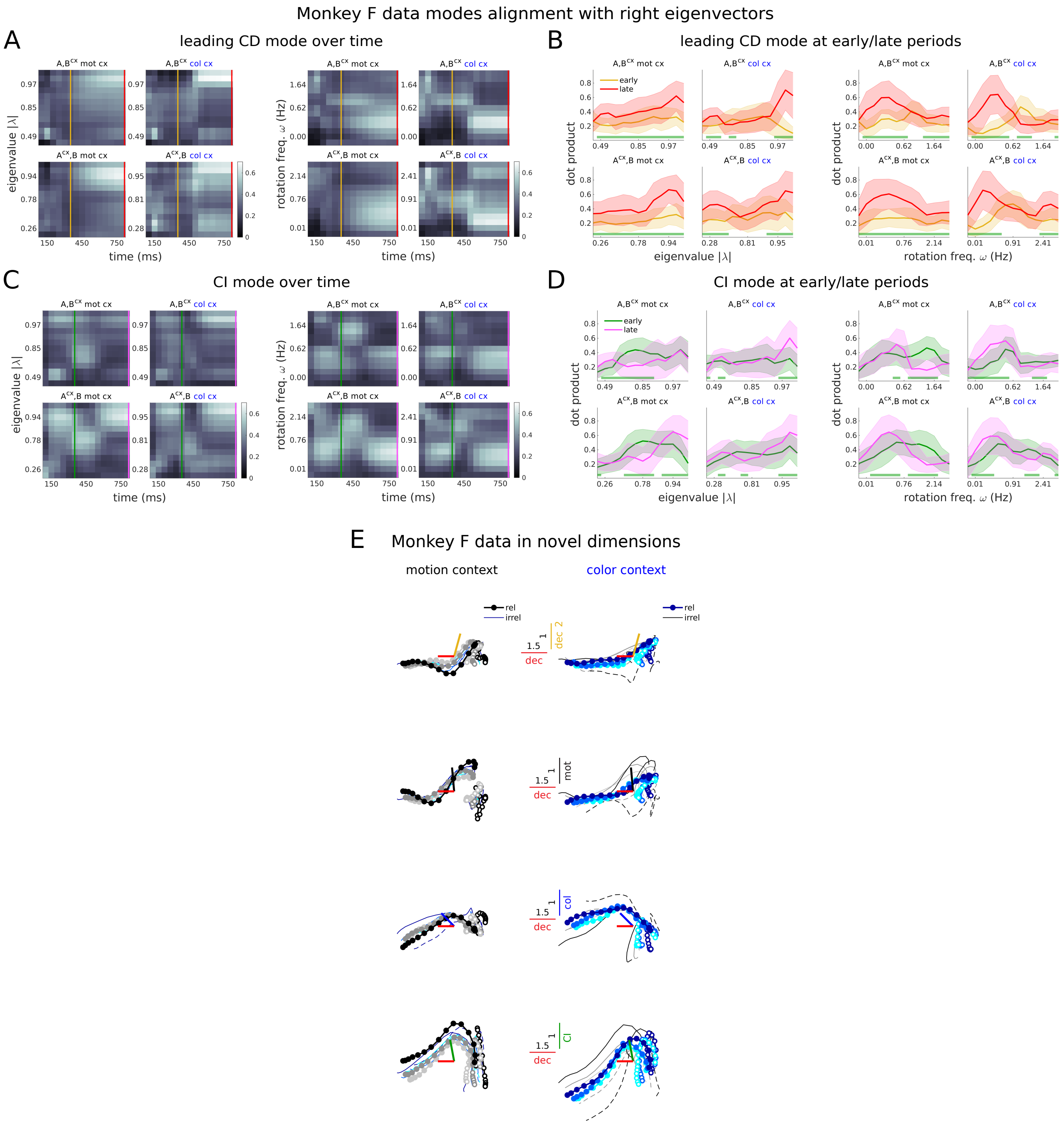
Monkey F data modes alignment with right eigenvectors
A
leading CD mode over time
B
leading CD mode at early/late periods
C
CI mode over time
D
CI mode at early/late periods
eigenvalue |λ|
rotation freq. ω (Hz)
time (ms)
dot product
early
late
A,B^cx mot cx
A,B^cx col cx
A^cx,B mot cx
A^cx,B col cx
E
Monkey F data in novel dimensions
motion context
color context
rel
irrel
dec
dec 2
mot
col
CI

**Fig. S22**. **Monkey F's integration process did less clearly separate in two phases, and CI signals were integrated along with choice signals.** Same as Fig. S17, but for monkey F data. (**A,B**) Monkey F did not present a clear separation of the integration process into different phases, unlike monkey A. In particular, early in the trial the leading CD variance vector did not preferentially project onto relatively fast decaying modes ((**B**), left panels, yellow lines, the pattern of projections is nearly flat), unlike for monkey A (Fig. 7B, Fig. S7B, left panels, yellow lines, projections pick at intermediate modes). However, the pattern of early vs. late projections was similar to that of monkey A when splitting the modes by their rotation frequency ((**B**), right panels, yellow lines in the color context, and in the motion context for the $\{A, B^{cx}\}$ model, pick on intermediate rotation frequencies, as for monkey A, Fig. 7B, Fig. S7B, right panels). The modes targeted during the late phase of the trial (red line projections) are consistent across monkeys both in time constant and rotation frequency. This late projections significantly differ from the early projections (green bars in (**B**), Wilcoxon rank-sum test, $p<0.001$). (**C,D**) The CI data vector for monkey F projects most strongly onto the slowest modes, in particular at the end of the trial ((**D**), pink lines). This indicates that CI signals are integrated along the same dimensions as the CD signals, i.e., the dimensions that integrate coherence inputs (compare with red lines in (**B**)), unlike what is found for monkey A (Fig. 7C,D, Fig. S7C,D, left panels, pink lines pick onto slow modes, but not the slowest). This is consistent with the fact that CI signals are strongly present along the decision axis [1]. The block-like structure found in the pattern of projections in panels (**A,C**) comes from the fact that the variance along the first singular vector dimensions is close to the variance along the next dominant singular value dimensions (i.e. that the covariance structure of the data is largely spherical), which leads to switches in the estimation of the dominant variance direction. (**E**) The early-phase dimension or secondary decision dimension (yellow) does not capture much structure of the trajectories in monkey F (top). The late-phase dimension (red) is strongly aligned with the TDR decision axis (18°). The LDS coherence input dimensions were well aligned with the TDR dimensions in the $\{A^{cx}, B\}$ model, specially for color, but not in the $\{A, B^{cx}\}$ model ($\{A, B^{cx}\}$: mot = 54°, col = 71°, for mean coherence input dimensions across 100 models and across contexts; $\{A^{cx}, B\}$, mot = 44°, col = 31°, for mean across 100 models). The averaged coherence input dimension across contexts and models highly aligned with the decision dimension (angle between blue and red bars in middle panels). This alignments were higher than expected by chance (Fig. S6B).

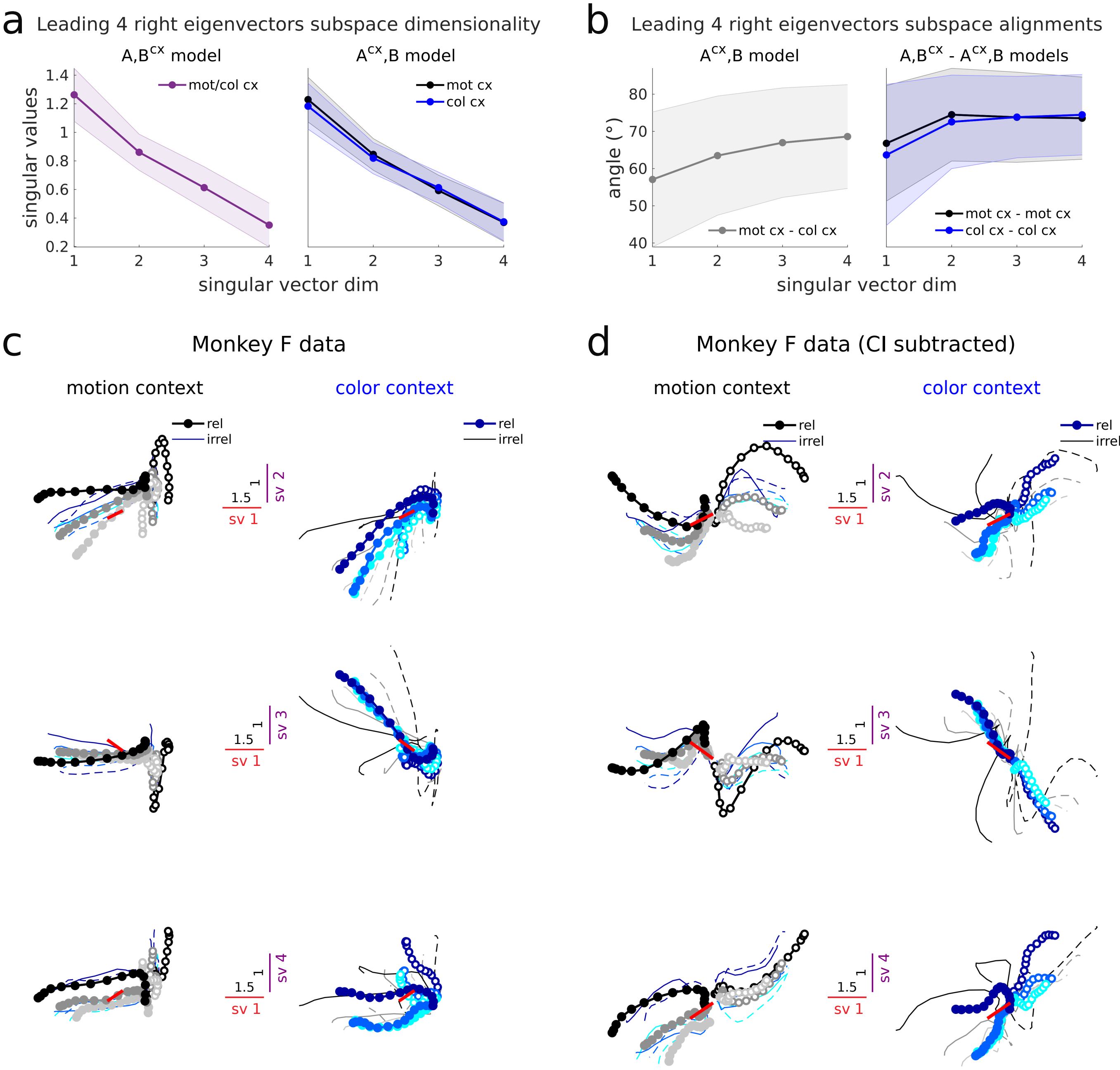


**Fig. S23**. **Choice signals in the slowest LDS subspace largely evolve along a single dimension across contexts in monkey F's data.** Same as Fig. S18, but for monkey F data. (**A,B,**) The right eigenvectors effectively span four dimensions, since the singular values are not close to zero, as found in the other monkey. Furthermore, the first singular vector dimension is also the most aligned across contexts in the $\{A, B^{cx}\}$ model and across models (Fig. S9A,B). (**C,D,**) The first singular vector dimension (sv) captures decision information. However, sv dimensions 2 to 4 mostly capture condition independent (CI) variance and contextual variance. Indeed, only the first dimension aligns well with the TDR decision dimension (red bars) (sv1: 58°, sv2: 80°, sv3: 75°, sv4: 76°).

# Supplementary Text

## Additional LDS model-fitting controls

### LDS models with time-constant inputs

To rule out the possibility that the LDS models were learning dynamically complex external input signals $\mathbf{u}_{m,c}(t)$ to capture the data, we re-trained all input parameters and constrained $\mathbf{u}_{m,c}(t)$ to be constant in time $\mathbf{u}_{m,c}$. This resulted in a relatively small drop in performance (Fig. S5A, for models with input dimensionalities higher than 2D). In particular, the performance of re-trained models with 3D constant inputs (dashed lines) dropped to the level of the 1D time-varying input models (filled lines). Newly fitted models with $\mathbf{u}_{m,c}$ constant in time, but where all parameters were optimized, performed nearly as well as the time-varying models (Fig. S5B), and accurately captured the PFC trajectories (Fig. S2G,H). Notably, in these two control model classes the optimal input dimensionality was consistently 3D, and the latent dimensionalty was also close to the time-varying input models' dimensionality ($\{A^{cx}, B\}$ - $\{A, B^{cx}\}$, dim = 16-22 for the time-varying models with inputs retrained to be time-constant; 15-16 for the newly optimized time-constant models and 16-18 for the original time-varying models). Most importantly, the time-constant input models could only rely on their recurrent dynamics to capture the temporal complexity of the PFC data. Therefore, the complexity of the PFC responses is well approximated by linear dynamics and is not necessarily inherited from the external inputs' dynamics.

### Features of the PFC data captured by the LDS models

We also quantitatively assessed whether the time-constant input models were indeed able to capture complex temporal structure in the data. For this, we asked how well these models performed on time-shuffled data, which had no correlational structure across time. If there was no drop in performance, this would indicate that the time-constant input models were uniquely capturing time-unrelated structure, such as correlations across neurons and conditions. The performance of the best time-constant models (which had 3D inputs, Fig. S5B) dropped substantially, being worse than the 1D input models and nearly as bad as the most contextually constrained $\{A, B\}$ models (Fig. S5C). This indicates that the simple, time-constant LDS models indeed captured the complex time-related structure present in the PFC data. However, these models still captured a substantial fraction of the time-shuffled data variance (24% on shuffled data vs 27% on the original data). This suggests that the LDS models might be in gran part capturing correlational structure across neurons and conditions, besides time-related variance (which makes sense, given that the LDS is a low-d model that extracts common structure across neurons, and across conditions via shared inputs). Indeed, surrogate data sets randomized across conditions, neurons and time were very poorly captured, even by the best time-varying LDS models (Fig. S5D). These data sets were designed to preserve the primary statistics of the data, and thus these results also indicate that the LDS models were not merely capturing basic features of the data [13].

**Models constrained to have normal dynamics**

To prove that the non-normality of the dynamics is an important feature of the PFC data, we tested whether models that are not non-normal (i.e. that have normal dynamics) performed worse on the data. To enforce the LDS models to learn normal dynamics, we included the following penalty in our cost $AA^\intercal - A^\intercal A$, since any normal matrix $A$ must fulfill $AA^\intercal = A^\intercal A$. We also constrained $C$ to be orthonormal, to leave the normality properties of the dynamics unaltered when mapping the latents into the observations. Otherwise, given well-known degeneracies arising from a similarity transformation of the $(A, B, C)$ triplet, the pair $CA$ could result in identical fits as the ones from the non-normal models, even with a normal $A$, by "pushing" the non-normality into $C$ (e.g. through ill conditioning of $C$). To enforce orthogonality of $C$, we set this parameter to the one learned for the original non-normal models, which was orthogonalized post-hoc (Methods). This worked better than introducing an additional penalty to enforce the orthogonality of $C$, since it ensured that $C$ was completely orthonormal. Orthogonalizing $C$ post-hoc, after learning $A$ constrained to be normal, was problematic, given that the similarity transformation required in the orthonormalization procedure ($TAT^{-1}$, see Methods) affected the normality of $A$ post-hoc.

We tested the performance of the $\{A, B^{cx}\}$ and $\{A^{cx}, B\}$ models for two different weights $\lambda_{dyn}$ of the normality penalty (Fig. S5E,F). For $\lambda_{dyn} = 1$, the models still had some residual non-normality (Fig. S5G, Henrichi's index of the fitted A matrix = 0.025). With such values the performance of the $\{A, B^{cx}\}$ model was weakly affected, whereas the performance of the $\{A^{cx}, B\}$ model was severely affected (Fig. S5E). For a much stronger penalty weight, $\lambda_{dyn} = 1e5$, the Henrichi's index was virtually 0 (Fig. S5G), and the performance of both models was greatly impacted (Fig. S5F). These results support our original conclusion that both the $\{A, B^{cx}\}$ and the $\{A^{cx}, B\}$ models require some non-normality in the dynamics to explain the data, but to different degrees. Specifically, the $\{A^{cx}, B\}$ model requires stronger non-normality than that $\{A, B^{cx}\}$ model to account for the PFC responses.

**Parameter recovery**

We also performed a control to show that both model classes are fitting properly. To show this, we performed a parameter recovery analysis by simulating data from the best model of each class (out of the 100 models we fitted with random initializations), and then re-fitted 100 models from the same model class the data was generated from. We found that the re-fitted models:

1. Consistently achieve a very low MSE: $\{A^{cx}, B\} = (5 \pm 2)e^{-4}$, $\{A, B^{cx}\} = (3 \pm 2)e^{-4}$, mean±std, (Fig. S10A).

2. Recover the true inputs and latent dimensionalities (Fig. S10B,C).

3. Recover qualitatively the same solution as the true model, in terms of inputs and dynamics properties inferred (Fig. S10D-G, Fig. S11A-G).

Additionally, we fitted models from the wrong class (e.g. we fitted 100 models from the $\{A^{cx}, B\}$ class on data generated from the best $\{A, B^{cx}\}$ model). We show that these models, despite being from the wrong model class, still fit the data very well, i.e. the MSE error is very small: $\{A^{cx}, B\} = (1 \pm 1)e^{-3}$,

$\{A, B^{cx}\} = (6 \pm 2)e^{-3}$, mean±std. However, the true model class still performs better (See performance in point 1 above and Fig. S10A). This might not be surprising given that both model classes approximate the PFC data equally well, so in the parameter regime constrained by the PFC data, both models should be almost equivalent.

**Consistency of solutions**

The mean input and dynamics properties reported in the main manuscript (taken over 100 randomly initialized LDS models) are representative of the solution implemented by individual LDS model instances. We show this for the top 10 models, out of the 100 models we fitted:

1. The top 10 models learn solutions that are analogous to each other in terms of the qualitative properties of the inferred inputs and dynamics (Fig. S12A-J).
2. All 100 models, including the top 10 models, have very close performance. In particular, we show that the MSE distributions belonging to the 100 models from each model class are tightly peaked around their mean and do not overlap with each other (Fig. S12K).

We report solutions for the two main model classes discussed in our manuscript ($\{A, B^{cx}\}$) and ($\{A^{cx}, B\}$), as well as the ($\{A^{cx}, B^{cx}\}$) model class (see below), which achieves the same performance as the two other model classes.

## LDS models with context-dependent dynamics and inputs

A model combining contextual modulation of both inputs and internal dynamics ($\{A^{cx}, B^{cx}\}$) explains the data as well as the two main model classes analyzed in the manuscript (Fig. 2A), which had context-dependent inputs ($\{A, B^{cx}\}$) or context-dependent dynamics ($\{A^{cx}, B\}$), and thus should also be discussed. We analyze the properties of these models in Fig. S12. Notably, the $\{A^{cx}, B^{cx}\}$ model show aspects of both the $\{A, B^{cx}\}$ and $\{A^{cx}, B\}$ models, such as inputs that are moderately modulated across contexts (Fig. S12C,F) but that are not sustained over the trial (Fig. S12C), or dynamics with degrees of non-normality that are in between those of the two other model classes (Fig. S12J). Thus, in this sense, the $\{A^{cx}, B^{cx}\}$ model does not provide many more insights into the range of mechanisms that could potentially explain the data. However, this mixture of solutions is a plausible one, which raises the possibility that PFC might be relying on both mechanisms. Importantly, however, the fact that the $\{A^{cx}, B^{cx}\}$ model does also explain the data should not be taken as definite evidence that both inputs and dynamics are modulated by context. Conclusively establishing which of the model classes best captures the mechanisms of context-dependent computations in PFC will require testing experimental predictions of the kind we propose in this manuscript (Fig. 9 and Fig. S13).

## Understanding non-normal transient amplification

Transient amplification is a property of dynamical systems that have non-normal dynamics matrices [17, 18]. These systems present non-trivial dynamical properties that are not predicted by their steady state behavior.

In particular, such systems can transiently amplify inputs before decaying to a steady state. To illustrate how the transient amplification mechanism takes place, we built two simplified dynamical system models with identical specifications and only two dimensions (shown below), and made one normal (degree of non-normality or Henrici's index=0, see Methods) and the other highly non-normal (Henrici's index=0.8).

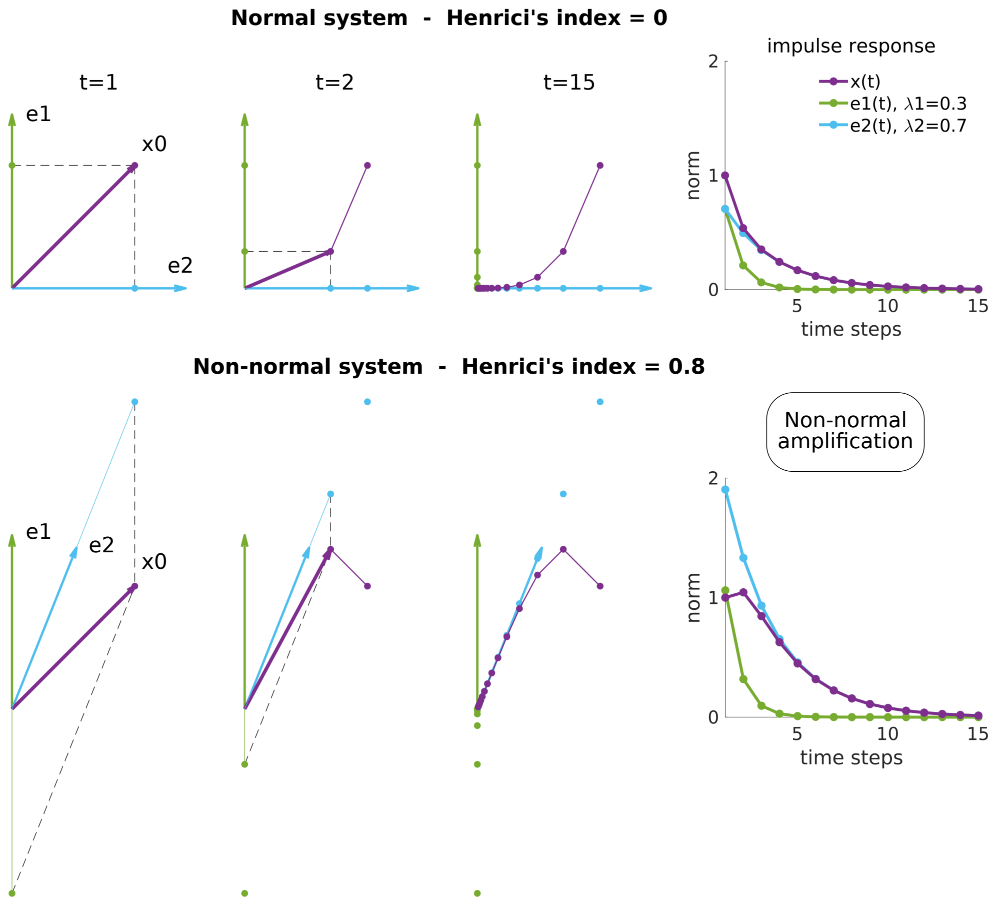


**Fig. S24. Non-normal amplification toy example.**

In the above figures, we show the two right eigenvectors of the dynamics for each system ($e1$, $e2$, in green and blue), which define the "output" dimensions were activity evolves over time. In the normal system, by definition, these are orthogonal vectors. In the non-normal system, they need not be, as it is the case in this example. Two additional left eigenvectors exist, but in a normal system they are the same as the right eigenvectors. In a non-normal system the left eigenvectors are distinct from the right ones (see Methods), but these are not shown here since they are not crucial to understand this picture. We set one of the eigenvalues of the system to be small ($\lambda 1 = 0.3$, fast dynamics) and the other large ($\lambda 2 = 0.7$, slower dynamics), in both models. We then analysed the impulse response properties of both systems (for t=15 time steps, as in the PFC data). For this, we provided an input pulse of unit norm along a random direction $x0$ (in this case, for illustration purposes, a direction that bisected the plane spanned by the two right eigenvectors in the normal system, see left panels, purple arrow). We then looked at how each system processed such input by looking at the evolution of the system state over time $x(t)$ and its norm (middle and right panels, purple

lines). In the normal system, the input decayed exponentially towards its steady-state, at zero, as expected from a dynamical system with real eigenvalues smaller than one (rightmost panels). The evolution of the two modes of the dynamics was governed by the time-constant of the eigenvalues (right panel). The smallest eigenvalue mode decayed faster (in green) and the largest slower (in blue), with the slower eigenmode largely determining the evolution of the state at later stages (in purple). The evolution along the two dynamics modes could be obtained by projecting the state at each time step onto the orthonormal basis defined by the two right eigenvectors (left and middle panels). The non-normal system, however, had non-orthogonal eigenvectors. In particular, we purposely set the eigenvectors to be closely aligned to each other, to obtain strong non-normal dynamics. This meant that the system state could no longer be decomposed using an orthogonal projection. Instead, the state was constructed from a linear combination of a non-orthogonal eigenvector basis set (left and middle panels, by applying the parallelogram law of vector composition). We provided the same input perturbation as for the normal system. Despite the similarities in design (in particular, having the same eigenvalues), the impulse response properties of this system were very different. Most notably, right after the pulse, there was a transient increase in the state norm (middle and right panels). However, the state eventually decayed to zero. This is because the long-term behaviour of a non-normal system is still governed by its eigenvalues. The smallest eigenvalue mode decayed faster and the largest slower, and they did so exponentially, as in the normal system (rightmost panel). Yet, initially a transient amplification effect was observed. This happens precisely because of the difference in the decay rates of the modes, combined with the fact that the state is constructed with a non-orthogonal eigenvector basis (left and middle panels, note how the state vector, in purple, is reconstructed at each time step). The more aligned the eigenvectors are, and the stronger the difference between their eigenvalues, the larger the degree of non-normal amplification. Note that because of the non-trivial decomposition of the state along the non-orthogonal basis, the modes' initial norm was very large (left and right panel, specially for the mode in blue). This resulted in the state experiencing a more sustained decay than in the normal system (right panel, compare blue and purple lines in both systems), i.e. the input pulse was transiently "persistent". This precise behavior was found for the relevant motion and color inputs in the $\{A^{cx}, B\}$ model, but not the irrelevant inputs; or any inputs in the $\{A, B^{cx}\}$ model (Fig. 6B).

## Biological implementation of the top-down input modulation mechanism: fixed input subspaces with context-dependent external inputs

The $\{A, B^{cx}\}$ mechanism can be realized even in the case where the input dimensions $B$ are fixed across contexts, which may be more intuitively aligned with biology—given that long-range projections are known to be anatomically stable. In this alternative view, the direction of input vectors $B\mathbf{u}$ within the fixed input subspace can still be changed, provided that the external inputs are context-dependent $\mathbf{u}^{cx}$, and that the dimensionality of the input subspace is higher than 2D. This can be achieved by changing the external inputs' strength in each context independently along each of the input dimensions (i.e. for 2D inputs, $u_1^{cx}$ and $u_2^{cx}$). This effectively changes the input vector $B\mathbf{u}$ coordinates within the input basis $B$. This change of coordinates can be used to rotate and stretch the inputs within the input subspace differently across contexts (i.e. $u_1^{cx}$ and $u_2^{cx}$ can be set to define any vector in the 2D input plane). The dimensionality of $B$ (its columns) determines the number of inputs that originate from different subpopulations—within an area or

across multiple areas. Changes in input direction could then be achieved through top-down modulation of the different subpopulations independently (i.e. modulation of subpopulation 1 would change $u_1^{cx}$ and of subpopulation 2 would change $u_2^{cx}$, resulting in a change of $B\mathbf{u}$ norm and direction).

## Perturbation analysis

In Fig. 9 and Fig. S13 we propose how to perturb population activity in PFC in order to disambiguate between the main model classes. These procedures could be readily performed in future experiments without the need to fit LDS models to the newly collected data. We specify the experimental steps needed to uncover the perturbation effect.

One should first record PFC population activity during a contextual discrimination task of the type implemented by Mante et al. [1]. Then compute per-condition averaged trajectories from the z-scored PSTHs under control conditions (i.e. without perturbations). Then, apply experimental perturbations to the activity (which should be brief, to be virtually unnoticeable and to not completely disrupt the dynamics), either by perturbing the activity directly (e.g. using electrical or optogenetic manipulations), or indirectly through input perturbations (e.g. by transiently changing the coherence of the random dots stimulus). Then, compute per-condition averages of the perturbed trajectories. Finally, subtract the averaged unperturbed trajectory to the perturbed trajectory. If the linearity assumption of the dynamics holds, this should reveal the effect of the perturbation. The effect can be visualized along specific state space dimensions (such as the task-relevant dimensions identified using TDR, as in Fig. 3) or by computing the norm of the trajectories' difference in the whole state space. In such case, we should see exactly a phenomenon of the type reported in Fig. 6A, when considering direct activity perturbations; i.e. we should see the transient amplification of the perturbation, if the dynamics falls within the $\{A^{cx}, B\}$ model class, but not the $\{A, B^{cx}\}$ class. We simulated the outcome of such experimental procedures using our fitted LDS models.

In the case of direct activity perturbations, we considered applying those along generic principal component dimensions, which could be easily computed from the control data in future experiments. In our simulations, the response to the perturbations were generated from the best performing models fitted to monkey A's data. In Fig. 9A, we show the results of executing the steps above for an example perturbation along PC 15 of the PFC data during the motion context. We provided this perturbation "transiently" for 50ms at t=100 after random dots motion onset, which in our case was the first time step modeled. The perturbation vector was of norm 10, chosen to elicit a strong enough response that could be visualized in the raw perturbed trajectories (Fig. 9A, right panels). For illustration purposes, we chose to project the model-generated trajectories along the decision dimension found from the data using TDR. By subtracting the perturbed and unperturbed trajectories, one can see that this type of perturbation has a strong effect along the decision dimension for the $\{A^{cx}, B\}$ model, but not for the $\{A, B^{cx}\}$ model (Fig. 9B, thick lines). Computing the norm of the trajectories' difference reveals a clear transient amplification of the perturbation for the $\{A^{cx}, B\}$ model, but not for the $\{A, B^{cx}\}$ model (Fig. 9C, thick lines). This is also the case for perturbations along other PC dimensions (thin lines). The response to the different PC perturbations is not so clearly distinguishable on average across 100 trained models along the decision dimension (Fig. 9D). The average response is however

clearly different across models when considering the norm of the perturbations (Fig. 9E), which reveals the transient perturbation effect characteristic of the $\{A^{cx}, B\}$ model, but not the $\{A, B^{cx}\}$ model (Fig. 6A). This transient amplification effect is observed for several PC dimensions. We found that the dimensions with stronger amplification did not tend to live in the leading PC dimensions, but rather, in PCs $>$10. In particular, for our best $\{A^{cx}, B\}$ model (out of the 100 models trained) we found that only a handful of dimensions resulted in transient amplification: PCs 13, 15, 16, 19, 20. This can be explained by the fact that the left eigenvectors in this model class, whose direct perturbation results in strong transients (Fig. 6A, Fig. S6G), are highly clustered (Fig. S6D).

An alternative way of choosing the directions along which to perturb the activity, although less practical to implement experimentally, would be to fit LDS models to the newly collected data to find the state-space directions along which we expect a perturbation to have maximal effect: this should be the directions specified by the left eigenvectors of the dynamics (see Fig. 6A, Fig. S6F, Fig. S6G).

In the case of input perturbations, we provided these in the model by transiently "switching off" the relevant inputs in each condition (by setting to zero the inputs along all input dimensions, 3 in this case). We did this during the third time point of the trial, representing 50ms at t=200ms after random dots onset. The perturbation could be implemented experimentally by transiently setting the relevant coherence value to $0\%$ for 50 ms at t=100ms after random dots onset (to account for response latencies to visual stimuli in FEF, the region in PFC where most of the recordings were performed [1], estimated to be $<$100ms [76]). Thus, the effect should be measurable in PFC at around t=200ms, as we simulated. In this case, the $\{A, B^{cx}\}$ model experienced a stronger response to the input perturbation than the $\{A^{cx}, B\}$ model (Fig. S13C,E), although the observed differences across models were weaker than for activity perturbations. This is line with the fact that the inferred inputs for the $\{A, B^{cx}\}$ model are stronger than the inputs inferred for the $\{A^{cx}, B\}$ model (Fig. 4B,C).

We also show the predictions of activity and input perturbations for the $\{A^{cx}, B^{cx}\}$ model (Fig. 9, Fig. S13). The results are, as expected based on the properties inferred for this third model class (Fig. S12J, Fig. S12A-F), in-between the perturbation effect for the two other model classes. For this model, a few dimensions transiently amplify the activity perturbations, albeit more weakly than in the $\{A^{cx}, B\}$ model. In the case of the $\{A^{cx}, B\}$ model, multiple dimensions strongly amplify the perturbation, whereas for the $\{A, B^{cx}\}$ model, there are no PC dimensions that result in transient amplification (Fig. 9E). Thus, the differential predictions we make for each of the three best model classes may disambiguate among the three different contextual decision-making mechanisms: purely input-driven, purely dynamics-driven, or a mixture of the two (with potential different relative contributions from each one), and can be readily implemented in future experiments.

# Supplementary Tables

**Table S1.** Monkey A data minimum LOOCV MSE ± sem across k folds (36 conditions) and corresponding latent dimensionality for which it is achieved, for LDS models with different input dimensionalities and contextual constraints. Highlighted in black is the model that achieved the minimum MSE. It cannot be appreciated in the 4th column (min MSE) due to rounding error, but can be seen in the last column (min Δ(MSE ± sem)), where performance is given relative to the best performing model (TFR 2D $AB^{cx}$ model, see Table S2), so the differences across models, albeit small, can be revealed. This quantity is the one reported in Fig. 2A.

| Input dim | Contextual constraints | Latent dim | min MSE | min ΔMSE ($\times 10^{-3}$) |
|---|---|---|---|---|
| 1D | $A^{cx}, B^{cx}$ | 13 | 0.74 ± 0.02 | 13 ± 1 |
| | $A^{cx}, B$ | 15 | 0.74 ± 0.02 | 13 ± 1 |
| | $A, B^{cx}$ | 15 | 0.74 ± 0.02 | 12 ± 1 |
| | $A, B$ | 16 | 0.78 ± 0.02 | 48 ± 6 |
| 2D | $A^{cx}, B^{cx}$ | 15 | 0.73 ± 0.02 | 2.7 ± 0.4 |
| | $A^{cx}, B$ | 15 | 0.73 ± 0.02 | 2.7 ± 0.4 |
| | $A, B^{cx}$ | 16 | 0.73 ± 0.02 | 1.7 ± 0.6 |
| | $A, B$ | 17 | 0.77 ± 0.02 | 38 ± 6 |
| 3D | $A^{cx}, B^{cx}$ | 14 | 0.73 ± 0.02 | 1.3 ± 0.4 |
| | $A^{cx}, B$ | 16 | 0.73 ± 0.02 | 1.3 ± 0.3 |
| | $\mathbf{A}, \mathbf{B^{cx}}$ | **18** | **0.73 ± 0.02** | **0.6 ± 0.5** |
| | $A, B$ | 17 | 0.77 ± 0.02 | 38 ± 6 |
| 4D | $A^{cx}, B^{cx}$ | 14 | 0.73 ± 0.02 | 1.9 ± 0.4 |
| | $A^{cx}, B$ | 14 | 0.73 ± 0.02 | 1.8 ± 0.4 |
| | $A, B^{cx}$ | 16 | 0.73 ± 0.02 | 1.0 ± 0.5 |
| | $A, B$ | 17 | 0.77 ± 0.02 | 39 ± 6 |

**Table S2.** Monkey A data minimum LOOCV MSE ± sem, min Δ(MSE ± sem) and corresponding latent dimensionality for which it is achieved, for TFR models with different input dimensionalities and contextual constraints. Same conventions as in Table S1. Performance in the last column is given relative to the best performing model (TFR 2D $AB^{cx}$ model).

| Input dim | Contextual constraints | Latent dim | min MSE | min $\Delta$MSE ($\times 10^{-3}$) |
|---|---|---|---|---|
| 1D | $AB^{cx}$ | 13 | 0.74 $\pm$ 0.02 | 9 $\pm$ 1 |
| | $AB$ | 14 | 0.78 $\pm$ 0.02 | 51 $\pm$ 7 |
| 2D | $\mathbf{AB^{cx}}$ | **14** | **0.73** $\pm$ **0.02** | **0** $\pm$ **0** |
| | $AB$ | 16 | 0.76 $\pm$ 0.02 | 36 $\pm$ 6 |
| 3D | $AB^{cx}$ | 14 | 0.73 $\pm$ 0.02 | 1.5 $\pm$ 0.4 |
| | $AB$ | 18 | 0.76 $\pm$ 0.02 | 36 $\pm$ 6 |
| 4D | $AB^{cx}$ | 14 | 0.73 $\pm$ 0.02 | 4.3 $\pm$ 0.5 |
| | $AB$ | 14 | 0.77 $\pm$ 0.02 | 38 $\pm$ 6 |

**Table S3.** Monkey F data minimum LOOCV MSE $\pm$ sem, min $\Delta$(MSE $\pm$ sem) and corresponding latent dimensionality for which it is achieved, for LDS models with different input dimensionalities and contextual constraints. Same conventions as in Table S1. Performance in the last column is given relative to the best performing model (TFR 2D $AB^{cx}$ model, see Table S4).

| Input dim | Contextual constraints | Latent dim | min MSE | min $\Delta$MSE ($\times 10^{-3}$) |
|---|---|---|---|---|
| 1D | $A^{cx}, B^{cx}$ | 12 | 0.75 ± 0.02 | 18 ± 2 |
| | $A^{cx}, B$ | 14 | 0.75 ± 0.02 | 19 ± 2 |
| | $A, B^{cx}$ | 12 | 0.75 ± 0.02 | 18 ± 2 |
| | $A, B$ | 15 | 0.77 ± 0.02 | 39 ± 4 |
| 2D | $A^{cx}, B^{cx}$ | 13 | 0.73 ± 0.02 | 3.1 ± 0.3 |
| | $A^{cx}, B$ | 14 | 0.73 ± 0.02 | 3.6 ± 0.4 |
| | $A, B^{cx}$ | 13 | 0.73 ± 0.02 | 3.0 ± 0.4 |
| | $A, B$ | 15 | 0.75 ± 0.03 | 25 ± 4 |
| 3D | $A^{cx}, B^{cx}$ | 13 | 0.73 ± 0.02 | 1.1 ± 0.5 |
| | $A^{cx}, B$ | 13 | 0.73 ± 0.02 | 1.8 ± 0.4 |
| | $\mathbf{A}, \mathbf{B}^{\mathbf{cx}}$ | **13** | **0.73 ± 0.02** | **0.6 ± 0.5** |
| | $A, B$ | 14 | 0.75 ± 0.03 | 24 ± 4 |
| 4D | $A^{cx}, B^{cx}$ | 13 | 0.73 ± 0.02 | 1.0 ± 0.4 |
| | $A^{cx}, B$ | 13 | 0.73 ± 0.02 | 1.2 ± 0.4 |
| | $A, B^{cx}$ | 12 | 0.73 ± 0.02 | 0.8 ± 0.4 |
| | $A, B$ | 13 | 0.75 ± 0.03 | 24 ± 4 |

**Table S4.** Monkey F data minimum LOOCV MSE ± sem, min Δ(MSE ± sem) and corresponding latent dimensionality for which it is achieved, for TFR models with different input dimensionalities and contextual constraints. Same conventions as in Table S1. Performance in the last column is given relative to the best performing model (TFR 2D $AB^{cx}$ model).

| Input dim | Contextual constraints | Latent dim | min MSE | min ΔMSE ($\times 10^{-3}$) |
|---|---|---|---|---|
| 1D | $AB^{cx}$ | 12 | 0.74 ± 0.02 | 12 ± 2 |
| | $AB$ | 13 | 0.77 ± 0.02 | 43 ± 4 |
| 2D | $\mathbf{AB^{cx}}$ | **12** | **0.73 ± 0.02** | **0 ± 0** |
| | $AB$ | 13 | 0.75 ± 0.03 | 23 ± 4 |
| 3D | $AB^{cx}$ | 12 | 0.73 ± 0.02 | 1.0 ± 0.3 |
| | $AB$ | 12 | 0.75 ± 0.03 | 22 ± 4 |
| 4D | $AB^{cx}$ | 12 | 0.73 ± 0.02 | 4.2 ± 0.5 |
| | $AB$ | 12 | 0.75 ± 0.03 | 24 ± 4 |

**Table S5.** RNN data minimum LOOCV MSE ± sem, min Δ(MSE ± sem) and corresponding latent dimensionality for which it is achieved, for LDS models with different input dimensionalities and contextual constraints. Same conventions as in Table S1. Performance in the last column is given relative to the best performing model (LDS 4D $A, B^{cx}$ model).

| Input dim | Contextual constraints | Latent dim | min MSE | min ΔMSE ($\times 10^{-3}$) |
|---|---|---|---|---|
| 1D | $A^{cx}, B$ | 23 | 0.039 ± 0.003 | 27 ± 2 |
| | $A, B^{cx}$ | 26 | 0.039 ± 0.003 | 27 ± 2 |
| 2D | $A^{cx}, B$ | 13 | 0.017 ± 0.001 | 5.1 ± 0.5 |
| | $A, B^{cx}$ | 26 | 0.017 ± 0.001 | 5.0 ± 0.6 |
| 3D | $A^{cx}, B$ | 14 | 0.013 ± 0.001 | 0.8 ± 0.3 |
| | $A, B^{cx}$ | 26 | 0.012 ± 0.001 | 0.1 ± 0.4 |
| 4D | $A^{cx}, B$ | 15 | 0.013 ± 0.001 | 0.6 ± 0.2 |
| | $\mathbf{A, B^{cx}}$ | **24** | **0.012 ± 0.001** | **0 ± 0** |

## REFERENCES AND NOTES


1. V. Mante, D. Sussillo, K. V. Shenoy, W. T. Newsome, Context-dependent computation by recurrent dynamics in prefrontal cortex. *Nature* **503**, 78–84 (2013).

2. M. Siegel, T. J. Buschman, E. K. Miller, Cortical information flow during flexible sensorimotor decisions. *Science* **348**, 1352–1355 (2015).

3. J. Fuster, *The Prefrontal Cortex* (Elsevier, 2015).

4. E. K. Miller, J. D. Cohen, An integrative theory of prefrontal cortex function. *Annu. Rev. Neurosci.* **24**, 167–202 (2001).

5. J. D. Wallis, K. C. Anderson, E. K. Miller, Single neurons in prefrontal cortex encode abstract rules. *Nature* **411**, 953–956 (2001).

6. J. Tanji, E. Hoshi, Role of the lateral prefrontal cortex in executive behavioral control. *Physiol. Rev.* **88**, 37–57 (2008).

7. M. J. Buckley, F. A. Mansouri, H. Hoda, M. Mahboubi, P. G. F. Browning, S. C. Kwok, A. Phillips, K. Tanaka, Dissociable components of rule-guided behavior depend on distinct medial and prefrontal regions. *Science* **325**, 52–58 (2009).

8. F. Katsuki, C. Constantinidis, Unique and shared roles of the posterior parietal and dorsolateral prefrontal cortex in cognitive functions. *Front. Integr. Neurosci.* **6**, 17 (2012).

9. M. Suzuki, J. Gottlieb, Distinct neural mechanisms of distractor suppression in the frontal and parietal lobe. *Nat. Neurosci.* **16**, 98–104 (2013).

10. W. T. Newsome, K. H. Britten, J. A. Movshon, Neuronal correlates of a perceptual decision. *Nature* **341**, 52–54 (1989).

11. J. Soldado Magraner, Linear Dynamics of Evidence Integration in Contextual Decision Making. thesis, University College London (2018).

12. M. C. Aoi, V. Mante, J. W. Pillow, Prefrontal cortex exhibits multidimensional dynamic encoding during decision-making. *Nat. Neurosci.* **23**, 1410–1420 (2020).

13. G. F. Elsayed, J. P. Cunningham, Structure in neural population recordings: An expected byproduct of simpler phenomena? *Nat. Neurosci.* **20**, 1310–1318 (2017).

14. C. Chandrasekaran, J. Soldado-Magraner, D. Peixoto, W. T. Newsome, K. V. Shenoy,M. Sahani, Brittleness in model selection analysis of single neuron firing rates. bioRxiv 430710 [Preprint] (2018). https://doi.org/10.1101/430710.

15. G. Okazawa, C. E. Hatch, A. Mancoo, C. K. Machens, R. Kiani, Representational geometry of perceptual decisions in the monkey parietal cortex. *Cell* **184**, 3748–3761.e18 (2021).

16. H. S. Seung, How the brain keeps the eyes still. *Proc. Natl. Acad. Sci. U.S.A.* **93**, 13339–13344 (1996).

17. B. K. Murphy, K. D. Miller, Balanced amplification: A new mechanism of selective amplification of neural activity patterns. *Neuron* **61**, 635–648 (2009).

18. G. Hennequin, T. P. Vogels, W. Gerstner, Non-normal amplification in random balanced neuronal networks. *Phys. Rev. E* **86**, 011909 (2012).

19. M. Rigotti, O. Barak, M. R. Warden, X.-J. Wang, N. D. Daw, E. K. Miller, S. Fusi, The importance of mixed selectivity in complex cognitive tasks. *Nature* **497**, 585–590 (2013).

20. F. Mastrogiuseppe, S. Ostojic, Linking connectivity, dynamics, and computations in low-rank recurrent neural networks. *Neuron* **99**, 609–623.e29 (2018).

21. A. Dubreuil, A. Valente, M. Beiran, F. Mastrogiuseppe, S. Ostojic, The role of population structure in computations through neural dynamics. *Nat. Neurosci.* **25**, 783–794 (2022).

22. A. Valente, J. W. Pillow, S. Ostojic, Extracting computational mechanisms from neural data using low-rank RNNs. *NeurIPS.* **35**, 24072–24086 (2022).

23. C. Langdon, T. A. Engel, Latent circuit inference from heterogeneous neural responses during cognitive tasks. bioRxiv 477431 [Preprint] (2022). https://doi.org/10.1101/2022.01.23.477431.

24. M. Pagan, V. D. Tang, M. C. Aoi, J. W. Pillow, V. Mante, D. Sussillo, C. D. Brody, A new theoretical framework jointly explains behavioral and neural variability across subjects performing flexible decision-making. bioRxiv 518207 [Preprint] (2022). https://doi.org/10.1101/2022.11.28.518207.

25. G. Baggio, V. Rutten, G. Hennequin, S. Zampieri, Efficient communication over complex dynamical networks: The role of matrix non-normality. *Sci. Adv.* **6**, eaba2282 (2020).

26. G. Bondanelli, S. Ostojic, Coding with transient trajectories in recurrent neural networks. *PLOS Comput. Biol.* **16**, e1007655 (2020).

27. J. P. Stroud, K. Watanabe, T. Suzuki, M. G. Stokes, M. Lengyel, Optimal information loading into working memory explains dynamic coding in the prefrontal cortex. *Proc. Natl. Acad. Sci. U.S.A.* **120**, e2307991120 (2023).

28. G. Christodoulou, T. P. Vogels, E. J. Agnes, Regimes and mechanisms of transient amplification in abstract and biological neural networks. *PLOS Comput. Biol.* **18**, e1010365 (2022).

29. M. S. Goldman, Memory without feedback in a neural network. *Neuron* **61**, 621–634 (2009).

30. G. Hennequin, T. P. Vogels, W. Gerstner, Optimal control of transient dynamics in balanced networks supports generation of complex movements. *Neuron* **82**, 1394–1406 (2014).

31. D. J. O'Shea, L. Duncker, W. Goo, X. Sun, S. Vyas, E. M. Trautmann, I. Diester, C. Ramakrishnan, K. Deisseroth, M. Sahani, K. V. Shenoy, Direct neural perturbations reveal a dynamical mechanism for robust computation. bioRxiv 520768 [Preprint] (2022). https://doi.org/10.1101/2022.12.16.520768.

32. A. Chadwick, A. G. Khan, J. Poort, A. Blot, S. B. Hofer, T. D. Mrsic-Flogel, M. Sahani, Learning shapes cortical dynamics to enhance integration of relevant sensory input. *Neuron* **111**, 106–120.e10 (2023).

33. S. Ardid, X.-J. Wang, A Tweaking principle for executive control: Neuronal circuit mechanism for rule-based task switching and conflict resolution. *J. Neurosci.* **33**, 19504–19517 (2013).

34. S. Treue, J. H. Maunsell, Attentional modulation of visual motion processing in cortical areas MT and MST. *Nature* **382**, 539–541 (1996).

35. S. Treue, J. C. M. Trujillo, Feature-based attention influences motion processing gain in macaque visual cortex. *Nature* **399**, 575–579 (1999).

36. S. Katzner, L. Busse, S. Treue, Attention to the color of a moving stimulus modulates motion-signal processing in macaque area MT: Evidence for a unified attentional system. *Front. Syst. Neurosci.* **3**, 12 (2009).

37. R. Sasaki, T. Uka, Dynamic readout of behaviorally relevant signals from area MT during task switching. *Neuron* **62**, 147–157 (2009).

38. G. Mirabella, G. Bertini, I. Samengo, B. E. Kilavik, D. Frilli, C. D. Libera, L. Chelazzi, Neurons in area V4 of the macaque translate attended visual features into behaviorally relevant categories. *Neuron* **54**, 303–318 (2007).

39. M. V. Bartsch, K. Loewe, C. Merkel, H.-J. Heinze, M. A. Schoenfeld, J. K. Tsotsos, J.-M. Hopf, Attention to color sharpens neural population tuning via feedback processing in the human visual cortex hierarchy. *J. Neurosci.* **37**, 10346–10357 (2017).

40. J. Barbosa, R. Proville, C. C. Rodgers, M. R. DeWeese, S. Ostojic, Y. Boubenec, Early selection of task-relevant features through population gating. *Nat. Commun.* **14**, 6837 (2023).

41. T. Flesch, K. Juechems, T. Dumbalska, A. Saxe, C. Summerfield, Orthogonal representations for robust context-dependent task performance in brains and neural networks. *Neuron* **110**, 1258–1270.e11 (2022).

42. M. Pagan, A. Valente, S. Ostojic, C. D. Brody, Brief technical note on linearizing recurrent neural networks (RNNs) before vs after the pointwise nonlinearity arXiv:2309.04030 [cs.LG] (2023).

43. C. Pandarinath, D. J. O'Shea, J. Collins, R. Jozefowicz, S. D. Stavisky, J. C. Kao, E. M. Trautmann, M. T. Kaufman, S. I. Ryu, L. R. Hochberg, J. M. Henderson, K. V. Shenoy, L. F. Abbott, D. Sussillo, Inferring single-trial neural population dynamics using sequential auto-encoders. *Nat. Methods* **15**, 805–815 (2018).

44. M. R. Keshtkaran, A. R. Sedler, R. H. Chowdhury, R. Tandon, D. Basrai, S. L. Nguyen, H. Sohn, M. Jazayeri, L. E. Miller, C. Pandarinath, A large-scale neural network training framework for generalized estimation of single-trial population dynamics. *Nat. Methods* **19**, 1572–1577 (2022).

45. E. L. Sylwestrak, Y. Jo, S. Vesuna, X. Wang, B. Holcomb, R. H. Tien, D. K. Kim, L. Fenno, C. Ramakrishnan, W. E. Allen, R. Chen, K. V. Shenoy, D. Sussillo, K. Deisseroth, Cell-type-specific population dynamics of diverse reward computations. *Cell* **185**, 3568–3587.e27 (2022).

46. M. G. Perich, C. Arlt, S. Soares, M. E. Young, C. P. Mosher, J. Minxha, E. Carter, U. Rutishauser, P. H. Rudebeck, C. D. Harvey, K. Rajan, Inferring brain-wide interactions using data-constrained recurrent neural network models. bioRxiv 423348 [Preprint] (2020). https://doi.org/10.1101/2020.12.18.423348.

47. T.-C. Kao, G. Hennequin, Neuroscience out of control: Control-theoretic perspectives on neural circuit dynamics. *Curr. Opin. Neurobiol.* **58**, 122–129 (2019).

48. T.-C. Kao, M. S. Sadabadi, G. Hennequin, Optimal anticipatory control as a theory of motor preparation: A thalamo-cortical circuit model. *Neuron* **109**, 1567–1581.e12 (2021).

49. M. Schimel, T.-C. Kao, K. T. Jensen, G. Hennequin, iLQR-VAE: Control-based learning of input-driven dynamics with applications to neural data. *ICLR* doi.org/10.1101/2021.10.07.463540, (2022).

50. P. J. Malonis, N. G. Hatsopoulos, J. N. MacLean, M. T. Kaufman, M1 dynamics share similar inputs for initiating and correcting movement. bioRxiv 464704 [Preprint] (2021). https://doi.org/10.1101/2021.10.18.464704.

51. A. R. Galgali, M. Sahani, V. Mante, Residual dynamics resolves recurrent contributions to neural computation. *Nat. Neurosci.* **26**, 326–338 (2023).

52. B. Feulner, M. G. Perich, R. H. Chowdhury, L. E. Miller, J. A. Gallego, C. Clopath, Small, correlated changes in synaptic connectivity may facilitate rapid motor learning. *Nat. Commun.* **13**, 5163 (2022).

53. S. Linderman, M. Johnson, A. Miller, R. Adams, D. Blei, L. Paninski, *Proceedings of the 20th International Conference on Artificial Intelligence and Statistics* (PMLR, 2017), pp. 914–922.

54. A. Nair, T. Karigo, B. Yang, S. Ganguli, M. J. Schnitzer, S. W. Linderman, D. J. Anderson, A. Kennedy, An approximate line attractor in the hypothalamus encodes an aggressive state. *Cell* **186**, 178–193.e15 (2023).

55. Y. Yang, S. Qiao, O. G. Sani, J. I. Sedillo, B. Ferrentino, B. Pesaran, M. M. Shanechi, Modelling and prediction of the dynamic responses of large-scale brain networks during direct electrical stimulation. *Nat. Biomed. Eng.* **5**, 324–345 (2021).

56. M. Schimel, T.-C. Kao, G. Hennequin, When and why does motor preparation arise in recurrent neural network models of motor control? *eLife* **12**, RP89131 (2024).

57. O. G. Sani, B. Pesaran, M. M. Shanechi, Where is all the nonlinearity: flexible nonlinear modeling of behaviorally relevant neural dynamics using recurrent neural networks. bioRxiv 458628 [Preprint] (2021). https://doi.org/10.1101/2021.09.03.458628.

58. M. Genkin, T. A. Engel, Moving beyond generalization to accurate interpretation of flexible models. *Nat. Mach. Intell.* **2**, 674–683 (2020).

59. J. DeFelipe, Brain plasticity and mental processes: Cajal again. *Nat. Rev. Neurosci.* **7**, 811–817 (2006).

60. C. K. Machens, R. Romo, C. D. Brody, Flexible control of mutual inhibition: A neural model of two-interval discrimination. *Science* **307**, 1121–1124 (2005).

61. M. M. Churchland, J. P. Cunningham, M. T. Kaufman, J. D. Foster, P. Nuyujukian, S. I. Ryu, K. V. Shenoy, Neural population dynamics during reaching. *Nature* **487**, 51–56 (2012).

62. K. V. Shenoy, M. Sahani, M. M. Churchland, Cortical control of arm movements: A dynamical systems perspective. *Annu. Rev. Neurosci.* **36**, 337–359 (2013).

63. E. D. Remington, S. W. Egger, D. Narain, J. Wang, M. Jazayeri, A dynamical systems perspective on flexible motor timing. *Trends Cogn. Sci.* **22**, 938–952 (2018).

64. E. Marder, D. Bucher, Central pattern generators and the control of rhythmic movements. *Curr. Biol.* **11**, R986–R996 (2001).

65. B. Hutcheon, Y. Yarom, Resonance, oscillation and the intrinsic frequency preferences of neurons. *Trends Neurosci.* **23**, 216–222 (2000).

66. S. Soldado-Magraner, F. Brandalise, S. Honnuraiah, M. Pfeiffer, M. Moulinier, U. Gerber, R. Douglas, Conditioning by subthreshold synaptic input changes the intrinsic firing pattern of CA3 hippocampal neurons. *J. Neurophysiol.* **123**, 90–106 (2019).

67. B. M. Yu, J. P. Cunningham, G. Santhanam, S. I. Ryu, K. V. Shenoy, M. Sahani, Gaussian-process factor analysis for low-dimensional single-trial analysis of neural population activity. *J. Neurophysiol.* **102**, 614–635 (2009).

68. L. Buesing, J. H. Macke, M. Sahani, Learning stable, regularised latent models of neural population dynamics. *Network* **23**, 24–47 (2012).

69. J. H. Macke, L. Buesing, J. P. Cunningham, B. M. Yu, K. V. Shenoy, M. Sahani, Empirical models of spiking in neural populations in *Advances in Neural Information Processing Systems 24*, (Curran Associates Inc., 2011) pp. 1350–1358.

70. D. Kobak, W. Brendel, C. Constantinidis, C. E. Feierstein, A. Kepecs, Z. F. Mainen, X.-L. Qi, R. Romo, N. Uchida, C. K. Machens, Demixed principal component analysis of neural population data. *eLife* **5**, e10989 (2016).

71. A. H. Williams, T. H. Kim, F. Wang, S. Vyas, S. I. Ryu, K. V. Shenoy, M. Schnitzer, T. G. Kolda, S. Ganguli, Unsupervised discovery of demixed, low-dimensional neural dynamics across multiple timescales through tensor component analysis. *Neuron* **98**, 1099–1115.e8 (2018).

72. O. Galor, *Discrete Dynamical Systems* (Springer, 2007).

73. M. Asllani, R. Lambiotte, T. Carletti, Structure and dynamical behavior of non-normal networks. *Sci. Adv.* **4**, eaau9403 (2018).

74. C. Libedinsky, M. Livingstone, Role of prefrontal cortex in conscious visual perception. *J. Neurosci.* **31**, 64–69 (2011).

75. G. F. Elsayed, A. H. Lara, M. T. Kaufman, M. M. Churchland, J. P. Cunningham, Reorganization between preparatory and movement population responses in motor cortex. *Nat. Commun.* **7**, 13239 (2016).

76. A. Valente, S. Ostojic, J. W. Pillow, Probing the relationship between latent linear dynamical systems and low-rank recurrent neural network models. *Neural Comput.* **34**, 1871–1892 (2022).
